# Supplementary material for: Contribution of CRISPRable DNA to human complex traits
Source: Commun Biol. 2022 Oct 20;5:1111. doi: 10.1038/s42003-022-03969-7 (PMC9585070; doi:10.1038/s42003-022-03969-7)
Supplement: Supplementary file 2 — Supplementary Information [file 42003_2022_3969_MOESM2_ESM.pdf]

Supplementary Information

for

Contribution of CRISPRable DNA to human complex traits

by Zhai et al.

Supplementary Figures

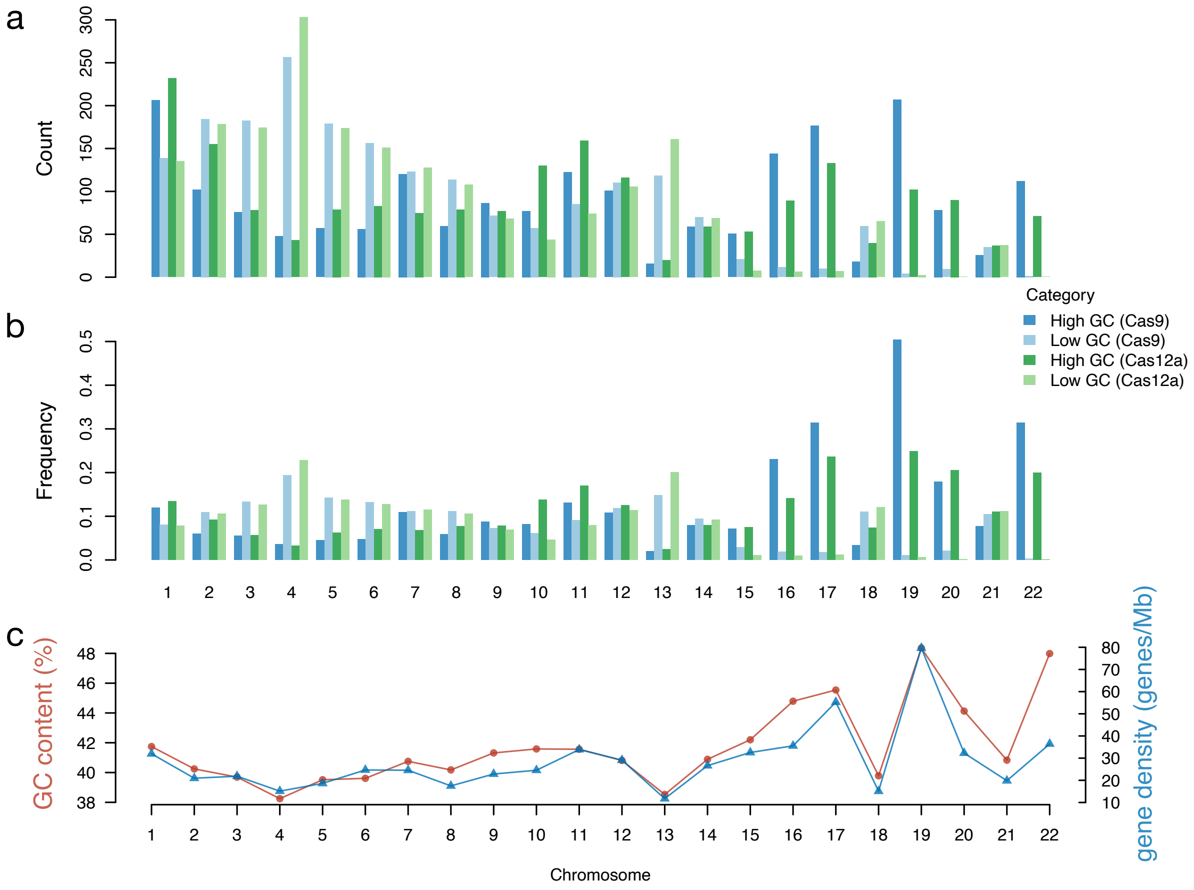

Supplementary Fig. 1: Distribution of Cas PAM-enriched regions, GC content, and gene density across 22 human autosomes. a,b. Cas was classified into four categories by their type (Cas9 or Cas12a) and GC content (see Fig. 1). High GC (Cas9) includes SpCas9, VRER-SpCas9, EQR-SpCas9, VQR-SpCas9, SpCas9-NG, xCas9, SpCas9-NRCH, SaCas9, St3Cas9, and SpaCas9. Low GC (Cas9) includes SpCas9-NRRH, SpCas9-NRTH, KKH-SaCas9, NmCas9, St1Cas9, and TdCas9. High GC (Cas12a) includes RR-AsCas12a. Low GC (Cas12a) includes AsCas12a, RVR-AsCas12a, enAsCas12a, and FnCas12a. **a** and **b** show the average count and frequency (count adjusted by chromosome length) distribution of top10% regions for each category, respectively. **c**. GC content and gene density distribution across 22 autosomes. GC content was based on the GRCh37 assembly fasta file, that is, the number of G and C bases divided by the number of total bases of each chromosome; and gene density is the number of genes divided by the number of total bases of each chromosome (in million base), gene number on each chromosome is from the UCSC known genes table (`https://genome.ucsc.edu/cgi-bin/hgTables`).

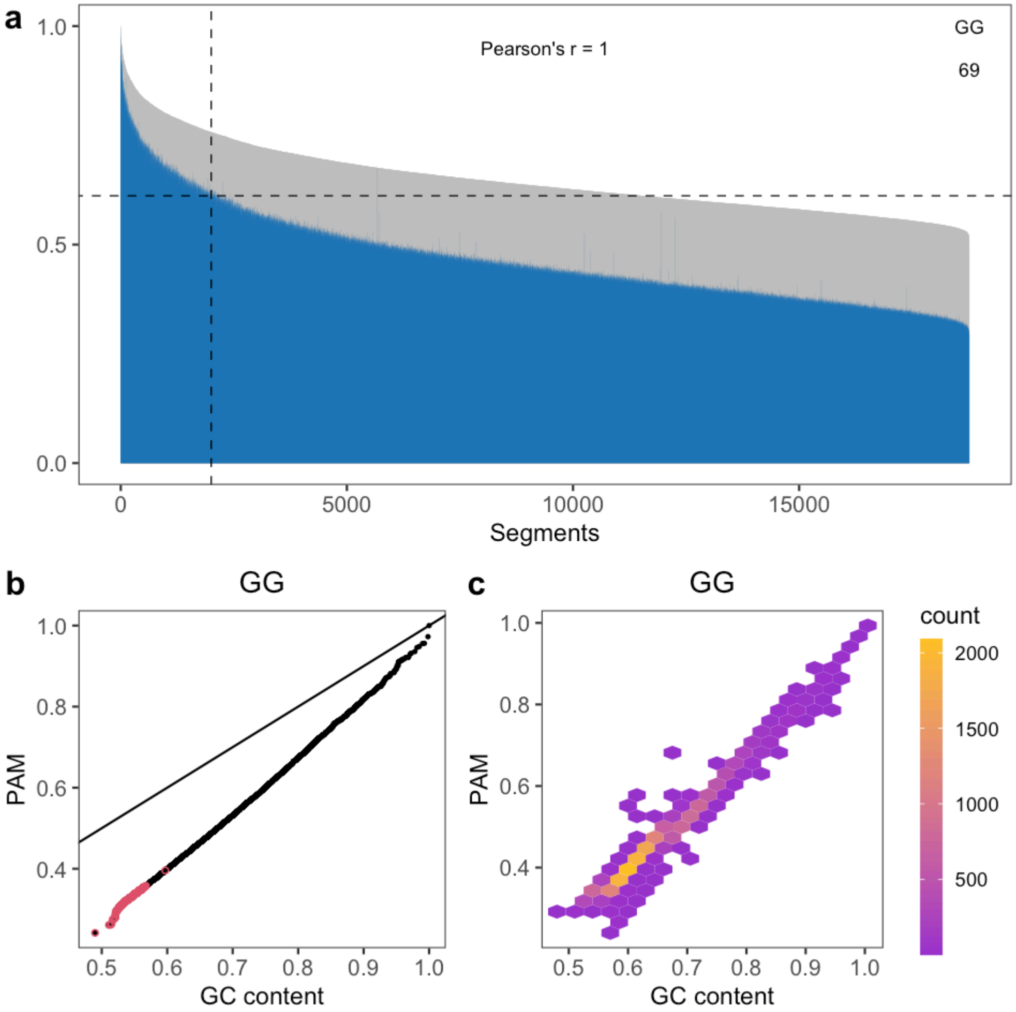

Supplementary Figure 2. Distribution of the GG PAM enriched regions compared with that of the GC-rich regions. a. PAM (shown on the top-right) content of 18,763 segments (excluding 1,237 segments with all N bases), respectively. The genome segments are ordered by decreasing GC content (grey), and the corresponding PAM content is plotted in the same order (blue). The segments to the left of the vertical dashed line are the top 2,000 segments for GC content, and the segments above the horizontal dashed line are the top 2,000 segments for the GG PAM. The number below the name of PAM (top-right) shows the number out of the top 2,000 PAM segments that are not in the top 2,000 GC-rich segments. The Pearson's r is the correlation between the GC content and the PAM content in all 18,763 segments. **b**. Quantile-quantile plot of the segments GC content v.s. PAM content distributions, where the red circles show the top 2,000 PAM segments, and the dashed line represents y = x. **c**. Hexbin plot of segments GC v.s. PAM content, where the color represents the count of points in each hexagon. GC and PAM content is normalized to the 0-1 range.

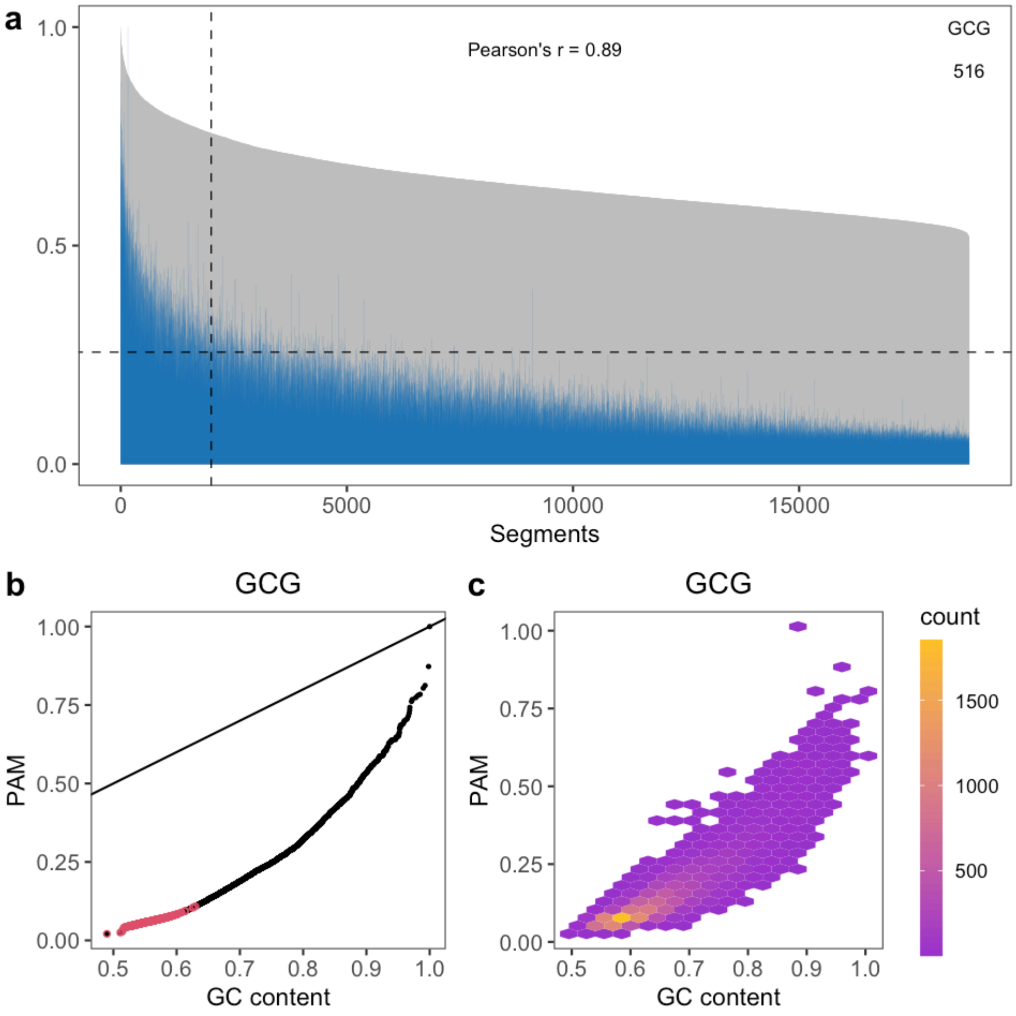

Supplementary Figure 3. Distribution of the GCG PAM enriched regions compared with that of the GC-rich regions. **a**. PAM (shown on the top-right) content of 18,763 segments (excluding 1,237 segments with all N bases), respectively. The genome segments are ordered by decreasing GC content (grey), and the corresponding PAM content is plotted in the same order (blue). The segments to the left of the vertical dashed line are the top 2,000 segments for GC content, and the segments above the horizontal dashed line are the top 2,000 segments for the GCG PAM. The number below the name of PAM (top-right) shows the number out of the top 2,000 PAM segments that are not in the top 2,000 GC-rich segments. The Pearson's r is the correlation between the GC content and the PAM content in all 18,763 segments. **b**. Quantile-quantile plot of the segments GC content v.s. PAM content distributions, where the red circles show the top 2,000 PAM segments, and the dashed line represents y = x. **c**. Hexbin plot of segments GC v.s. PAM content, where the color represents the count of points in each hexagon. GC and PAM content is normalized to the 0-1 range.

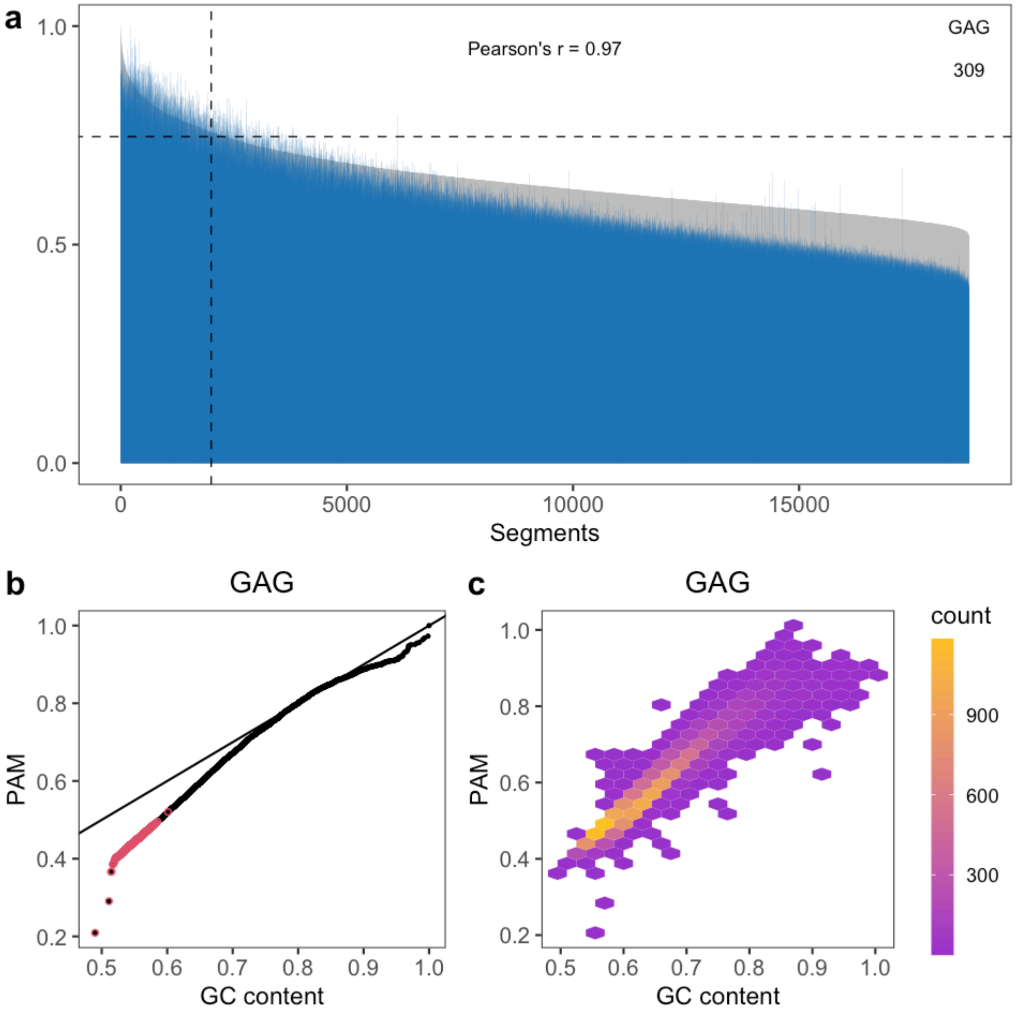

Supplementary Figure 4. Distribution of the GAG PAM enriched regions compared with that of the GC-rich regions. **a**. PAM (shown on the top-right) content of 18,763 segments (excluding 1,237 segments with all N bases), respectively. The genome segments are ordered by decreasing GC content (grey), and the corresponding PAM content is plotted in the same order (blue). The segments to the left of the vertical dashed line are the top 2,000 segments for GC content, and the segments above the horizontal dashed line are the top 2,000 segments for the GAG PAM. The number below the name of PAM (top-right) shows the number out of the top 2,000 PAM segments that are not in the top 2,000 GC-rich segments. The Pearson's r is the correlation between the GC content and the PAM content in all 18,763 segments. **b**. Quantile-quantile plot of the segments GC content v.s. PAM content distributions, where the red circles show the top 2,000 PAM segments, and the dashed line represents y = x. **c**. Hexbin plot of segments GC v.s. PAM content, where the color represents the count of points in each hexagon. GC and PAM content is normalized to the 0-1 range.

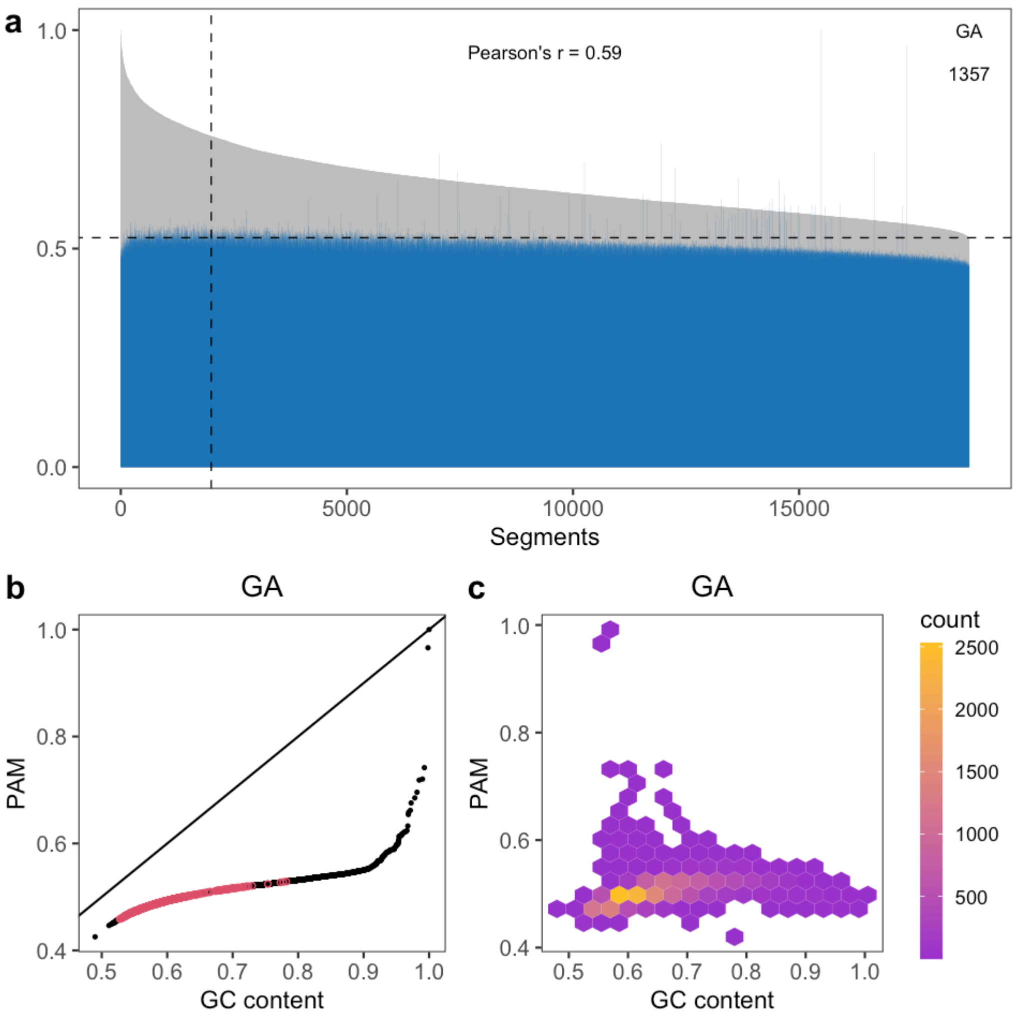

Supplementary Figure 5. Distribution of the GA PAM enriched regions compared with that of the GC-rich regions. **a**. PAM (shown on the top-right) content of 18,763 segments (excluding 1,237 segments with all N bases), respectively. The genome segments are ordered by decreasing GC content (grey), and the corresponding PAM content is plotted in the same order (blue). The segments to the left of the vertical dashed line are the top 2,000 segments for GC content, and the segments above the horizontal dashed line are the top 2,000 segments for the GA PAM. The number below the name of PAM (top-right) shows the number out of the top 2,000 PAM segments that are not in the top 2,000 GC-rich segments. The Pearson's r is the correlation between the GC content and the PAM content in all 18,763 segments. **b**. Quantile-quantile plot of the segments GC content v.s. PAM content distributions, where the red circles show the top 2,000 PAM segments, and the dashed line represents y = x. **c**. Hexbin plot of segments GC v.s. PAM content, where the color represents the count of points in each hexagon. GC and PAM content is normalized to the 0-1 range.

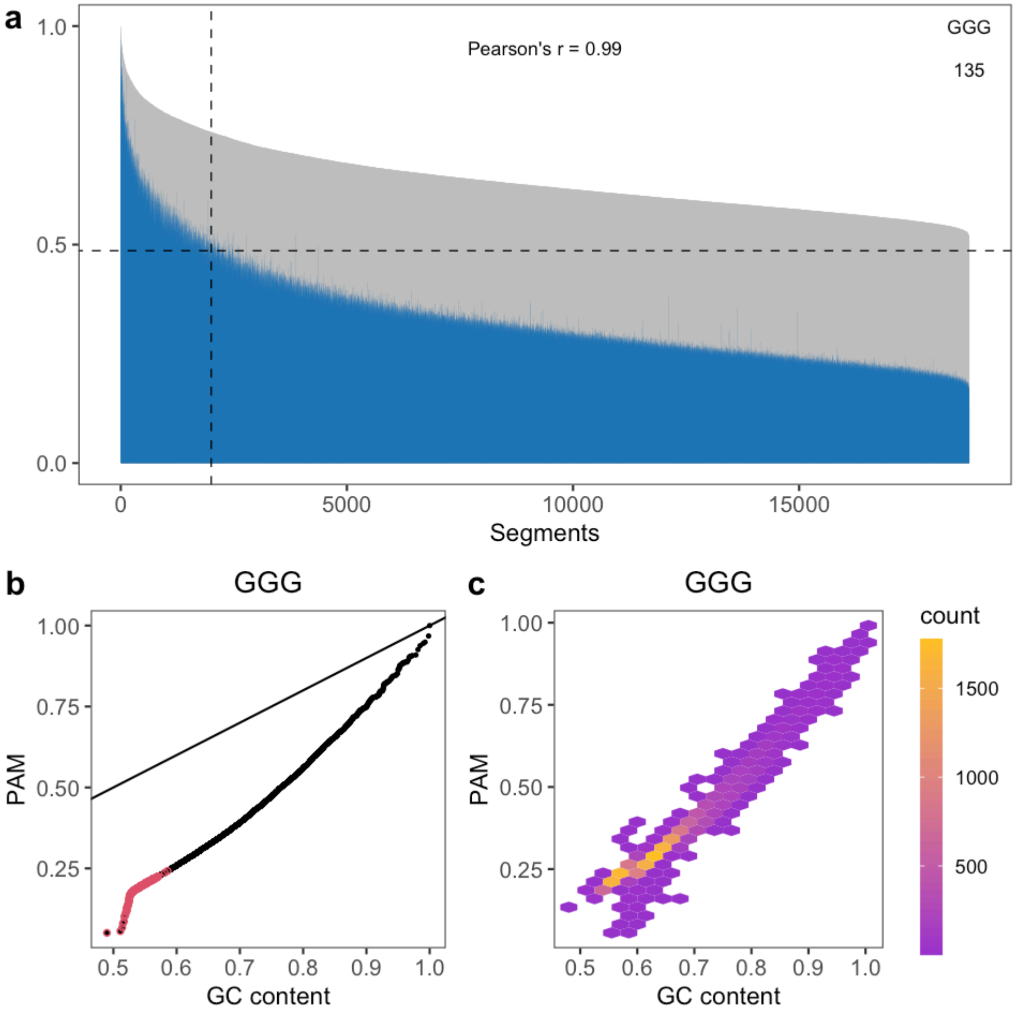

Supplementary Figure 6. Distribution of the GGG PAM enriched regions compared with that of the GC-rich regions. **a**. PAM (shown on the top-right) content of 18,763 segments (excluding 1,237 segments with all N bases), respectively. The genome segments are ordered by decreasing GC content (grey), and the corresponding PAM content is plotted in the same order (blue). The segments to the left of the vertical dashed line are the top 2,000 segments for GC content, and the segments above the horizontal dashed line are the top 2,000 segments for the GGG PAM. The number below the name of PAM (top-right) shows the number out of the top 2,000 PAM segments that are not in the top 2,000 GC-rich segments. The Pearson's r is the correlation between the GC content and the PAM content in all 18,763 segments. **b**. Quantile-quantile plot of the segments GC content v.s. PAM content distributions, where the red circles show the top 2,000 PAM segments, and the dashed line represents y = x. **c**. Hexbin plot of segments GC v.s. PAM content, where the color represents the count of points in each hexagon. GC and PAM content is normalized to the 0-1 range.

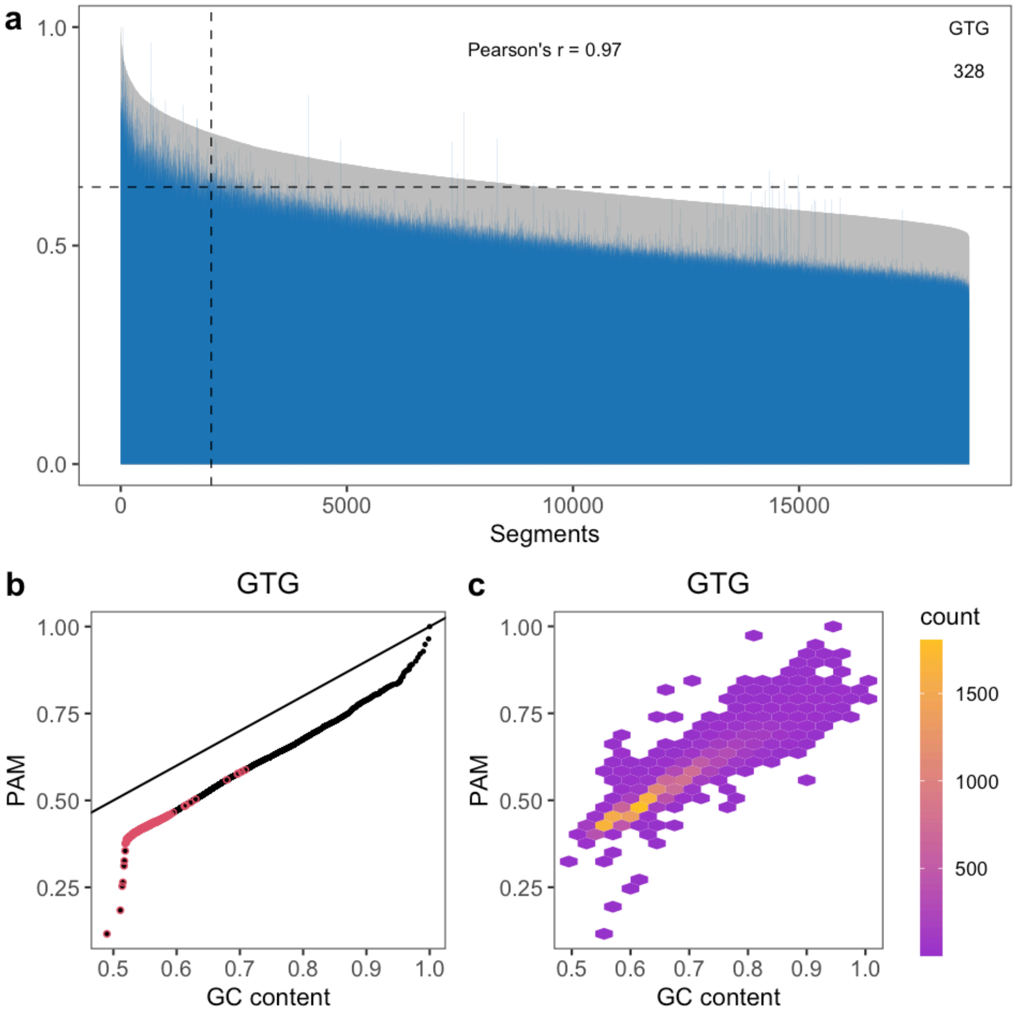

Supplementary Figure 7. Distribution of the GTG PAM enriched regions compared with that of the GC-rich regions. **a**. PAM (shown on the top-right) content of 18,763 segments (excluding 1,237 segments with all N bases), respectively. The genome segments are ordered by decreasing GC content (grey), and the corresponding PAM content is plotted in the same order (blue). The segments to the left of the vertical dashed line are the top 2,000 segments for GC content, and the segments above the horizontal dashed line are the top 2,000 segments for the GTG PAM. The number below the name of PAM (top-right) shows the number out of the top 2,000 PAM segments that are not in the top 2,000 GC-rich segments. The Pearson's r is the correlation between the GC content and the PAM content in all 18,763 segments. **b**. Quantile-quantile plot of the segments GC content v.s. PAM content distributions, where the red circles show the top 2,000 PAM segments, and the dashed line represents y = x. **c**. Hexbin plot of segments GC v.s. PAM content, where the color represents the count of points in each hexagon. GC and PAM content is normalized to the 0-1 range.

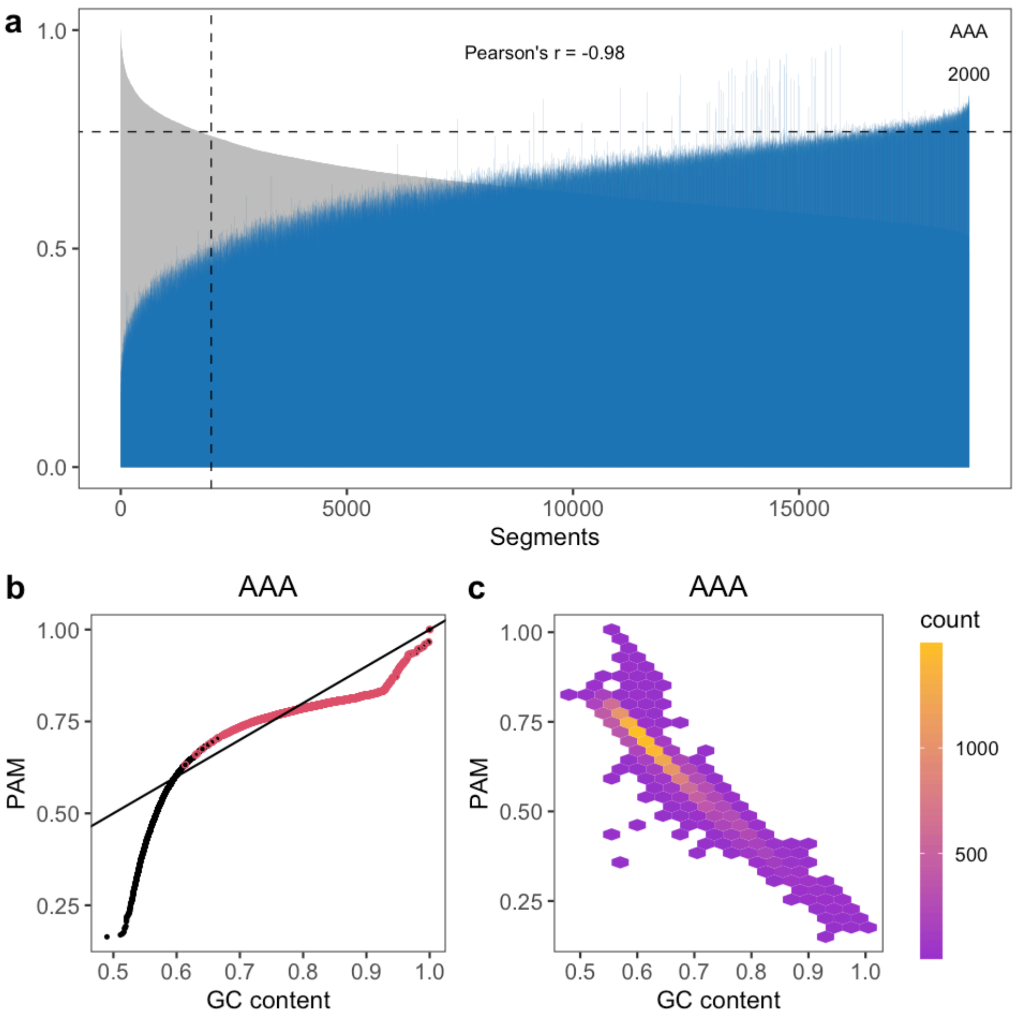

Supplementary Figure 8. Distribution of the AAA PAM enriched regions compared with that of the GC-rich regions. **a**. PAM (shown on the top-right) content of 18,763 segments (excluding 1,237 segments with all N bases), respectively. The genome segments are ordered by decreasing GC content (grey), and the corresponding PAM content is plotted in the same order (blue). The segments to the left of the vertical dashed line are the top 2,000 segments for GC content, and the segments above the horizontal dashed line are the top 2,000 segments for the AAA PAM. The number below the name of PAM (top-right) shows the number out of the top 2,000 PAM segments that are not in the top 2,000 GC-rich segments. The Pearson's r is the correlation between the GC content and the PAM content in all 18,763 segments. **b**. Quantile-quantile plot of the segments GC content v.s. PAM content distributions, where the red circles show the top 2,000 PAM segments, and the dashed line represents y = x. **c**. Hexbin plot of segments GC v.s. PAM content, where the color represents the count of points in each hexagon. GC and PAM content is normalized to the 0-1 range.

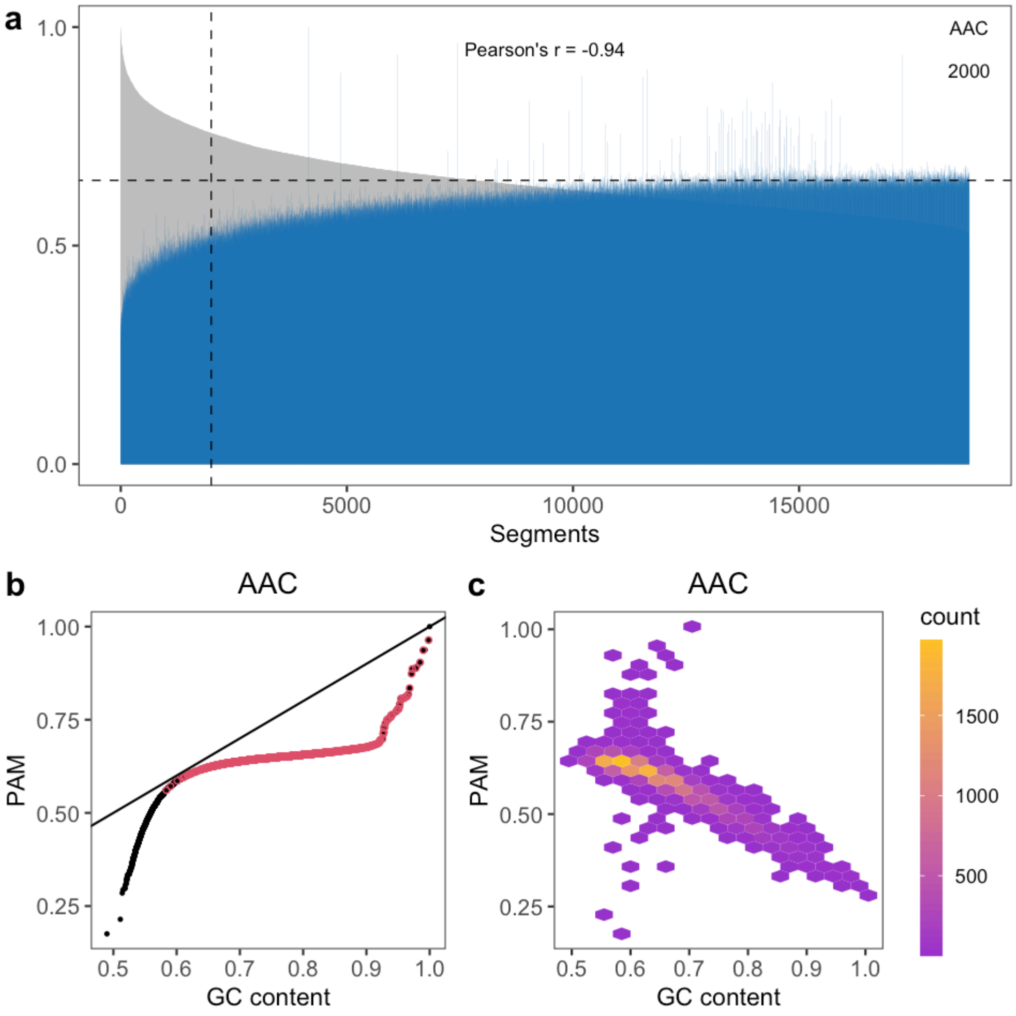

Supplementary Figure 9. Distribution of the AAC PAM enriched regions compared with that of the GC-rich regions. **a**. PAM (shown on the top-right) content of 18,763 segments (excluding 1,237 segments with all N bases), respectively. The genome segments are ordered by decreasing GC content (grey), and the corresponding PAM content is plotted in the same order (blue). The segments to the left of the vertical dashed line are the top 2,000 segments for GC content, and the segments above the horizontal dashed line are the top 2,000 segments for the AAC PAM. The number below the name of PAM (top-right) shows the number out of the top 2,000 PAM segments that are not in the top 2,000 GC-rich segments. The Pearson's r is the correlation between the GC content and the PAM content in all 18,763 segments. **b**. Quantile-quantile plot of the segments GC content v.s. PAM content distributions, where the red circles show the top 2,000 PAM segments, and the dashed line represents y = x. **c**. Hexbin plot of segments GC v.s. PAM content, where the color represents the count of points in each hexagon. GC and PAM content is normalized to the 0-1 range.

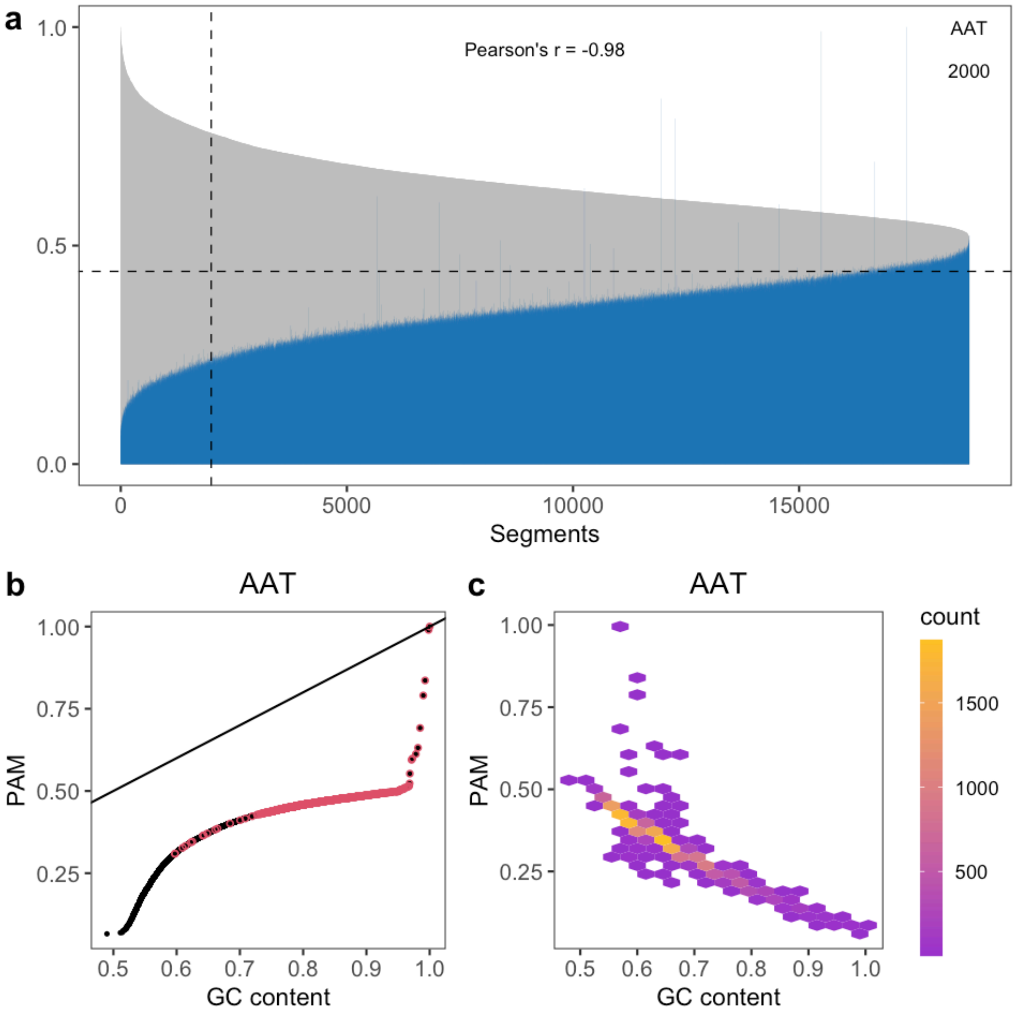

Supplementary Figure 10. Distribution of the AAT PAM enriched regions compared with that of the GC-rich regions. **a**. PAM (shown on the top-right) content of 18,763 segments (excluding 1,237 segments with all N bases), respectively. The genome segments are ordered by decreasing GC content (grey), and the corresponding PAM content is plotted in the same order (blue). The segments to the left of the vertical dashed line are the top 2,000 segments for GC content, and the segments above the horizontal dashed line are the top 2,000 segments for the AAT PAM. The number below the name of PAM (top-right) shows the number out of the top 2,000 PAM segments that are not in the top 2,000 GC-rich segments. The Pearson's r is the correlation between the GC content and the PAM content in all 18,763 segments. **b**. Quantile-quantile plot of the segments GC content v.s. PAM content distributions, where the red circles show the top 2,000 PAM segments, and the dashed line represents y = x. **c**. Hexbin plot of segments GC v.s. PAM content, where the color represents the count of points in each hexagon. GC and PAM content is normalized to the 0-1 range.

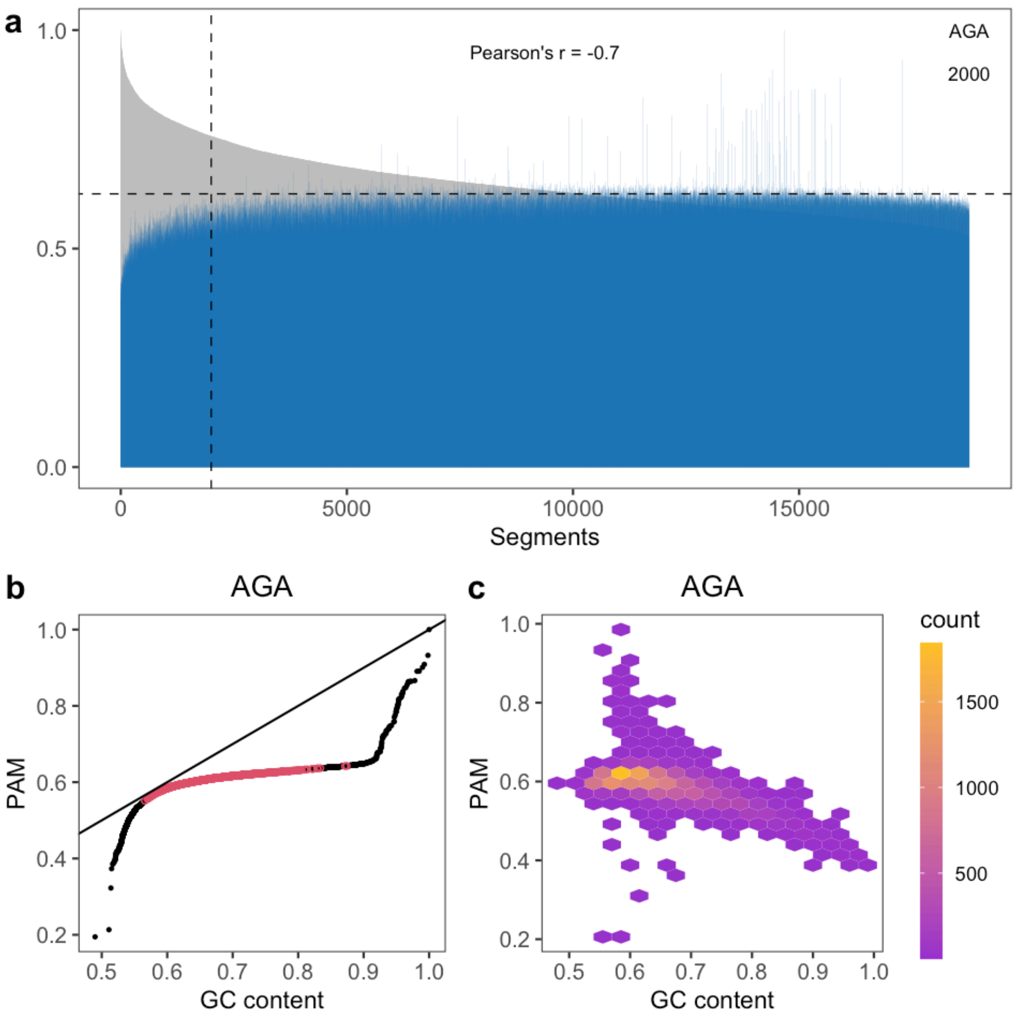

Supplementary Figure 11. Distribution of the AGA PAM enriched regions compared with that of the GC-rich regions. **a**. PAM (shown on the top-right) content of 18,763 segments (excluding 1,237 segments with all N bases), respectively. The genome segments are ordered by decreasing GC content (grey), and the corresponding PAM content is plotted in the same order (blue). The segments to the left of the vertical dashed line are the top 2,000 segments for GC content, and the segments above the horizontal dashed line are the top 2,000 segments for the AGA PAM. The number below the name of PAM (top-right) shows the number out of the top 2,000 PAM segments that are not in the top 2,000 GC-rich segments. The Pearson's r is the correlation between the GC content and the PAM content in all 18,763 segments. **b**. Quantile-quantile plot of the segments GC content v.s. PAM content distributions, where the red circles show the top 2,000 PAM segments, and the dashed line represents y = x. **c**. Hexbin plot of segments GC v.s. PAM content, where the color represents the count of points in each hexagon. GC and PAM content is normalized to the 0-1 range.

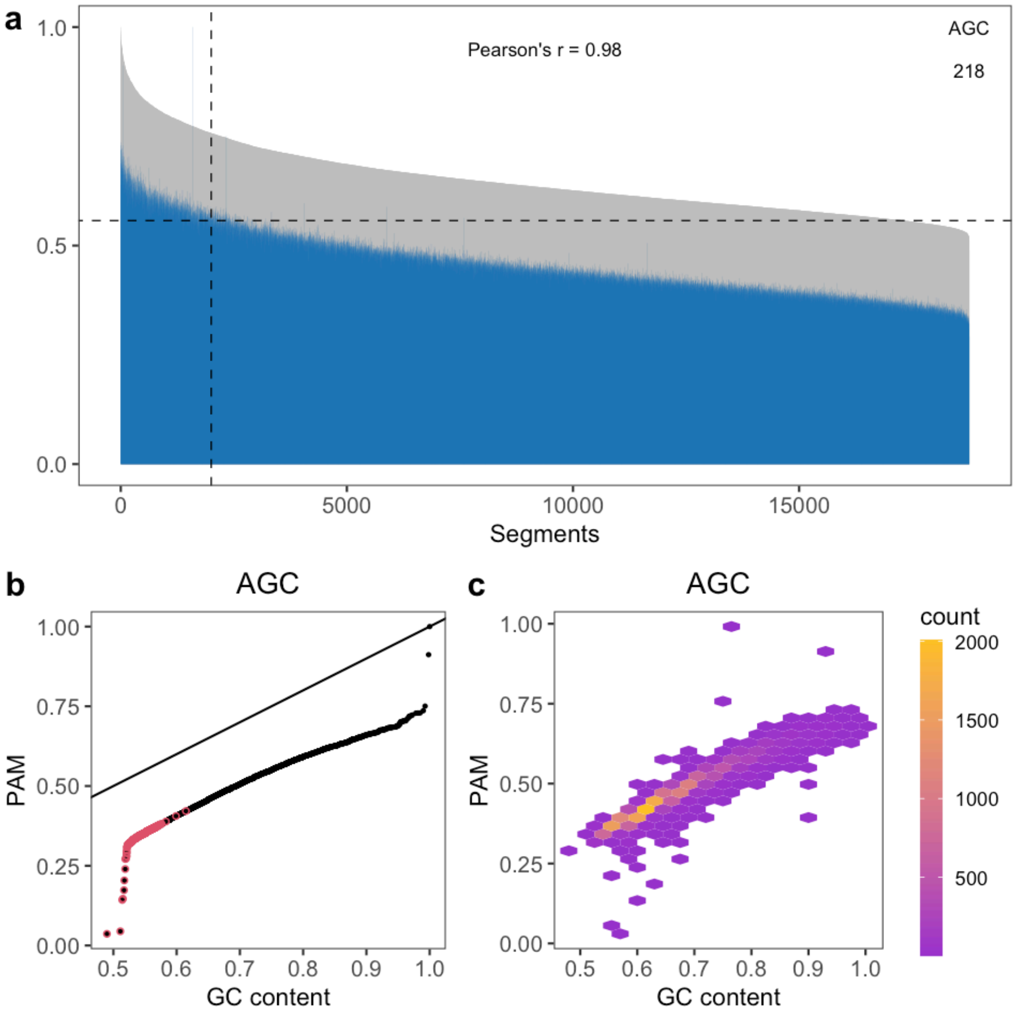

Supplementary Figure 12. Distribution of the AGC PAM enriched regions compared with that of the GC-rich regions. **a**. PAM (shown on the top-right) content of 18,763 segments (excluding 1,237 segments with all N bases), respectively. The genome segments are ordered by decreasing GC content (grey), and the corresponding PAM content is plotted in the same order (blue). The segments to the left of the vertical dashed line are the top 2,000 segments for GC content, and the segments above the horizontal dashed line are the top 2,000 segments for the AGC PAM. The number below the name of PAM (top-right) shows the number out of the top 2,000 PAM segments that are not in the top 2,000 GC-rich segments. The Pearson's r is the correlation between the GC content and the PAM content in all 18,763 segments. **b**. Quantile-quantile plot of the segments GC content v.s. PAM content distributions, where the red circles show the top 2,000 PAM segments, and the dashed line represents y = x. **c**. Hexbin plot of segments GC v.s. PAM content, where the color represents the count of points in each hexagon. GC and PAM content is normalized to the 0-1 range.

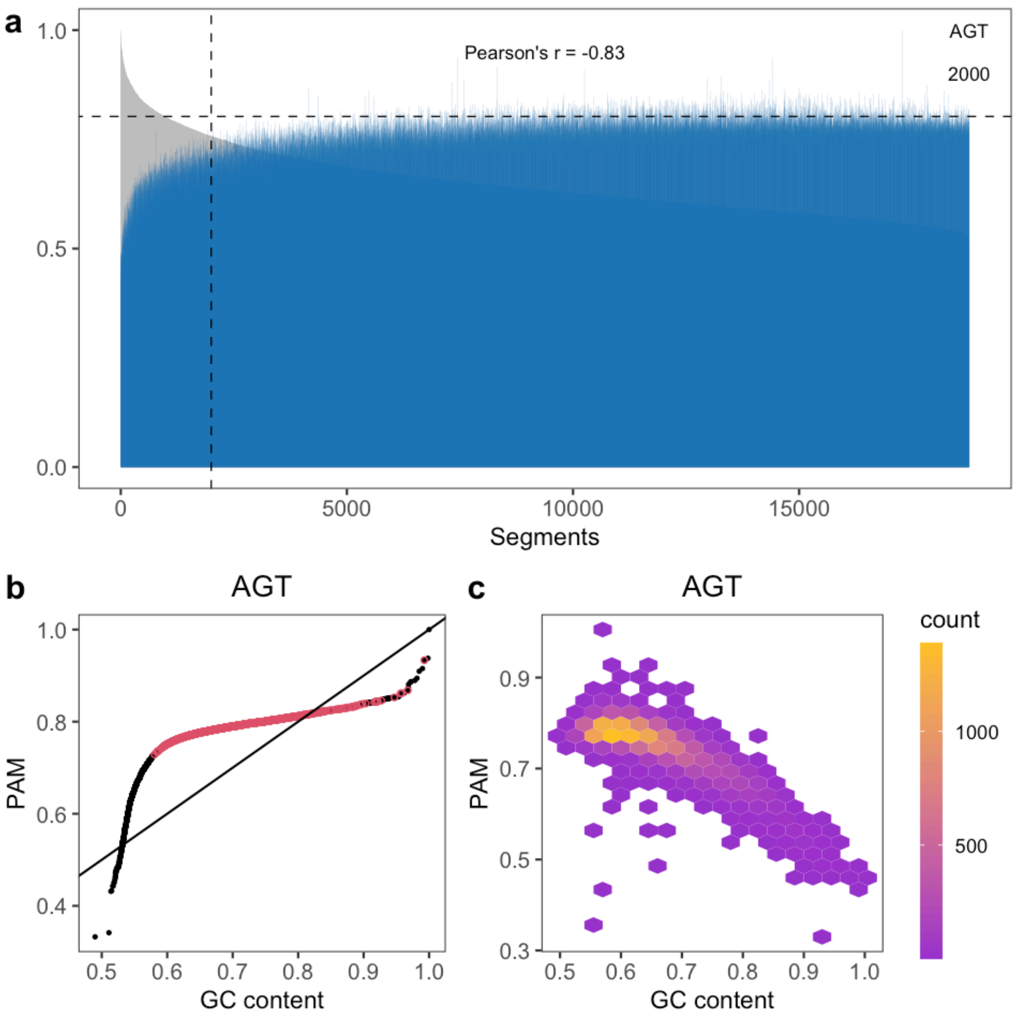

Supplementary Figure 13. Distribution of the AGT PAM enriched regions compared with that of the GC-rich regions. **a**. PAM (shown on the top-right) content of 18,763 segments (excluding 1,237 segments with all N bases), respectively. The genome segments are ordered by decreasing GC content (grey), and the corresponding PAM content is plotted in the same order (blue). The segments to the left of the vertical dashed line are the top 2,000 segments for GC content, and the segments above the horizontal dashed line are the top 2,000 segments for the AGT PAM. The number below the name of PAM (top-right) shows the number out of the top 2,000 PAM segments that are not in the top 2,000 GC-rich segments. The Pearson's r is the correlation between the GC content and the PAM content in all 18,763 segments. **b**. Quantile-quantile plot of the segments GC content v.s. PAM content distributions, where the red circles show the top 2,000 PAM segments, and the dashed line represents y = x. **c**. Hexbin plot of segments GC v.s. PAM content, where the color represents the count of points in each hexagon. GC and PAM content is normalized to the 0-1 range.

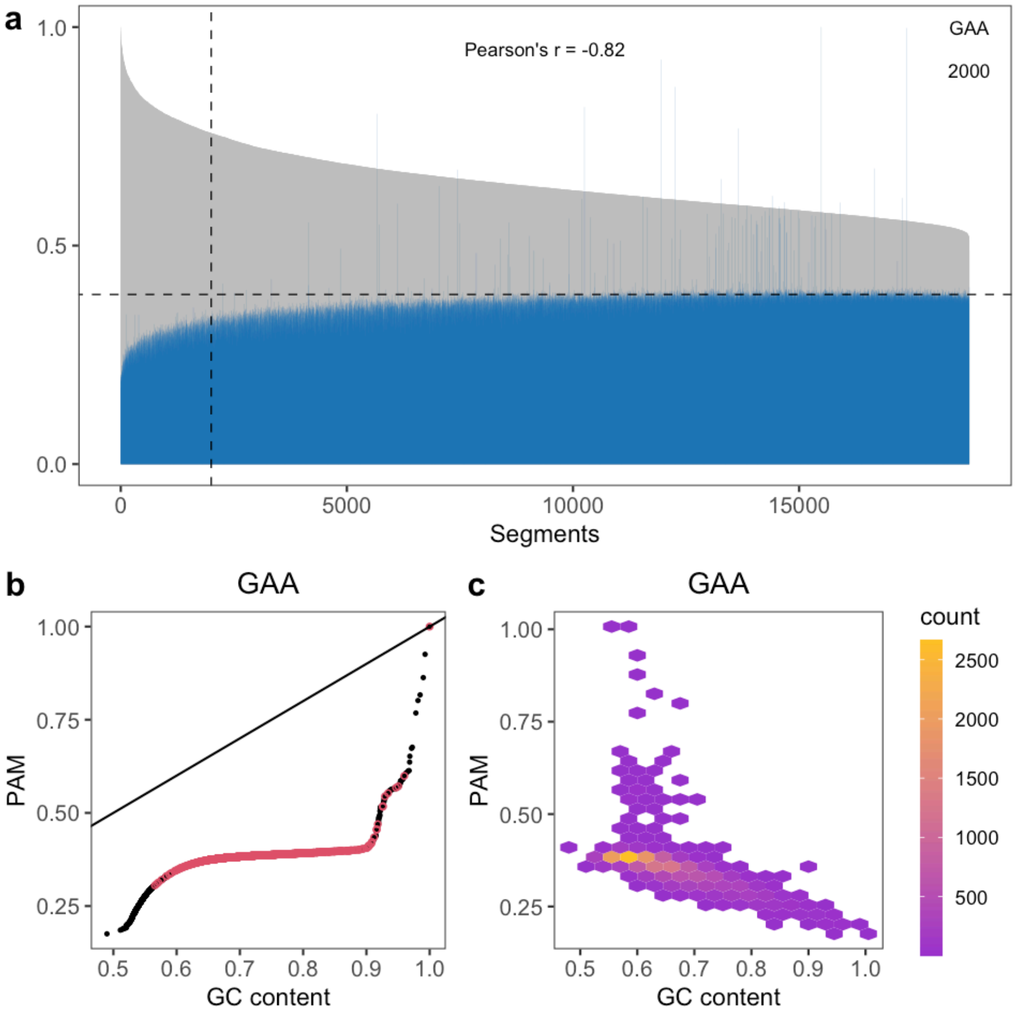

Supplementary Figure 14. Distribution of the GAA PAM enriched regions compared with that of the GC-rich regions. **a**. PAM (shown on the top-right) content of 18,763 segments (excluding 1,237 segments with all N bases), respectively. The genome segments are ordered by decreasing GC content (grey), and the corresponding PAM content is plotted in the same order (blue). The segments to the left of the vertical dashed line are the top 2,000 segments for GC content, and the segments above the horizontal dashed line are the top 2,000 segments for the GAA PAM. The number below the name of PAM (top-right) shows the number out of the top 2,000 PAM segments that are not in the top 2,000 GC-rich segments. The Pearson's r is the correlation between the GC content and the PAM content in all 18,763 segments. **b**. Quantile-quantile plot of the segments GC content v.s. PAM content distributions, where the red circles show the top 2,000 PAM segments, and the dashed line represents y = x. **c**. Hexbin plot of segments GC v.s. PAM content, where the color represents the count of points in each hexagon. GC and PAM content is normalized to the 0-1 range.

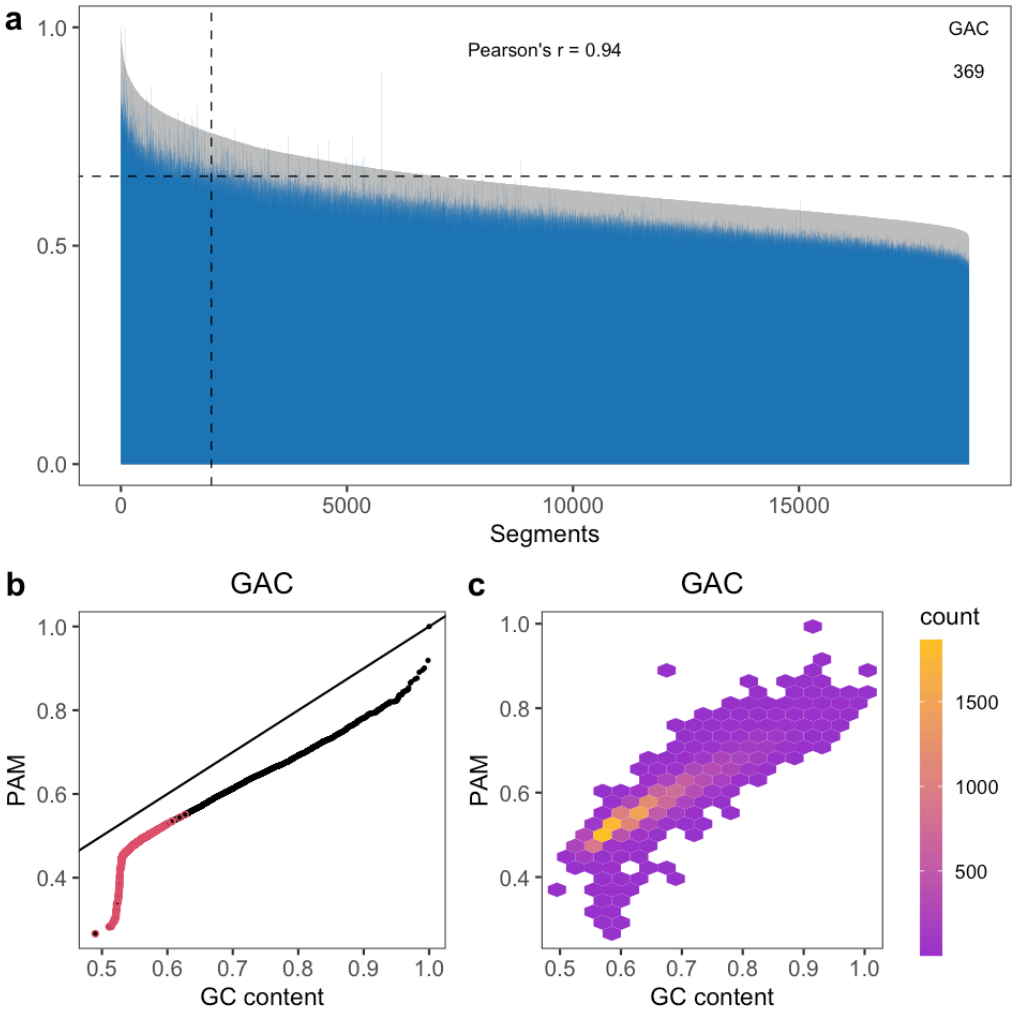

Supplementary Figure 15. Distribution of the GAC PAM enriched regions compared with that of the GC-rich regions. a. PAM (shown on the top-right) content of 18,763 segments (excluding 1,237 segments with all N bases), respectively. The genome segments are ordered by decreasing GC content (grey), and the corresponding PAM content is plotted in the same order (blue). The segments to the left of the vertical dashed line are the top 2,000 segments for GC content, and the segments above the horizontal dashed line are the top 2,000 segments for the GAC PAM. The number below the name of PAM (top-right) shows the number out of the top 2,000 PAM segments that are not in the top 2,000 GC-rich segments. The Pearson's r is the correlation between the GC content and the PAM content in all 18,763 segments. **b**. Quantile-quantile plot of the segments GC content v.s. PAM content distributions, where the red circles show the top 2,000 PAM segments, and the dashed line represents y = x. **c**. Hexbin plot of segments GC v.s. PAM content, where the color represents the count of points in each hexagon. GC and PAM content is normalized to the 0-1 range.

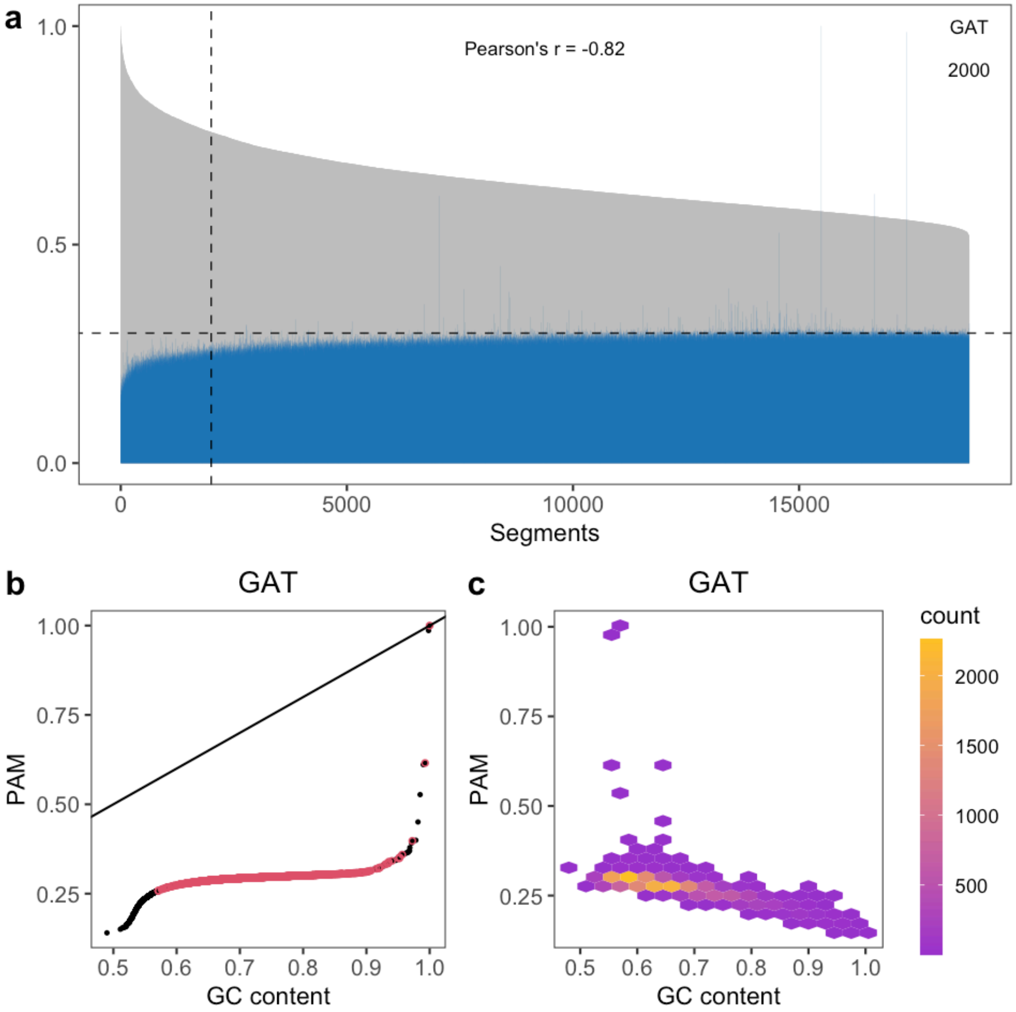

Supplementary Figure 16. Distribution of the GAT PAM enriched regions compared with that of the GC-rich regions. **a**. PAM (shown on the top-right) content of 18,763 segments (excluding 1,237 segments with all N bases), respectively. The genome segments are ordered by decreasing GC content (grey), and the corresponding PAM content is plotted in the same order (blue). The segments to the left of the vertical dashed line are the top 2,000 segments for GC content, and the segments above the horizontal dashed line are the top 2,000 segments for the GAT PAM. The number below the name of PAM (top-right) shows the number out of the top 2,000 PAM segments that are not in the top 2,000 GC-rich segments. The Pearson's r is the correlation between the GC content and the PAM content in all 18,763 segments. **b**. Quantile-quantile plot of the segments GC content v.s. PAM content distributions, where the red circles show the top 2,000 PAM segments, and the dashed line represents y = x. **c**. Hexbin plot of segments GC v.s. PAM content, where the color represents the count of points in each hexagon. GC and PAM content is normalized to the 0-1 range.

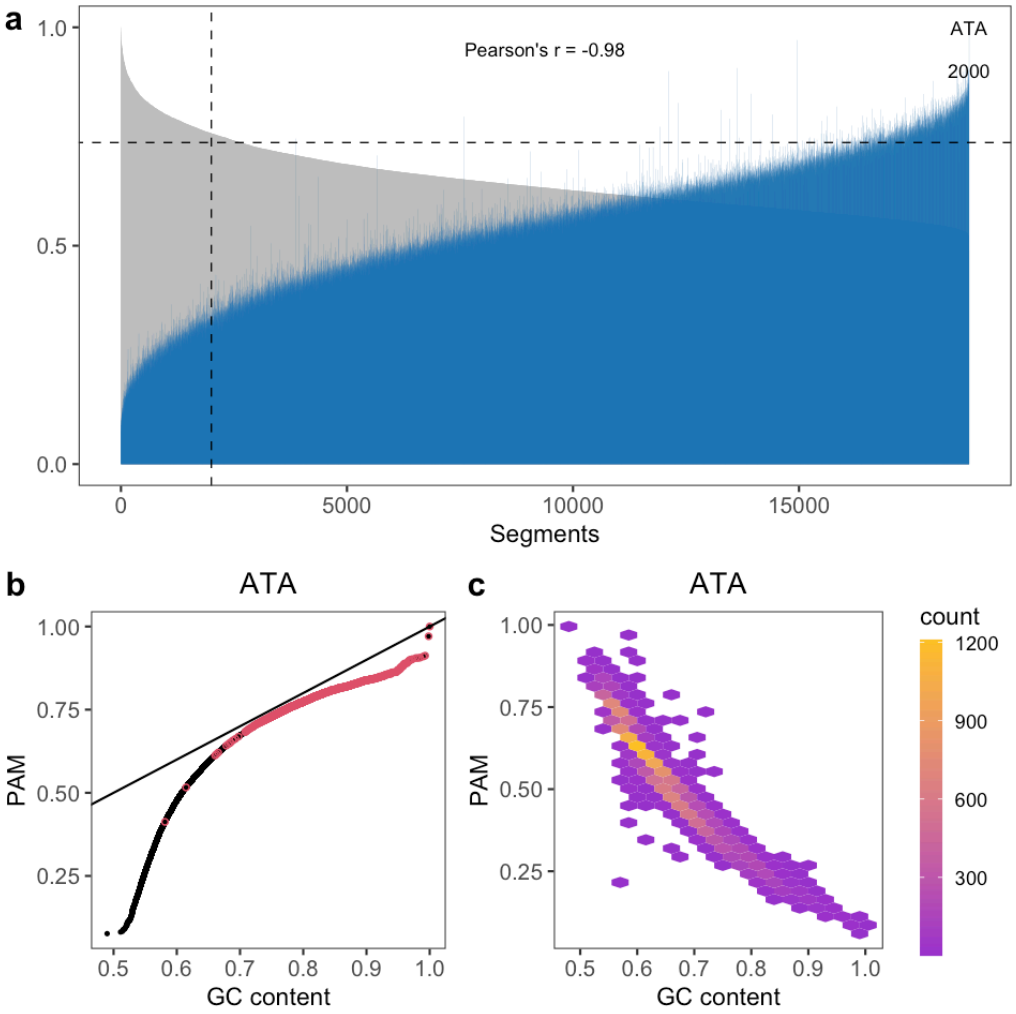

Supplementary Figure 17. Distribution of the ATA PAM enriched regions compared with that of the GC-rich regions. **a**. PAM (shown on the top-right) content of 18,763 segments (excluding 1,237 segments with all N bases), respectively. The genome segments are ordered by decreasing GC content (grey), and the corresponding PAM content is plotted in the same order (blue). The segments to the left of the vertical dashed line are the top 2,000 segments for GC content, and the segments above the horizontal dashed line are the top 2,000 segments for the ATA PAM. The number below the name of PAM (top-right) shows the number out of the top 2,000 PAM segments that are not in the top 2,000 GC-rich segments. The Pearson's r is the correlation between the GC content and the PAM content in all 18,763 segments. **b**. Quantile-quantile plot of the segments GC content v.s. PAM content distributions, where the red circles show the top 2,000 PAM segments, and the dashed line represents y = x. **c**. Hexbin plot of segments GC v.s. PAM content, where the color represents the count of points in each hexagon. GC and PAM content is normalized to the 0-1 range.

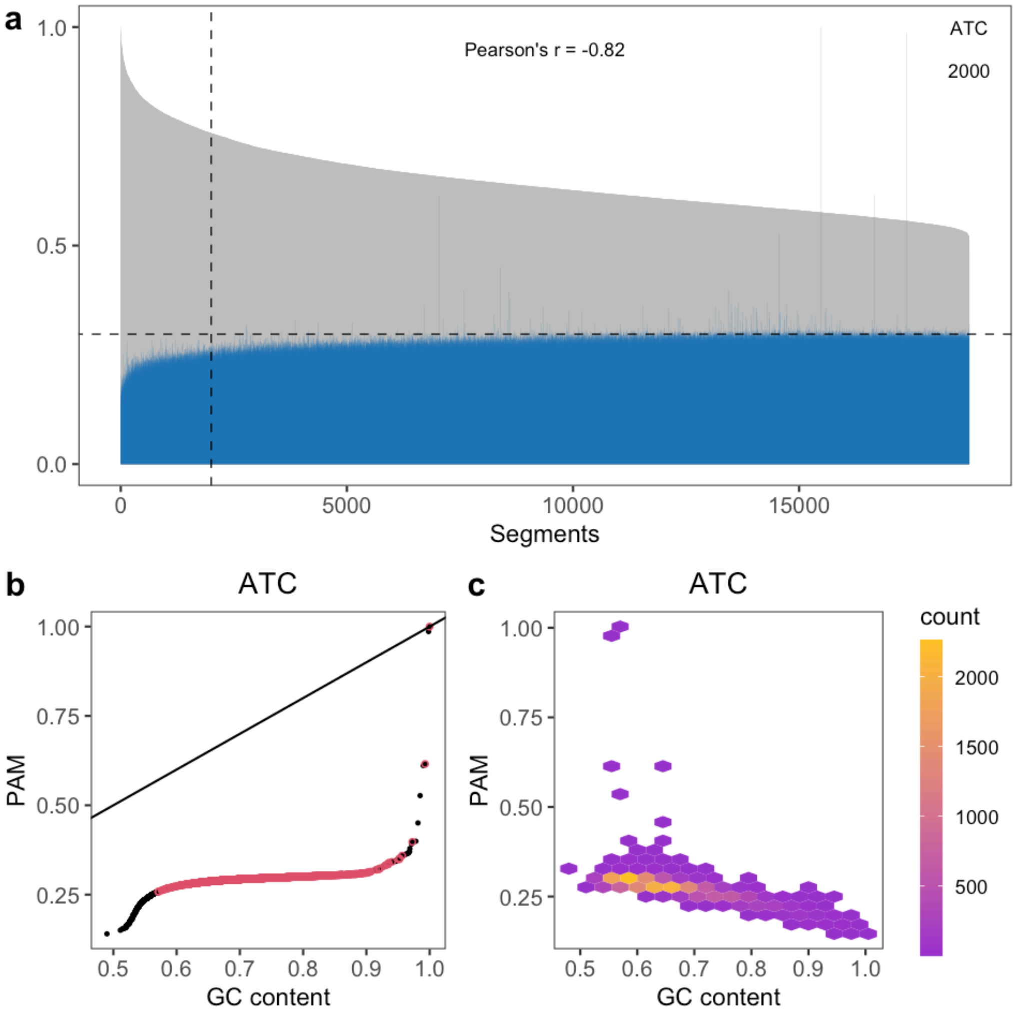

Supplementary Figure 18. Distribution of the ATC PAM enriched regions compared with that of the GC-rich regions. **a**. PAM (shown on the top-right) content of 18,763 segments (excluding 1,237 segments with all N bases), respectively. The genome segments are ordered by decreasing GC content (grey), and the corresponding PAM content is plotted in the same order (blue). The segments to the left of the vertical dashed line are the top 2,000 segments for GC content, and the segments above the horizontal dashed line are the top 2,000 segments for the ATC PAM. The number below the name of PAM (top-right) shows the number out of the top 2,000 PAM segments that are not in the top 2,000 GC-rich segments. The Pearson's r is the correlation between the GC content and the PAM content in all 18,763 segments. **b**. Quantile-quantile plot of the segments GC content v.s. PAM content distributions, where the red circles show the top 2,000 PAM segments, and the dashed line represents y = x. **c**. Hexbin plot of segments GC v.s. PAM content, where the color represents the count of points in each hexagon. GC and PAM content is normalized to the 0-1 range.

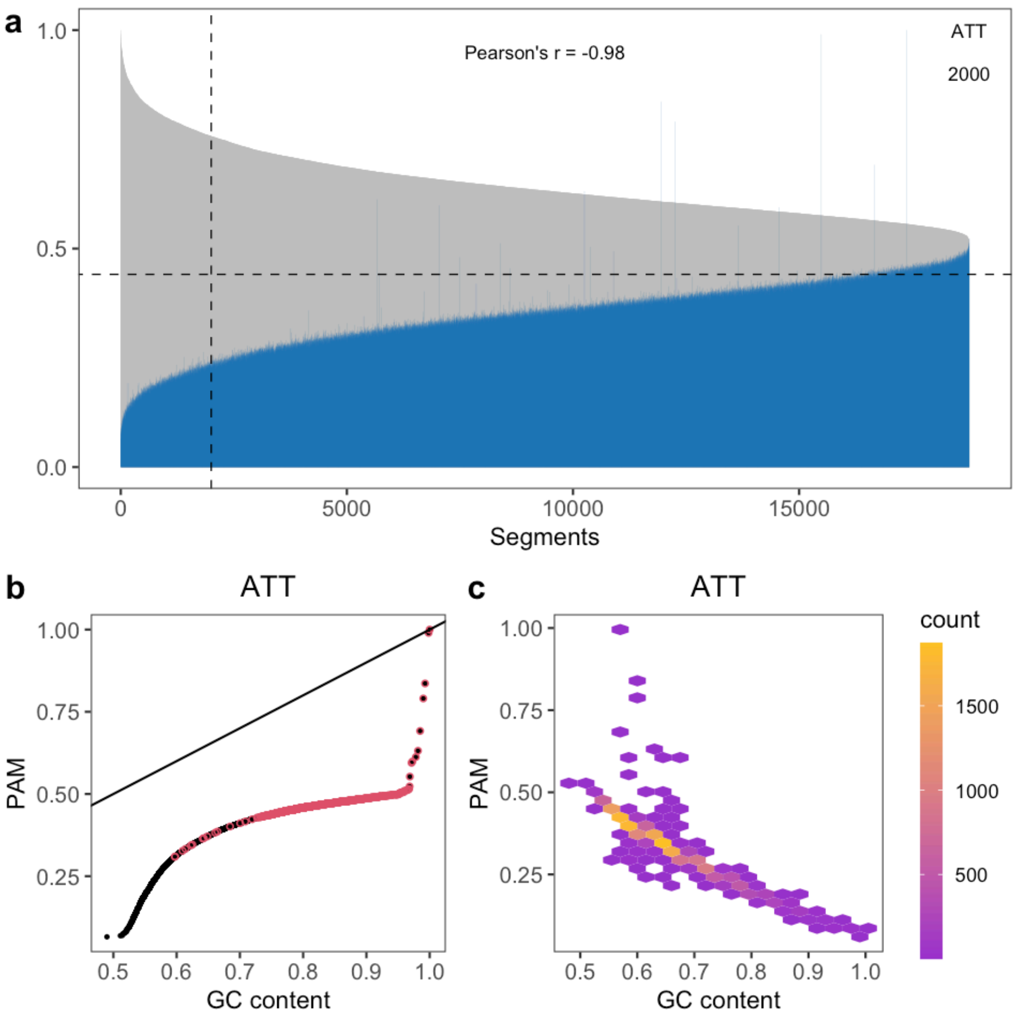

Supplementary Figure 19. Distribution of the ATT PAM enriched regions compared with that of the GC-rich regions. **a**. PAM (shown on the top-right) content of 18,763 segments (excluding 1,237 segments with all N bases), respectively. The genome segments are ordered by decreasing GC content (grey), and the corresponding PAM content is plotted in the same order (blue). The segments to the left of the vertical dashed line are the top 2,000 segments for GC content, and the segments above the horizontal dashed line are the top 2,000 segments for the ATT PAM. The number below the name of PAM (top-right) shows the number out of the top 2,000 PAM segments that are not in the top 2,000 GC-rich segments. The Pearson's r is the correlation between the GC content and the PAM content in all 18,763 segments. **b**. Quantile-quantile plot of the segments GC content v.s. PAM content distributions, where the red circles show the top 2,000 PAM segments, and the dashed line represents y = x. **c**. Hexbin plot of segments GC v.s. PAM content, where the color represents the count of points in each hexagon. GC and PAM content is normalized to the 0-1 range.

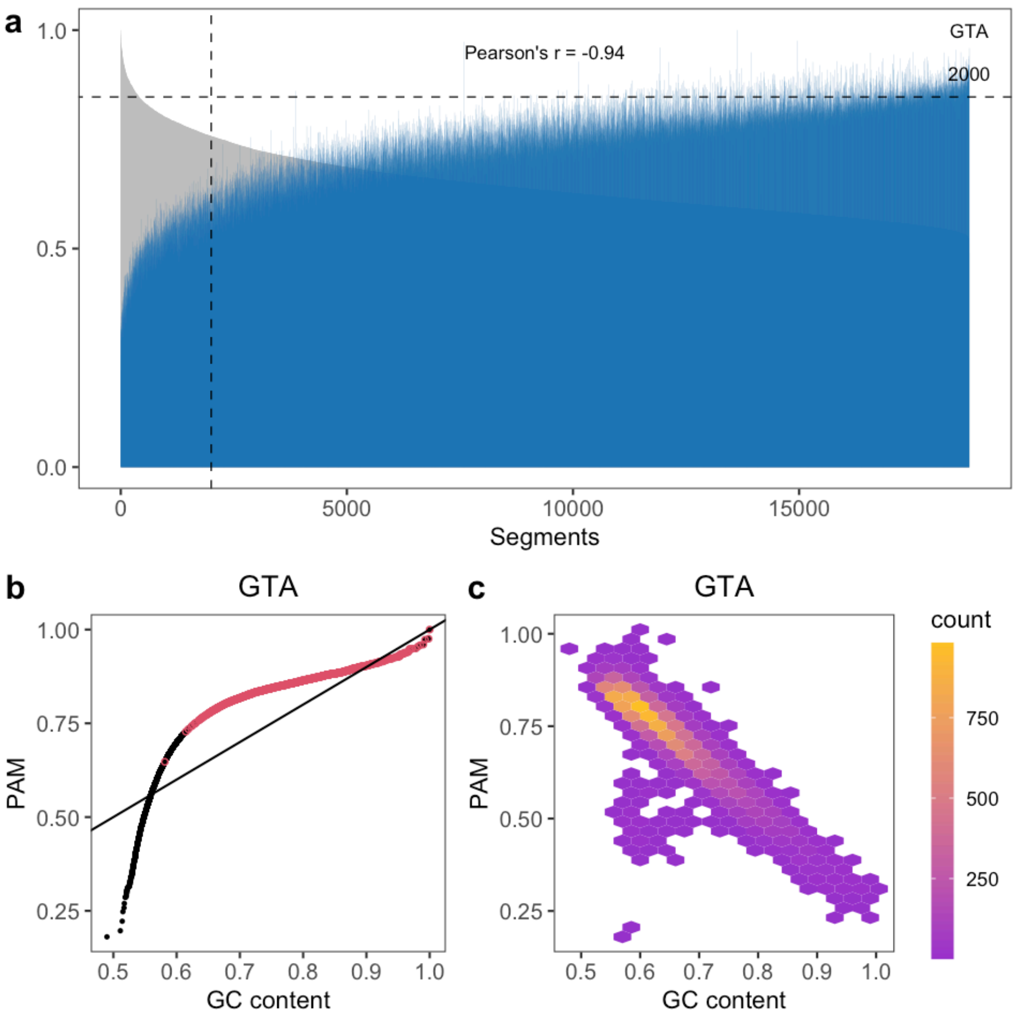

Supplementary Figure 20. Distribution of the GTA PAM enriched regions compared with that of the GC-rich regions. **a**. PAM (shown on the top-right) content of 18,763 segments (excluding 1,237 segments with all N bases), respectively. The genome segments are ordered by decreasing GC content (grey), and the corresponding PAM content is plotted in the same order (blue). The segments to the left of the vertical dashed line are the top 2,000 segments for GC content, and the segments above the horizontal dashed line are the top 2,000 segments for the GTA PAM. The number below the name of PAM (top-right) shows the number out of the top 2,000 PAM segments that are not in the top 2,000 GC-rich segments. The Pearson's r is the correlation between the GC content and the PAM content in all 18,763 segments. **b**. Quantile-quantile plot of the segments GC content v.s. PAM content distributions, where the red circles show the top 2,000 PAM segments, and the dashed line represents y = x. **c**. Hexbin plot of segments GC v.s. PAM content, where the color represents the count of points in each hexagon. GC and PAM content is normalized to the 0-1 range.

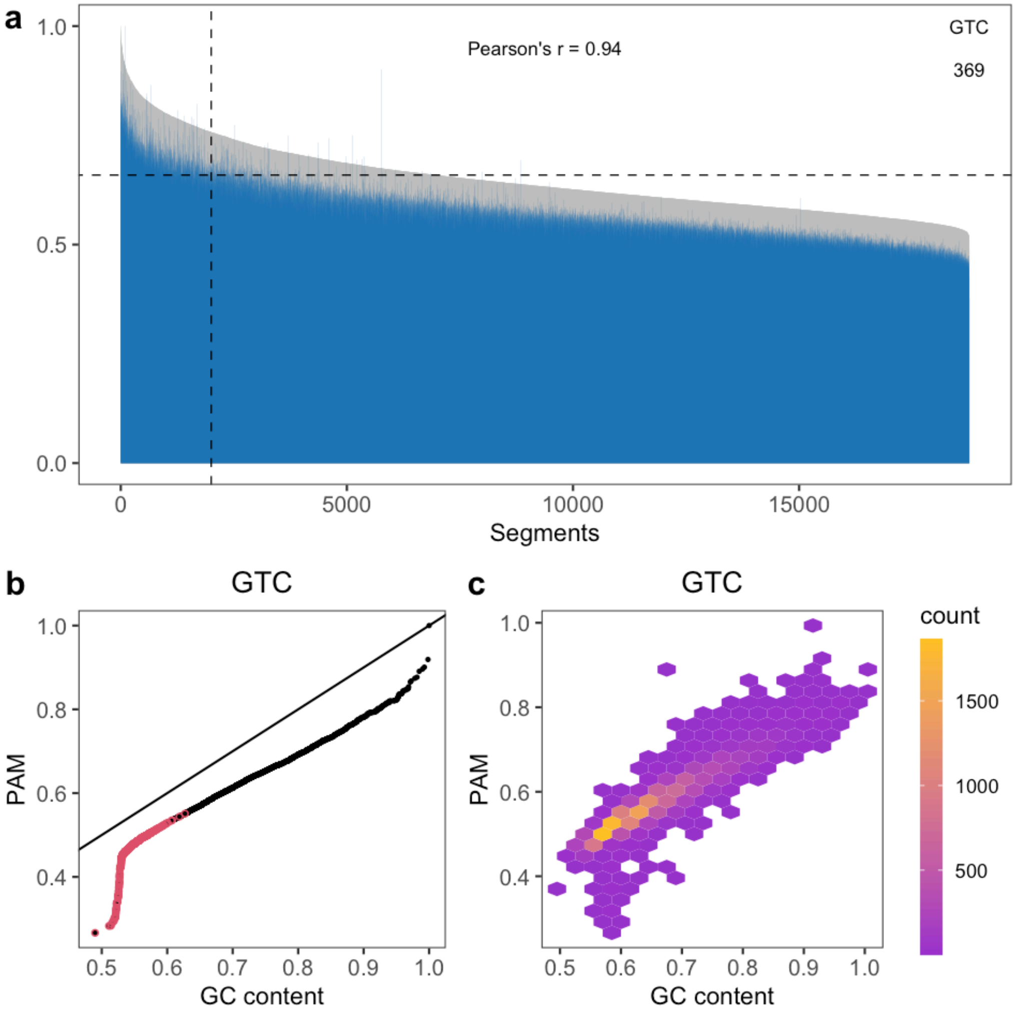

Supplementary Figure 21. Distribution of the GTC PAM enriched regions compared with that of the GC-rich regions. **a**. PAM (shown on the top-right) content of 18,763 segments (excluding 1,237 segments with all N bases), respectively. The genome segments are ordered by decreasing GC content (grey), and the corresponding PAM content is plotted in the same order (blue). The segments to the left of the vertical dashed line are the top 2,000 segments for GC content, and the segments above the horizontal dashed line are the top 2,000 segments for the GTC PAM. The number below the name of PAM (top-right) shows the number out of the top 2,000 PAM segments that are not in the top 2,000 GC-rich segments. The Pearson's r is the correlation between the GC content and the PAM content in all 18,763 segments. **b**. Quantile-quantile plot of the segments GC content v.s. PAM content distributions, where the red circles show the top 2,000 PAM segments, and the dashed line represents y = x. **c**. Hexbin plot of segments GC v.s. PAM content, where the color represents the count of points in each hexagon. GC and PAM content is normalized to the 0-1 range.

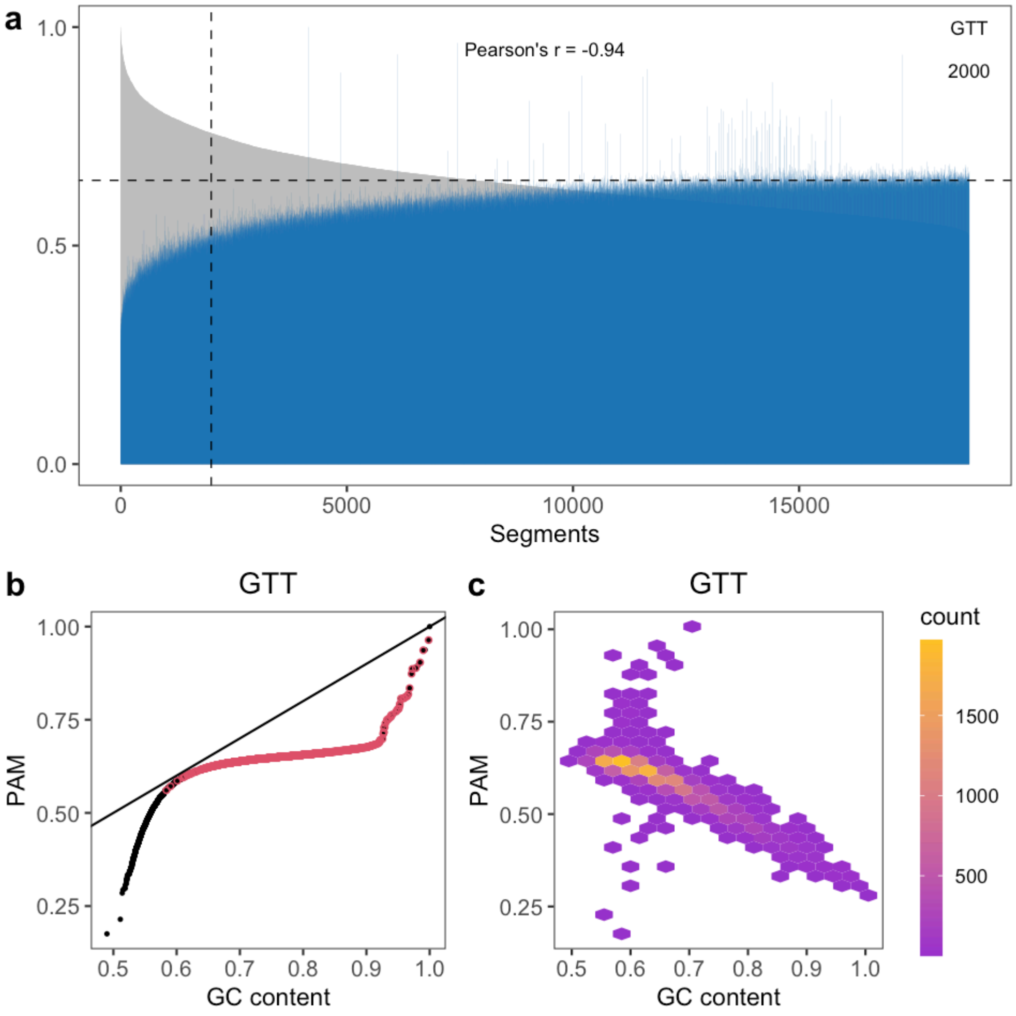

Supplementary Figure 22. Distribution of the GTT PAM enriched regions compared with that of the GC-rich regions. **a**. PAM (shown on the top-right) content of 18,763 segments (excluding 1,237 segments with all N bases), respectively. The genome segments are ordered by decreasing GC content (grey), and the corresponding PAM content is plotted in the same order (blue). The segments to the left of the vertical dashed line are the top 2,000 segments for GC content, and the segments above the horizontal dashed line are the top 2,000 segments for the GTT PAM. The number below the name of PAM (top-right) shows the number out of the top 2,000 PAM segments that are not in the top 2,000 GC-rich segments. The Pearson's r is the correlation between the GC content and the PAM content in all 18,763 segments. **b**. Quantile-quantile plot of the segments GC content v.s. PAM content distributions, where the red circles show the top 2,000 PAM segments, and the dashed line represents y = x. **c**. Hexbin plot of segments GC v.s. PAM content, where the color represents the count of points in each hexagon. GC and PAM content is normalized to the 0-1 range.

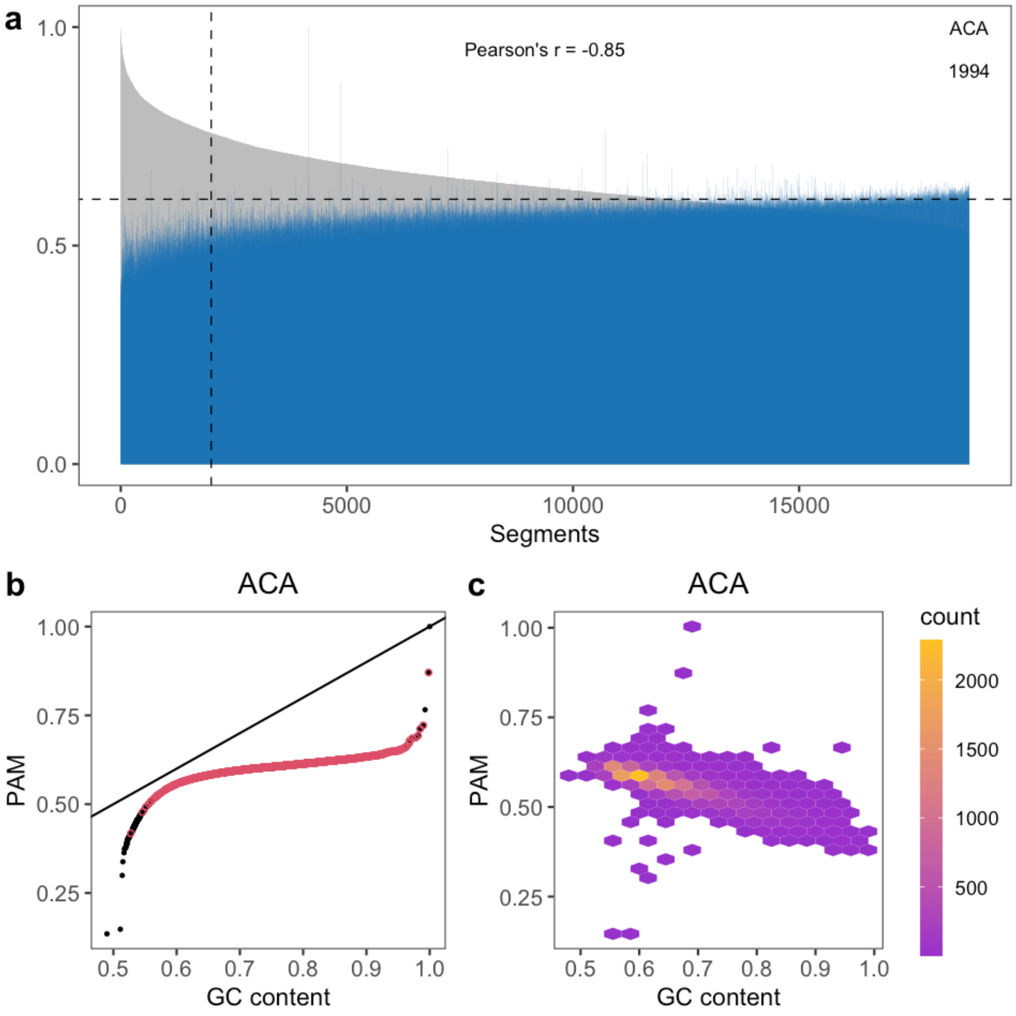

Supplementary Figure 23. Distribution of the ACA PAM enriched regions compared with that of the GC-rich regions. **a**. PAM (shown on the top-right) content of 18,763 segments (excluding 1,237 segments with all N bases), respectively. The genome segments are ordered by decreasing GC content (grey), and the corresponding PAM content is plotted in the same order (blue). The segments to the left of the vertical dashed line are the top 2,000 segments for GC content, and the segments above the horizontal dashed line are the top 2,000 segments for the ACA PAM. The number below the name of PAM (top-right) shows the number out of the top 2,000 PAM segments that are not in the top 2,000 GC-rich segments. The Pearson's r is the correlation between the GC content and the PAM content in all 18,763 segments. **b**. Quantile-quantile plot of the segments GC content v.s. PAM content distributions, where the red circles show the top 2,000 PAM segments, and the dashed line represents y = x. **c**. Hexbin plot of segments GC v.s. PAM content, where the color represents the count of points in each hexagon. GC and PAM content is normalized to the 0-1 range.

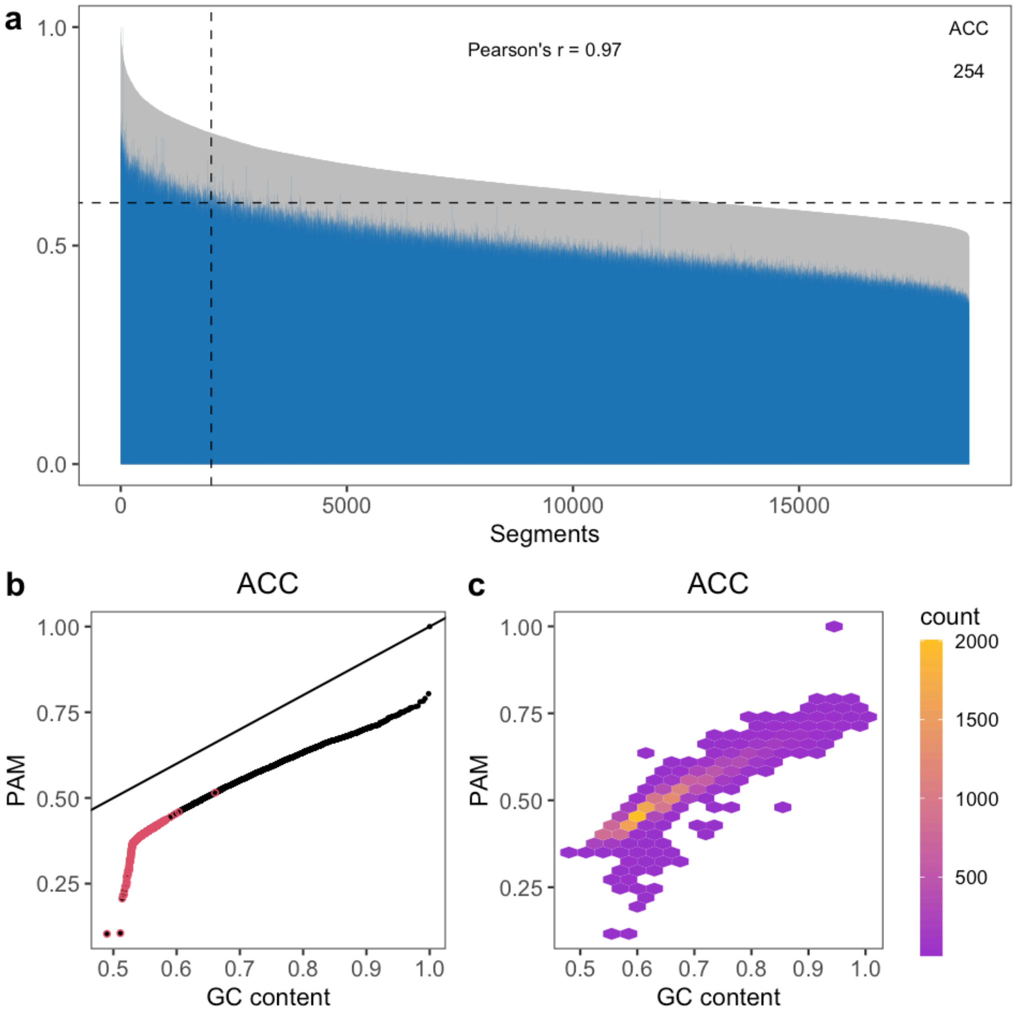

Supplementary Figure 24. Distribution of the ACC PAM enriched regions compared with that of the GC-rich regions. **a**. PAM (shown on the top-right) content of 18,763 segments (excluding 1,237 segments with all N bases), respectively. The genome segments are ordered by decreasing GC content (grey), and the corresponding PAM content is plotted in the same order (blue). The segments to the left of the vertical dashed line are the top 2,000 segments for GC content, and the segments above the horizontal dashed line are the top 2,000 segments for the ACC PAM. The number below the name of PAM (top-right) shows the number out of the top 2,000 PAM segments that are not in the top 2,000 GC-rich segments. The Pearson's r is the correlation between the GC content and the PAM content in all 18,763 segments. **b**. Quantile-quantile plot of the segments GC content v.s. PAM content distributions, where the red circles show the top 2,000 PAM segments, and the dashed line represents y = x. **c**. Hexbin plot of segments GC v.s. PAM content, where the color represents the count of points in each hexagon. GC and PAM content is normalized to the 0-1 range.

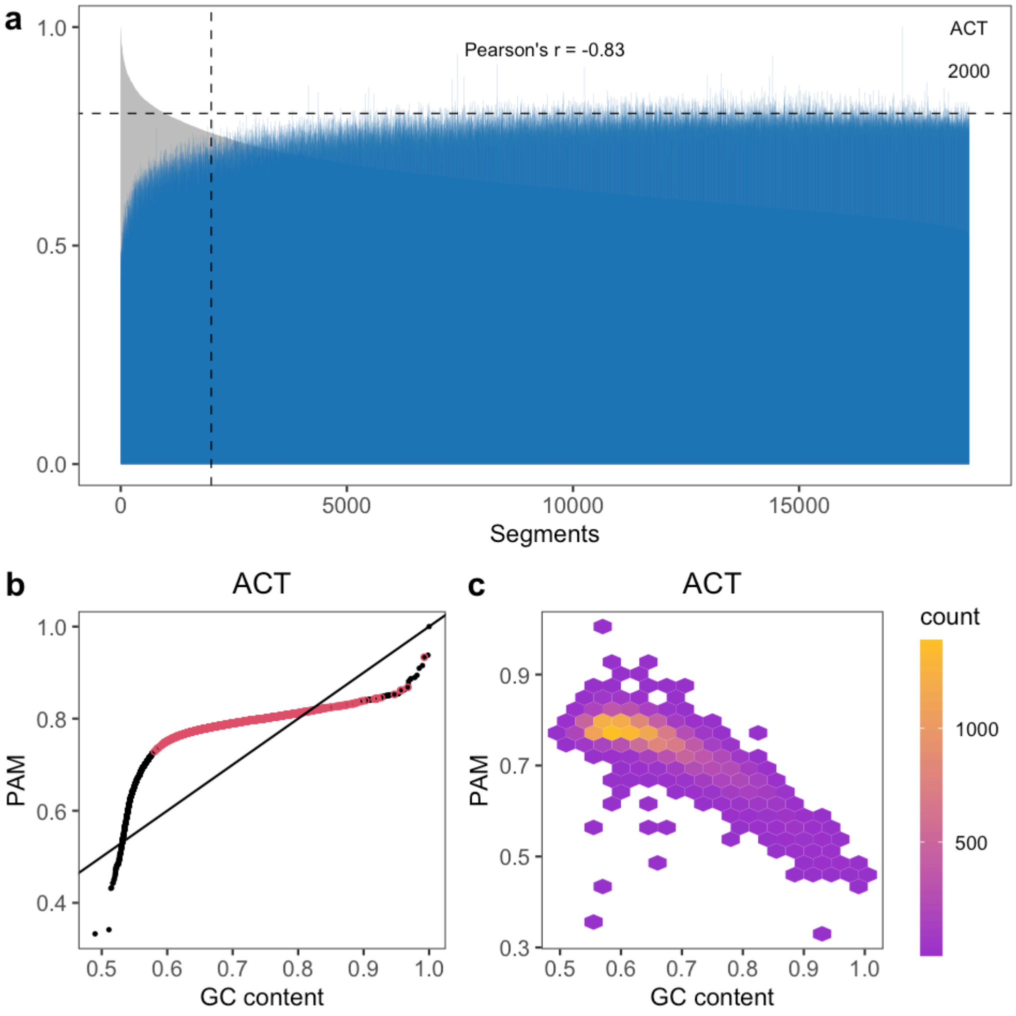

Supplementary Figure 25. Distribution of the ACT PAM enriched regions compared with that of the GC-rich regions. **a**. PAM (shown on the top-right) content of 18,763 segments (excluding 1,237 segments with all N bases), respectively. The genome segments are ordered by decreasing GC content (grey), and the corresponding PAM content is plotted in the same order (blue). The segments to the left of the vertical dashed line are the top 2,000 segments for GC content, and the segments above the horizontal dashed line are the top 2,000 segments for the ACT PAM. The number below the name of PAM (top-right) shows the number out of the top 2,000 PAM segments that are not in the top 2,000 GC-rich segments. The Pearson's r is the correlation between the GC content and the PAM content in all 18,763 segments. **b**. Quantile-quantile plot of the segments GC content v.s. PAM content distributions, where the red circles show the top 2,000 PAM segments, and the dashed line represents y = x. **c**. Hexbin plot of segments GC v.s. PAM content, where the color represents the count of points in each hexagon. GC and PAM content is normalized to the 0-1 range.

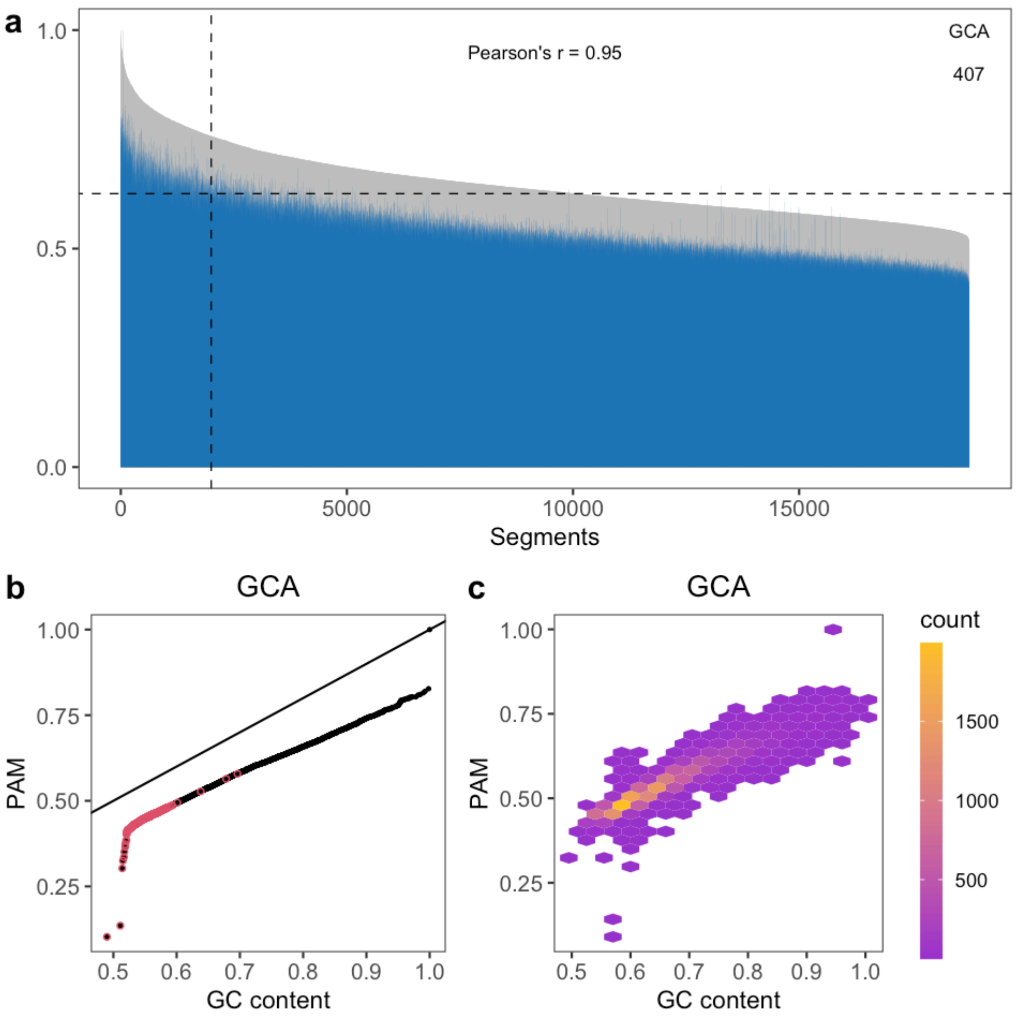

Supplementary Figure 26. Distribution of the GCA PAM enriched regions compared with that of the GC-rich regions. **a**. PAM (shown on the top-right) content of 18,763 segments (excluding 1,237 segments with all N bases), respectively. The genome segments are ordered by decreasing GC content (grey), and the corresponding PAM content is plotted in the same order (blue). The segments to the left of the vertical dashed line are the top 2,000 segments for GC content, and the segments above the horizontal dashed line are the top 2,000 segments for the GCA PAM. The number below the name of PAM (top-right) shows the number out of the top 2,000 PAM segments that are not in the top 2,000 GC-rich segments. The Pearson's r is the correlation between the GC content and the PAM content in all 18,763 segments. **b**. Quantile-quantile plot of the segments GC content v.s. PAM content distributions, where the red circles show the top 2,000 PAM segments, and the dashed line represents y = x. **c**. Hexbin plot of segments GC v.s. PAM content, where the color represents the count of points in each hexagon. GC and PAM content is normalized to the 0-1 range.

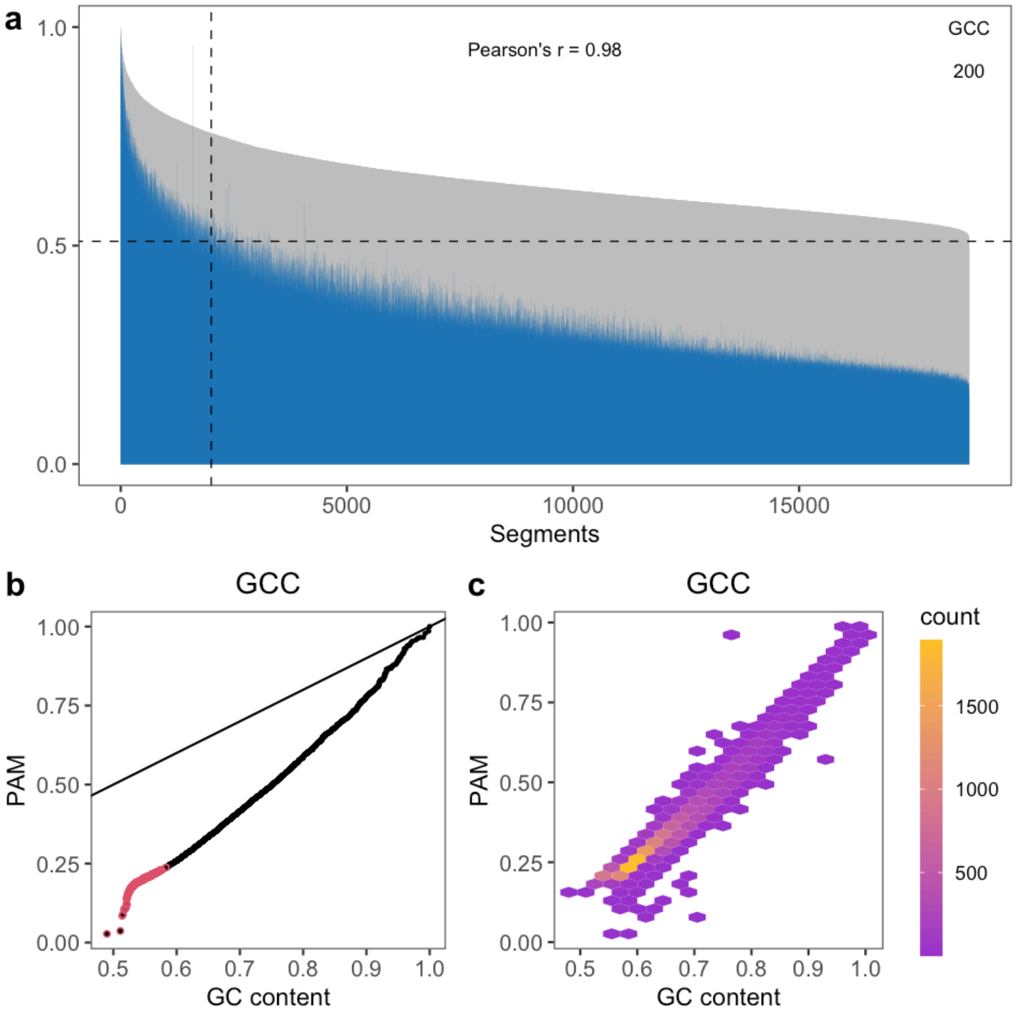

Supplementary Figure 27. Distribution of the GCC PAM enriched regions compared with that of the GC-rich regions. **a**. PAM (shown on the top-right) content of 18,763 segments (excluding 1,237 segments with all N bases), respectively. The genome segments are ordered by decreasing GC content (grey), and the corresponding PAM content is plotted in the same order (blue). The segments to the left of the vertical dashed line are the top 2,000 segments for GC content, and the segments above the horizontal dashed line are the top 2,000 segments for the GCC PAM. The number below the name of PAM (top-right) shows the number out of the top 2,000 PAM segments that are not in the top 2,000 GC-rich segments. The Pearson's r is the correlation between the GC content and the PAM content in all 18,763 segments. **b**. Quantile-quantile plot of the segments GC content v.s. PAM content distributions, where the red circles show the top 2,000 PAM segments, and the dashed line represents y = x. **c**. Hexbin plot of segments GC v.s. PAM content, where the color represents the count of points in each hexagon. GC and PAM content is normalized to the 0-1 range.

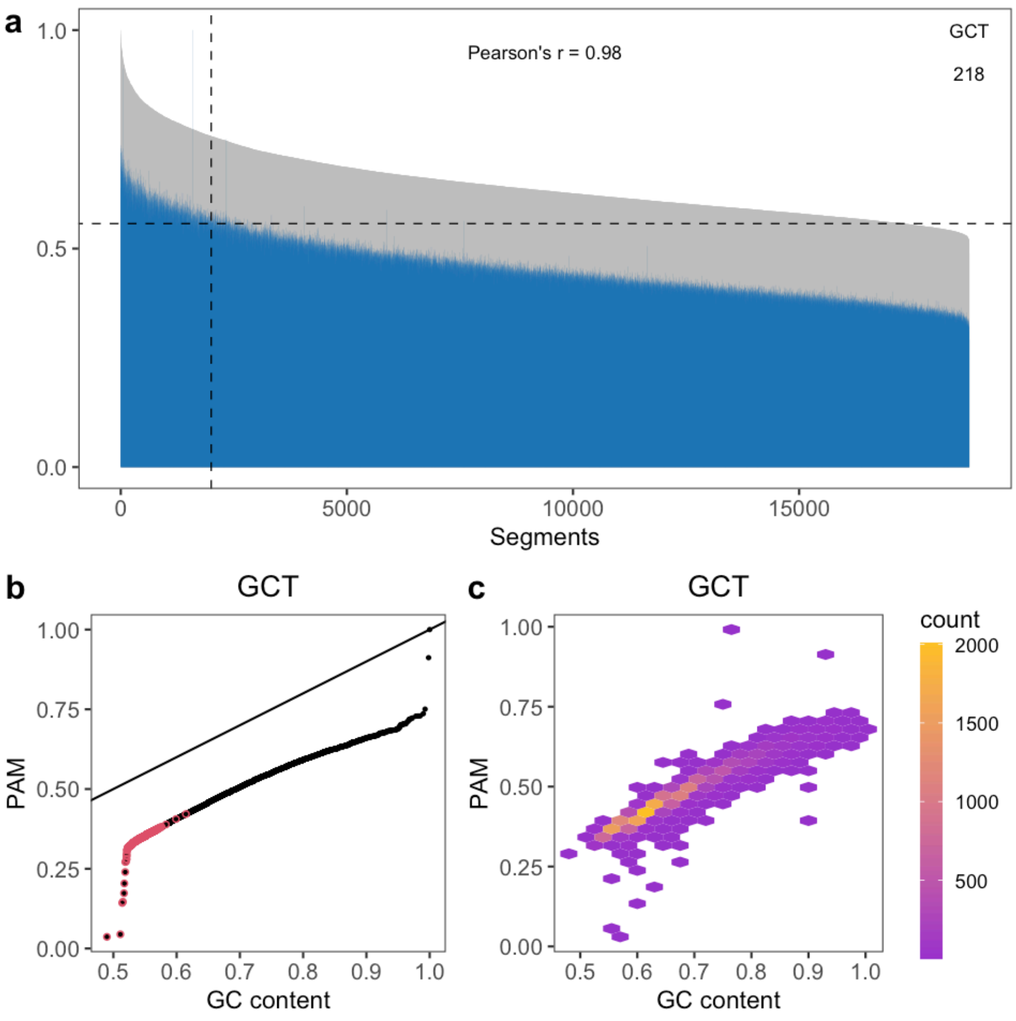

Supplementary Figure 28. Distribution of the GCT PAM enriched regions compared with that of the GC-rich regions. **a**. PAM (shown on the top-right) content of 18,763 segments (excluding 1,237 segments with all N bases), respectively. The genome segments are ordered by decreasing GC content (grey), and the corresponding PAM content is plotted in the same order (blue). The segments to the left of the vertical dashed line are the top 2,000 segments for GC content, and the segments above the horizontal dashed line are the top 2,000 segments for the GCT PAM. The number below the name of PAM (top-right) shows the number out of the top 2,000 PAM segments that are not in the top 2,000 GC-rich segments. The Pearson's r is the correlation between the GC content and the PAM content in all 18,763 segments. **b**. Quantile-quantile plot of the segments GC content v.s. PAM content distributions, where the red circles show the top 2,000 PAM segments, and the dashed line represents y = x. **c**. Hexbin plot of segments GC v.s. PAM content, where the color represents the count of points in each hexagon. GC and PAM content is normalized to the 0-1 range.

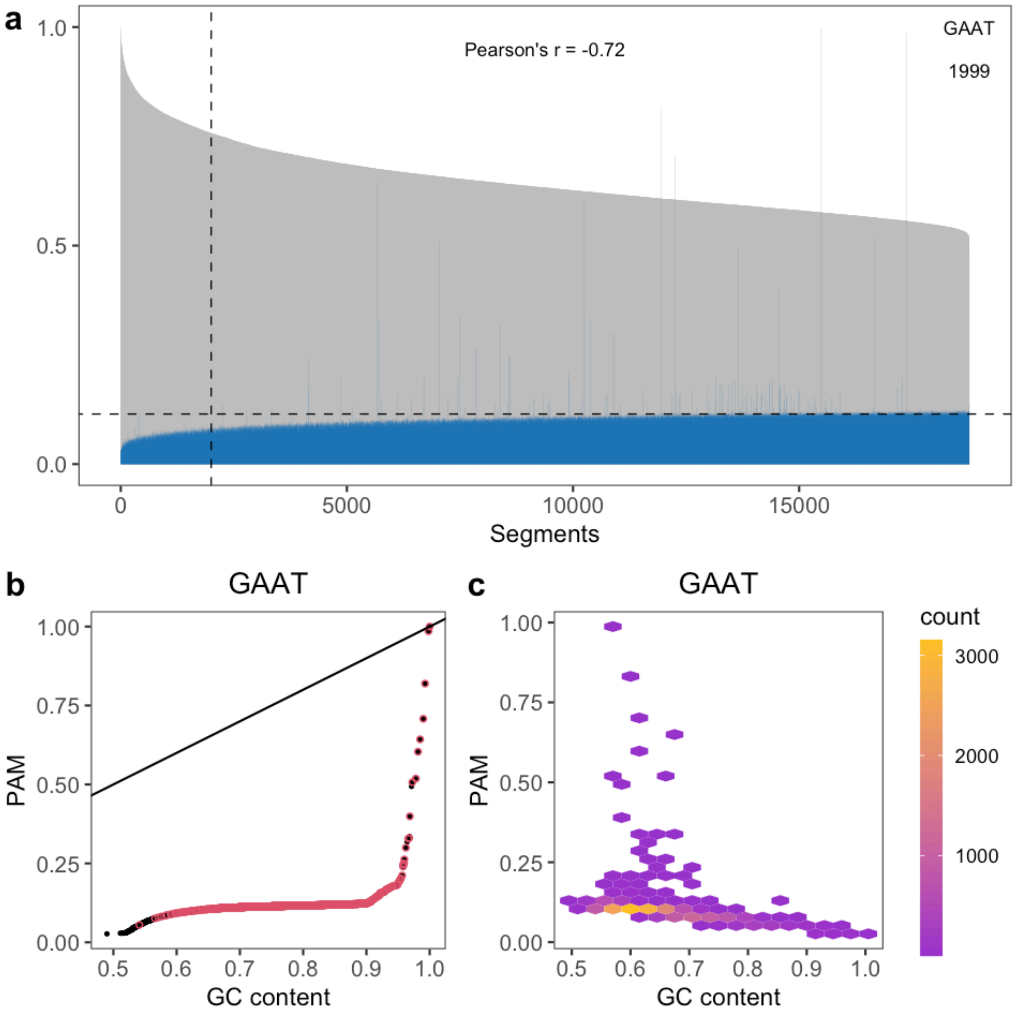

Supplementary Figure 29. Distribution of the GAAT PAM enriched regions compared with that of the GC-rich regions. **a**. PAM (shown on the top-right) content of 18,763 segments (excluding 1,237 segments with all N bases), respectively. The genome segments are ordered by decreasing GC content (grey), and the corresponding PAM content is plotted in the same order (blue). The segments to the left of the vertical dashed line are the top 2,000 segments for GC content, and the segments above the horizontal dashed line are the top 2,000 segments for the GAAT PAM. The number below the name of PAM (top-right) shows the number out of the top 2,000 PAM segments that are not in the top 2,000 GC-rich segments. The Pearson's r is the correlation between the GC content and the PAM content in all 18,763 segments. **b**. Quantile-quantile plot of the segments GC content v.s. PAM content distributions, where the red circles show the top 2,000 PAM segments, and the dashed line represents y = x. **c**. Hexbin plot of segments GC v.s. PAM content, where the color represents the count of points in each hexagon. GC and PAM content is normalized to the 0-1 range.

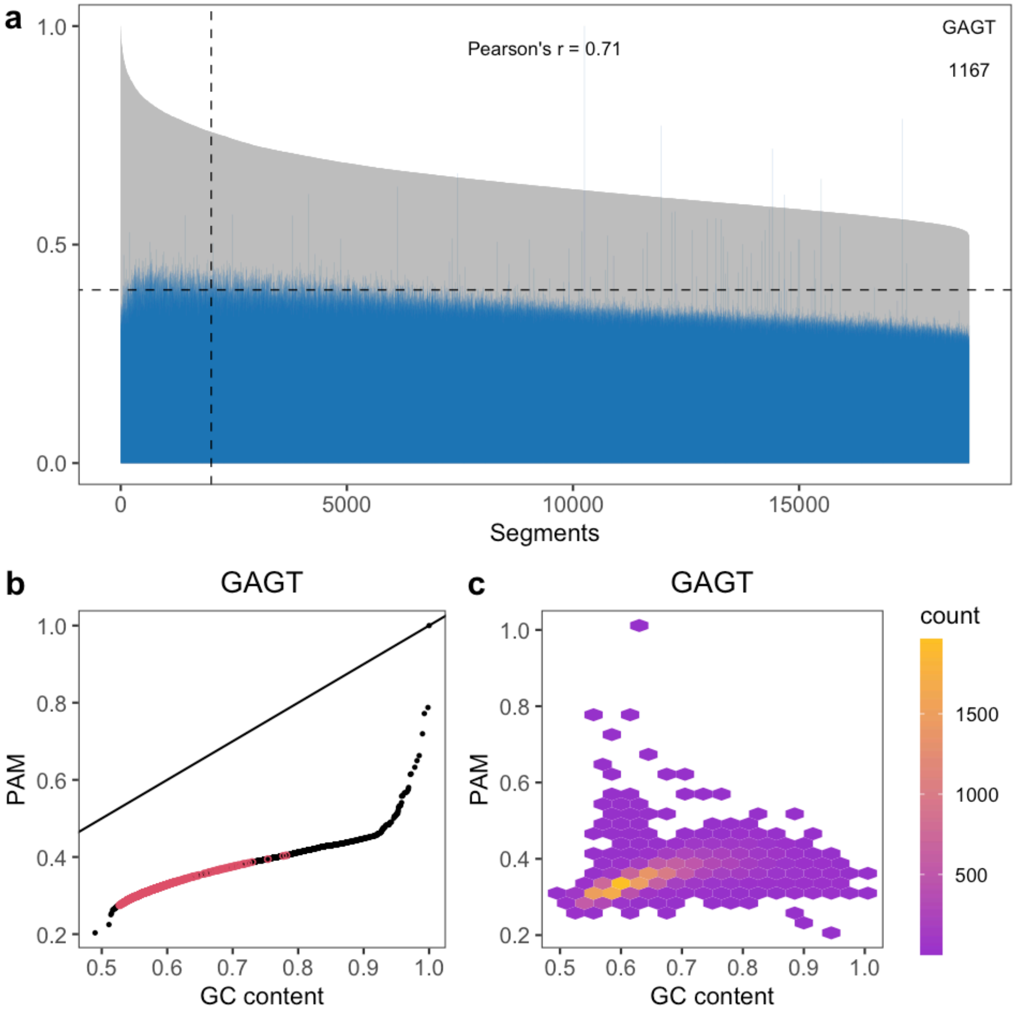

Supplementary Figure 30. Distribution of the GAGT PAM enriched regions compared with that of the GC-rich regions. **a**. PAM (shown on the top-right) content of 18,763 segments (excluding 1,237 segments with all N bases), respectively. The genome segments are ordered by decreasing GC content (grey), and the corresponding PAM content is plotted in the same order (blue). The segments to the left of the vertical dashed line are the top 2,000 segments for GC content, and the segments above the horizontal dashed line are the top 2,000 segments for the GAGT PAM. The number below the name of PAM (top-right) shows the number out of the top 2,000 PAM segments that are not in the top 2,000 GC-rich segments. The Pearson's r is the correlation between the GC content and the PAM content in all 18,763 segments. **b**. Quantile-quantile plot of the segments GC content v.s. PAM content distributions, where the red circles show the top 2,000 PAM segments, and the dashed line represents y = x. **c**. Hexbin plot of segments GC v.s. PAM content, where the color represents the count of points in each hexagon. GC and PAM content is normalized to the 0-1 range.

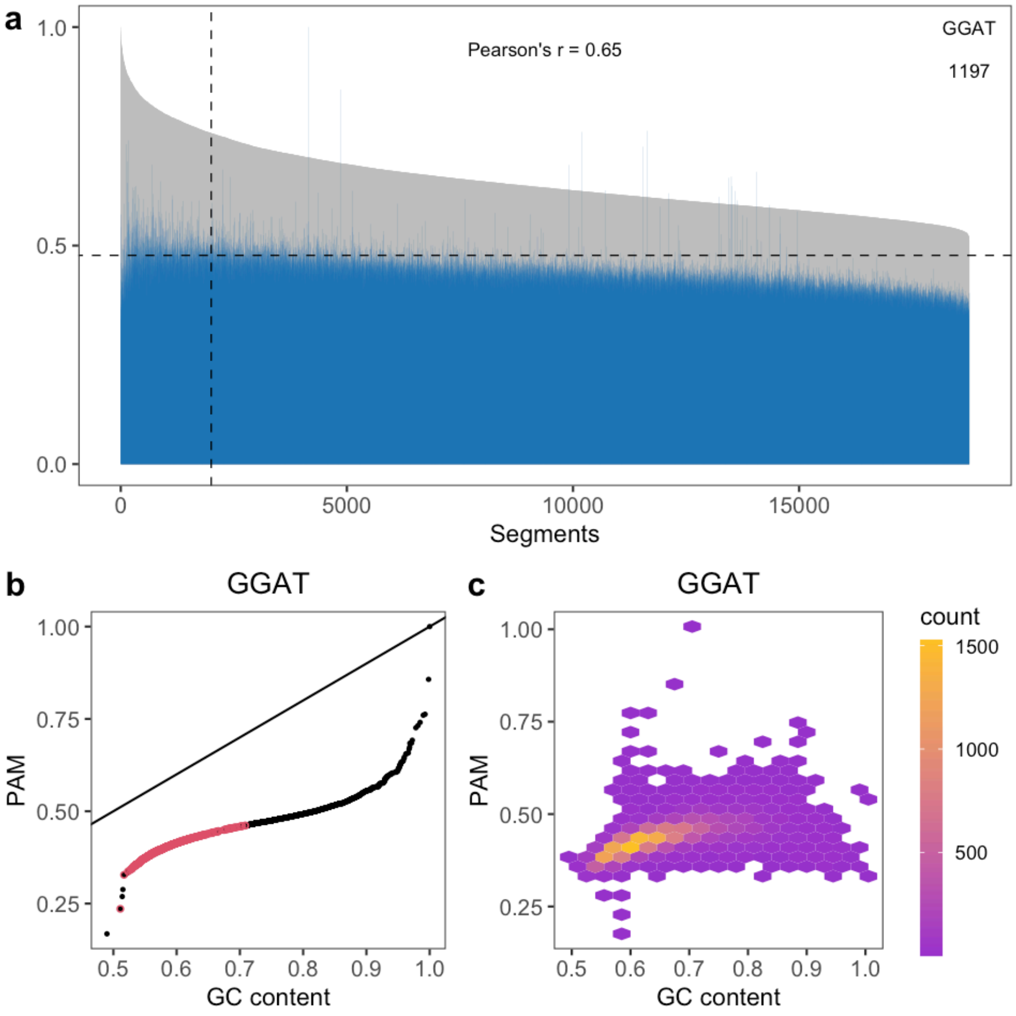

Supplementary Figure 31. Distribution of the GGAT PAM enriched regions compared with that of the GC-rich regions. **a**. PAM (shown on the top-right) content of 18,763 segments (excluding 1,237 segments with all N bases), respectively. The genome segments are ordered by decreasing GC content (grey), and the corresponding PAM content is plotted in the same order (blue). The segments to the left of the vertical dashed line are the top 2,000 segments for GC content, and the segments above the horizontal dashed line are the top 2,000 segments for the GGAT PAM. The number below the name of PAM (top-right) shows the number out of the top 2,000 PAM segments that are not in the top 2,000 GC-rich segments. The Pearson's r is the correlation between the GC content and the PAM content in all 18,763 segments. **b**. Quantile-quantile plot of the segments GC content v.s. PAM content distributions, where the red circles show the top 2,000 PAM segments, and the dashed line represents y = x. **c**. Hexbin plot of segments GC v.s. PAM content, where the color represents the count of points in each hexagon. GC and PAM content is normalized to the 0-1 range.

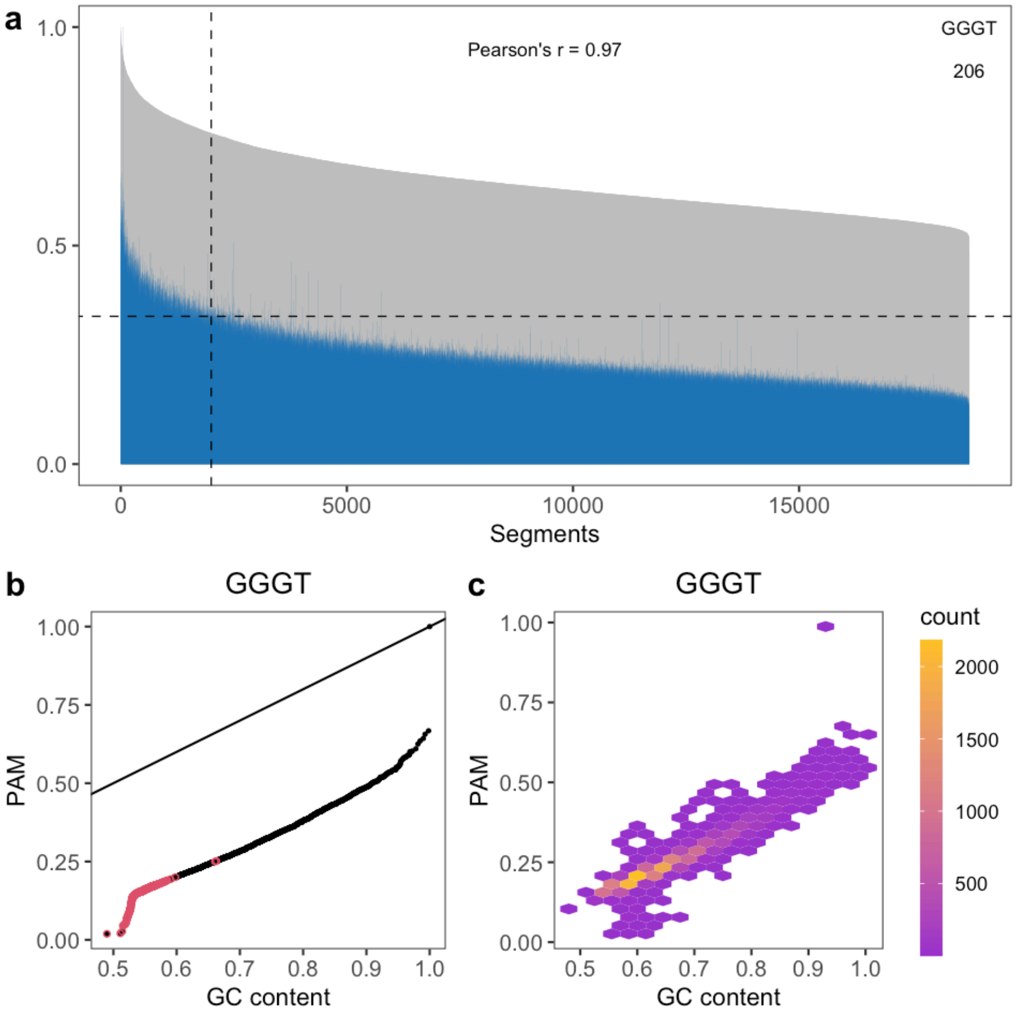

Supplementary Figure 32. Distribution of the GGGT PAM enriched regions compared with that of the GC-rich regions. **a**. PAM (shown on the top-right) content of 18,763 segments (excluding 1,237 segments with all N bases), respectively. The genome segments are ordered by decreasing GC content (grey), and the corresponding PAM content is plotted in the same order (blue). The segments to the left of the vertical dashed line are the top 2,000 segments for GC content, and the segments above the horizontal dashed line are the top 2,000 segments for the GGGT PAM. The number below the name of PAM (top-right) shows the number out of the top 2,000 PAM segments that are not in the top 2,000 GC-rich segments. The Pearson's r is the correlation between the GC content and the PAM content in all 18,763 segments. **b**. Quantile-quantile plot of the segments GC content v.s. PAM content distributions, where the red circles show the top 2,000 PAM segments, and the dashed line represents y = x. **c**. Hexbin plot of segments GC v.s. PAM content, where the color represents the count of points in each hexagon. GC and PAM content is normalized to the 0-1 range.

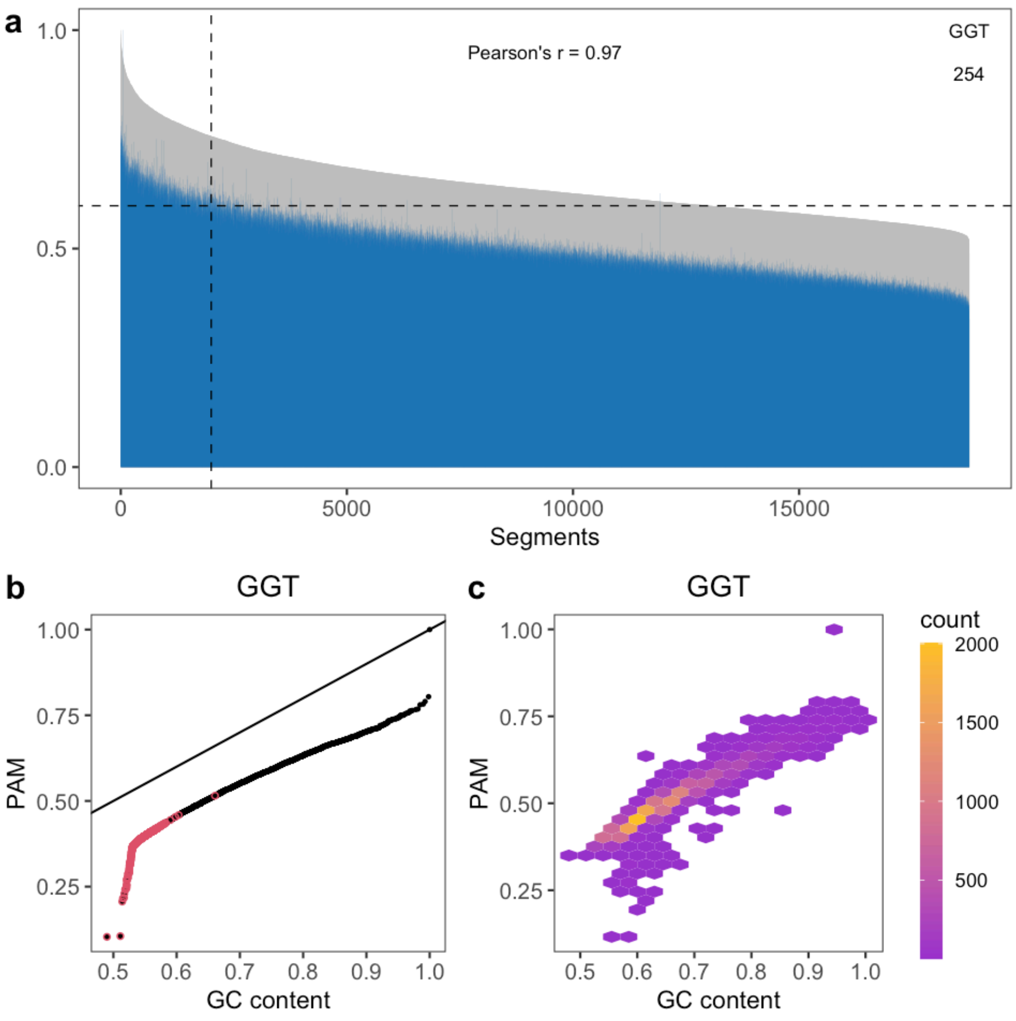

Supplementary Figure 33. Distribution of the GGT PAM enriched regions compared with that of the GC-rich regions. **a**. PAM (shown on the top-right) content of 18,763 segments (excluding 1,237 segments with all N bases), respectively. The genome segments are ordered by decreasing GC content (grey), and the corresponding PAM content is plotted in the same order (blue). The segments to the left of the vertical dashed line are the top 2,000 segments for GC content, and the segments above the horizontal dashed line are the top 2,000 segments for the GGT PAM. The number below the name of PAM (top-right) shows the number out of the top 2,000 PAM segments that are not in the top 2,000 GC-rich segments. The Pearson's r is the correlation between the GC content and the PAM content in all 18,763 segments. **b**. Quantile-quantile plot of the segments GC content v.s. PAM content distributions, where the red circles show the top 2,000 PAM segments, and the dashed line represents y = x. **c**. Hexbin plot of segments GC v.s. PAM content, where the color represents the count of points in each hexagon. GC and PAM content is normalized to the 0-1 range.

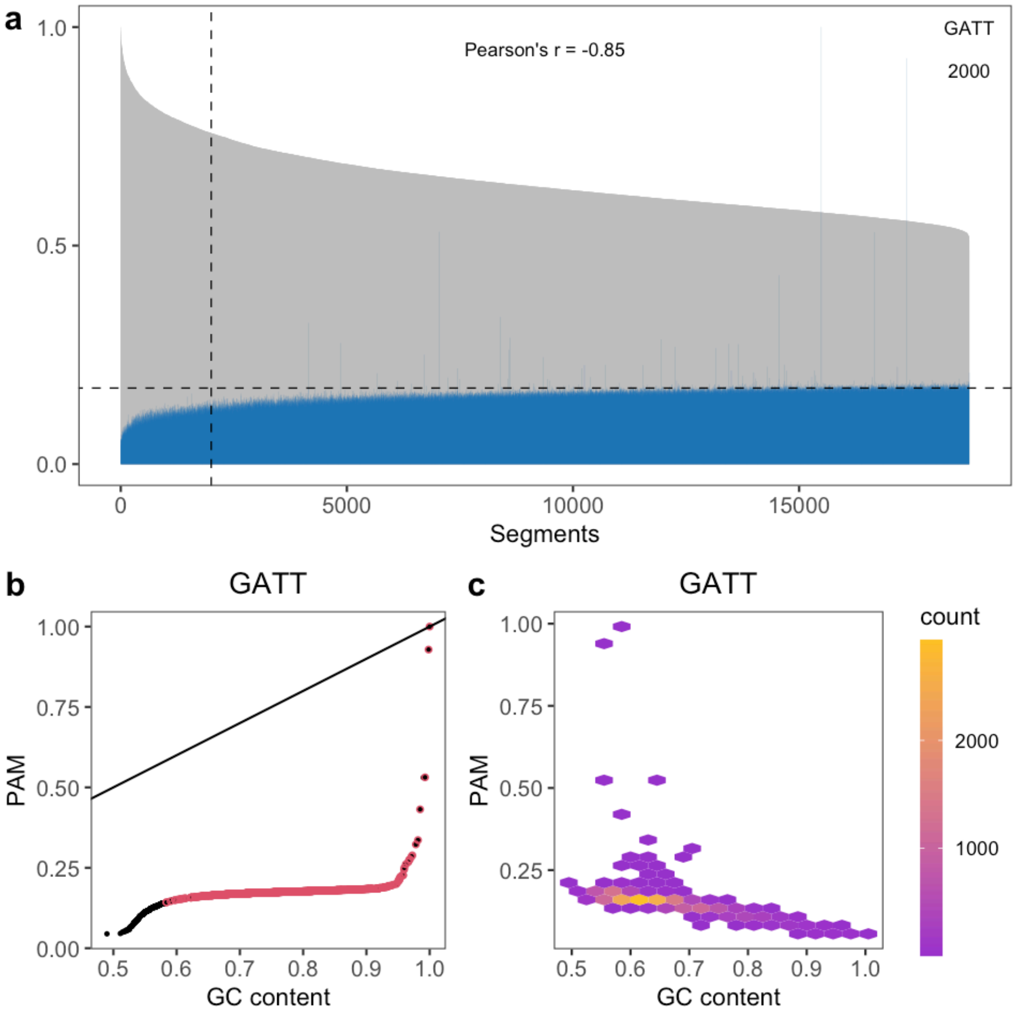

Supplementary Figure 34. Distribution of the GATT PAM enriched regions compared with that of the GC-rich regions. **a**. PAM (shown on the top-right) content of 18,763 segments (excluding 1,237 segments with all N bases), respectively. The genome segments are ordered by decreasing GC content (grey), and the corresponding PAM content is plotted in the same order (blue). The segments to the left of the vertical dashed line are the top 2,000 segments for GC content, and the segments above the horizontal dashed line are the top 2,000 segments for the GATT PAM. The number below the name of PAM (top-right) shows the number out of the top 2,000 PAM segments that are not in the top 2,000 GC-rich segments. The Pearson's r is the correlation between the GC content and the PAM content in all 18,763 segments. **b**. Quantile-quantile plot of the segments GC content v.s. PAM content distributions, where the red circles show the top 2,000 PAM segments, and the dashed line represents y = x. **c**. Hexbin plot of segments GC v.s. PAM content, where the color represents the count of points in each hexagon. GC and PAM content is normalized to the 0-1 range.

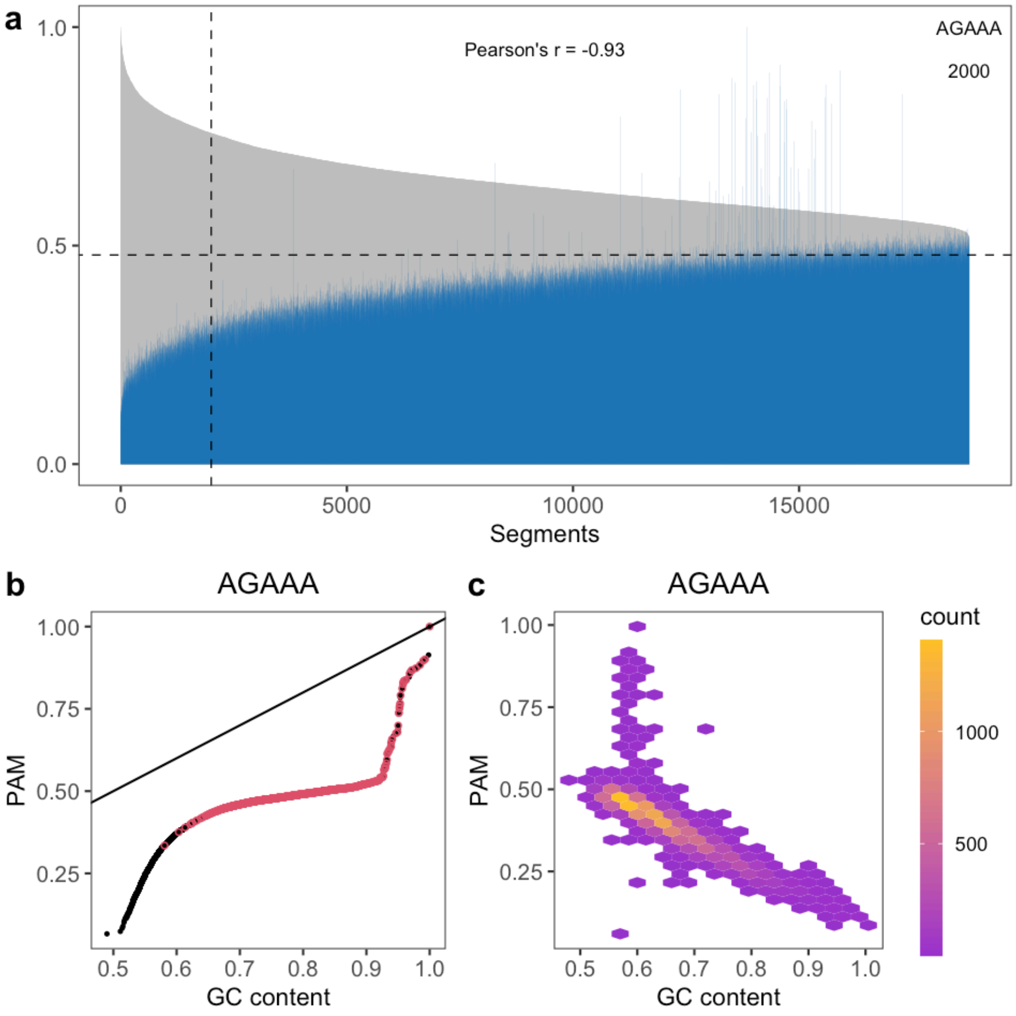

Supplementary Figure 35. Distribution of the AGAAA PAM enriched regions compared with that of the GC-rich regions. **a**. PAM (shown on the top-right) content of 18,763 segments (excluding 1,237 segments with all N bases), respectively. The genome segments are ordered by decreasing GC content (grey), and the corresponding PAM content is plotted in the same order (blue). The segments to the left of the vertical dashed line are the top 2,000 segments for GC content, and the segments above the horizontal dashed line are the top 2,000 segments for the AGAAA PAM. The number below the name of PAM (top-right) shows the number out of the top 2,000 PAM segments that are not in the top 2,000 GC-rich segments. The Pearson's r is the correlation between the GC content and the PAM content in all 18,763 segments. **b**. Quantile-quantile plot of the segments GC content v.s. PAM content distributions, where the red circles show the top 2,000 PAM segments, and the dashed line represents y = x. **c**. Hexbin plot of segments GC v.s. PAM content, where the color represents the count of points in each hexagon. GC and PAM content is normalized to the 0-1 range.

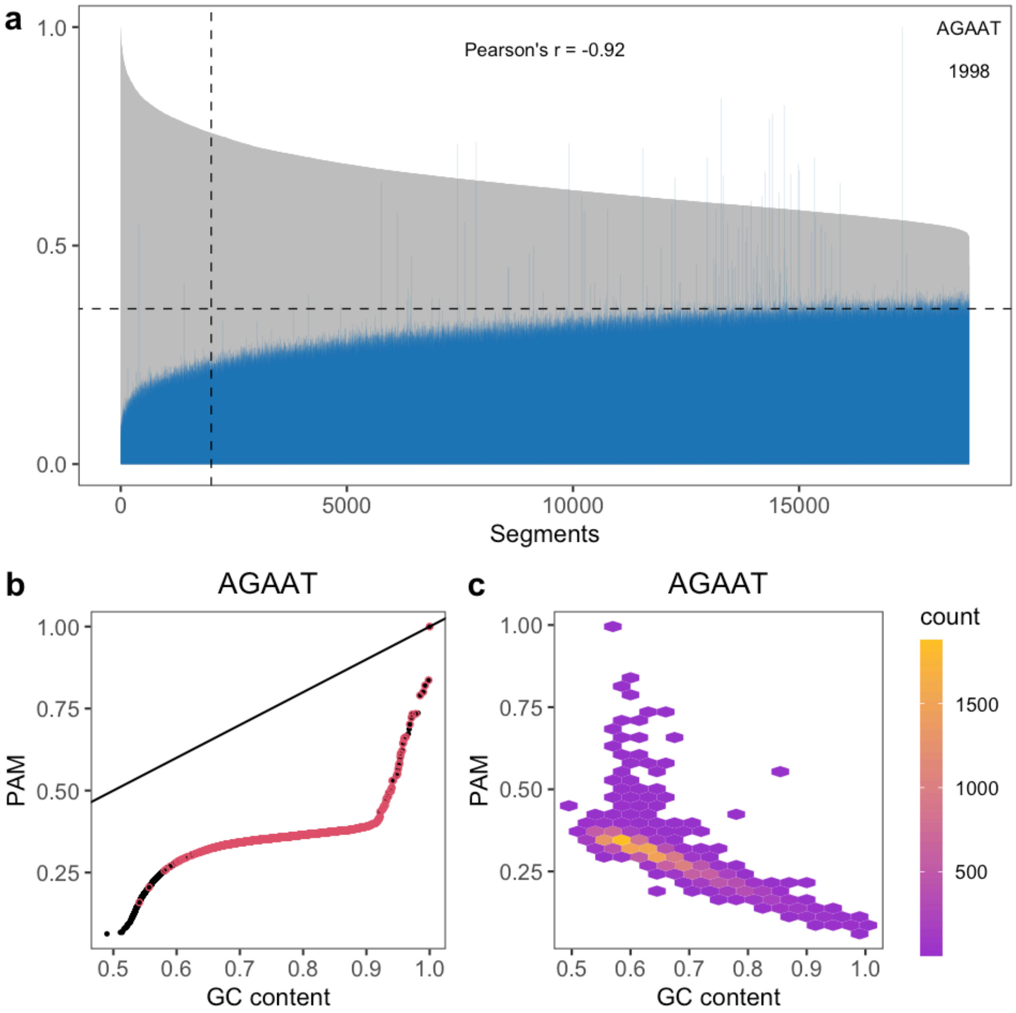

Supplementary Figure 36. Distribution of the AGAAT PAM enriched regions compared with that of the GC-rich regions. **a**. PAM (shown on the top-right) content of 18,763 segments (excluding 1,237 segments with all N bases), respectively. The genome segments are ordered by decreasing GC content (grey), and the corresponding PAM content is plotted in the same order (blue). The segments to the left of the vertical dashed line are the top 2,000 segments for GC content, and the segments above the horizontal dashed line are the top 2,000 segments for the AGAAT PAM. The number below the name of PAM (top-right) shows the number out of the top 2,000 PAM segments that are not in the top 2,000 GC-rich segments. The Pearson's r is the correlation between the GC content and the PAM content in all 18,763 segments. **b**. Quantile-quantile plot of the segments GC content v.s. PAM content distributions, where the red circles show the top 2,000 PAM segments, and the dashed line represents y = x. **c**. Hexbin plot of segments GC v.s. PAM content, where the color represents the count of points in each hexagon. GC and PAM content is normalized to the 0-1 range.

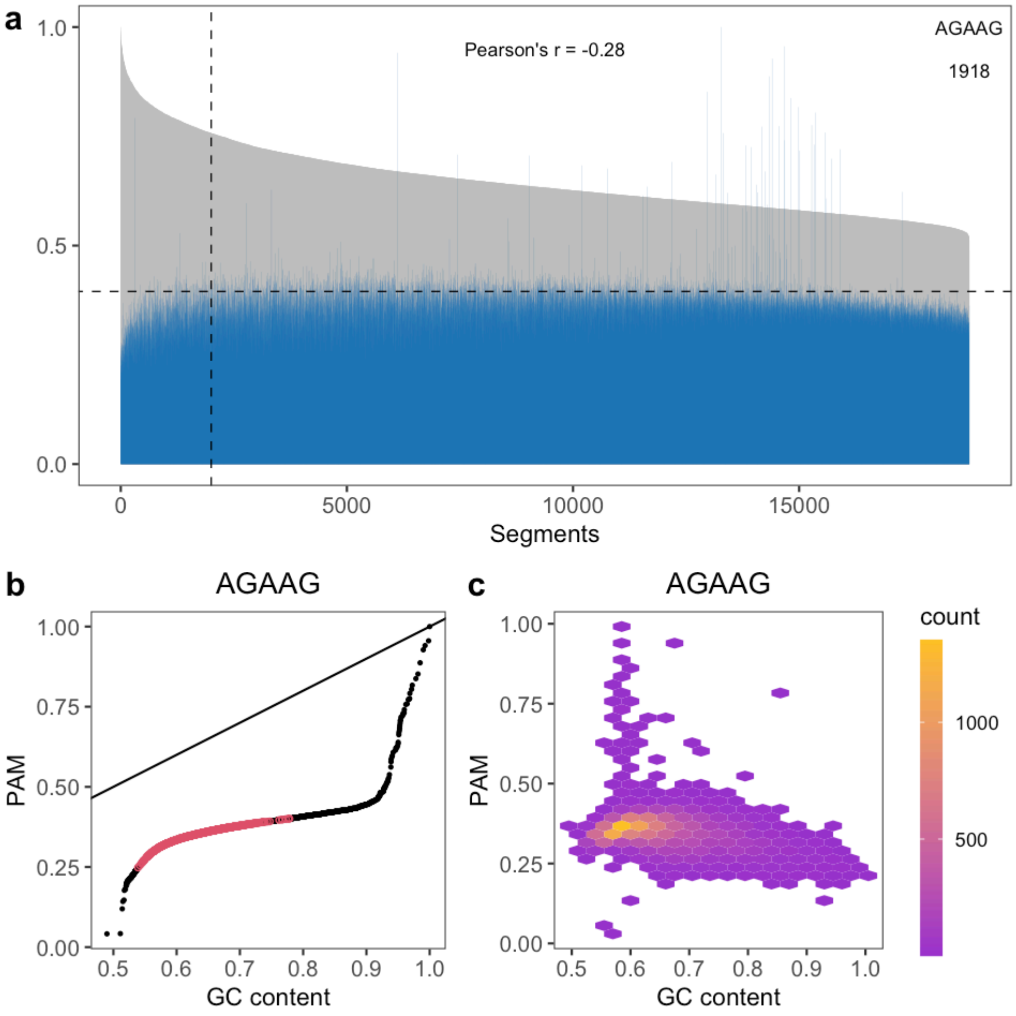

Supplementary Figure 37. Distribution of the AGAAG PAM enriched regions compared with that of the GC-rich regions. **a**. PAM (shown on the top-right) content of 18,763 segments (excluding 1,237 segments with all N bases), respectively. The genome segments are ordered by decreasing GC content (grey), and the corresponding PAM content is plotted in the same order (blue). The segments to the left of the vertical dashed line are the top 2,000 segments for GC content, and the segments above the horizontal dashed line are the top 2,000 segments for the AGAAG PAM. The number below the name of PAM (top-right) shows the number out of the top 2,000 PAM segments that are not in the top 2,000 GC-rich segments. The Pearson's r is the correlation between the GC content and the PAM content in all 18,763 segments. **b**. Quantile-quantile plot of the segments GC content v.s. PAM content distributions, where the red circles show the top 2,000 PAM segments, and the dashed line represents y = x. **c**. Hexbin plot of segments GC v.s. PAM content, where the color represents the count of points in each hexagon. GC and PAM content is normalized to the 0-1 range.

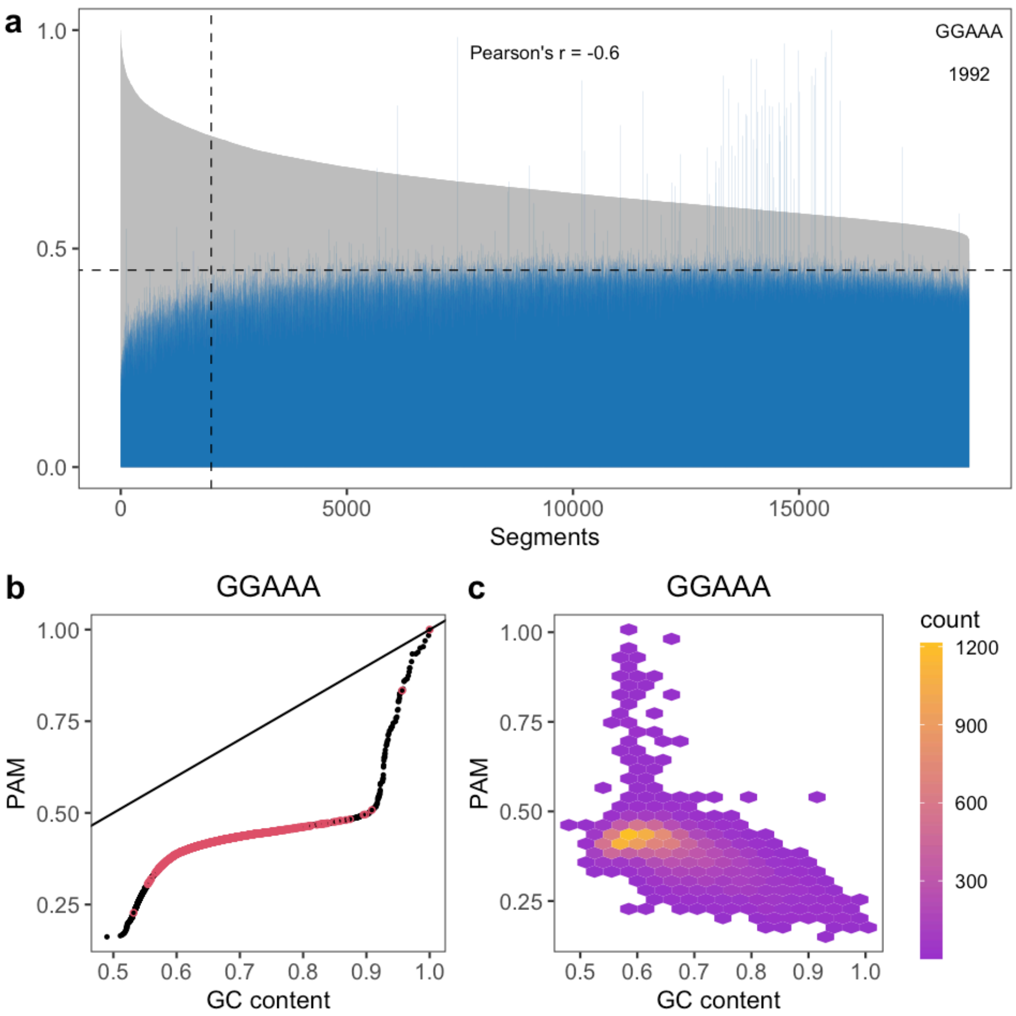

Supplementary Figure 38. Distribution of the GGAAA PAM enriched regions compared with that of the GC-rich regions. **a**. PAM (shown on the top-right) content of 18,763 segments (excluding 1,237 segments with all N bases), respectively. The genome segments are ordered by decreasing GC content (grey), and the corresponding PAM content is plotted in the same order (blue). The segments to the left of the vertical dashed line are the top 2,000 segments for GC content, and the segments above the horizontal dashed line are the top 2,000 segments for the GGAAA PAM. The number below the name of PAM (top-right) shows the number out of the top 2,000 PAM segments that are not in the top 2,000 GC-rich segments. The Pearson's r is the correlation between the GC content and the PAM content in all 18,763 segments. **b**. Quantile-quantile plot of the segments GC content v.s. PAM content distributions, where the red circles show the top 2,000 PAM segments, and the dashed line represents y = x. **c**. Hexbin plot of segments GC v.s. PAM content, where the color represents the count of points in each hexagon. GC and PAM content is normalized to the 0-1 range.

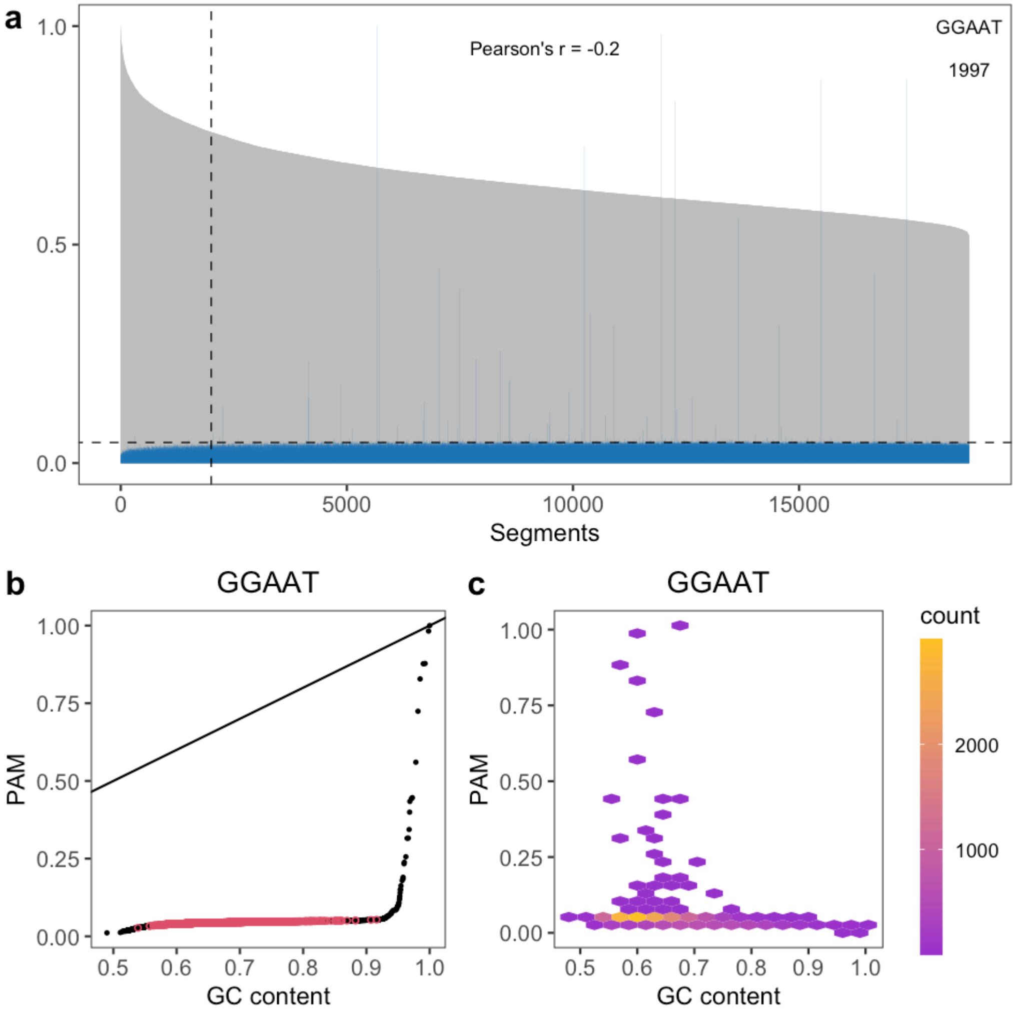

Supplementary Figure 39. Distribution of the GGAAT PAM enriched regions compared with that of the GC-rich regions. **a**. PAM (shown on the top-right) content of 18,763 segments (excluding 1,237 segments with all N bases), respectively. The genome segments are ordered by decreasing GC content (grey), and the corresponding PAM content is plotted in the same order (blue). The segments to the left of the vertical dashed line are the top 2,000 segments for GC content, and the segments above the horizontal dashed line are the top 2,000 segments for the GGAAT PAM. The number below the name of PAM (top-right) shows the number out of the top 2,000 PAM segments that are not in the top 2,000 GC-rich segments. The Pearson's r is the correlation between the GC content and the PAM content in all 18,763 segments. **b**. Quantile-quantile plot of the segments GC content v.s. PAM content distributions, where the red circles show the top 2,000 PAM segments, and the dashed line represents y = x. **c**. Hexbin plot of segments GC v.s. PAM content, where the color represents the count of points in each hexagon. GC and PAM content is normalized to the 0-1 range.

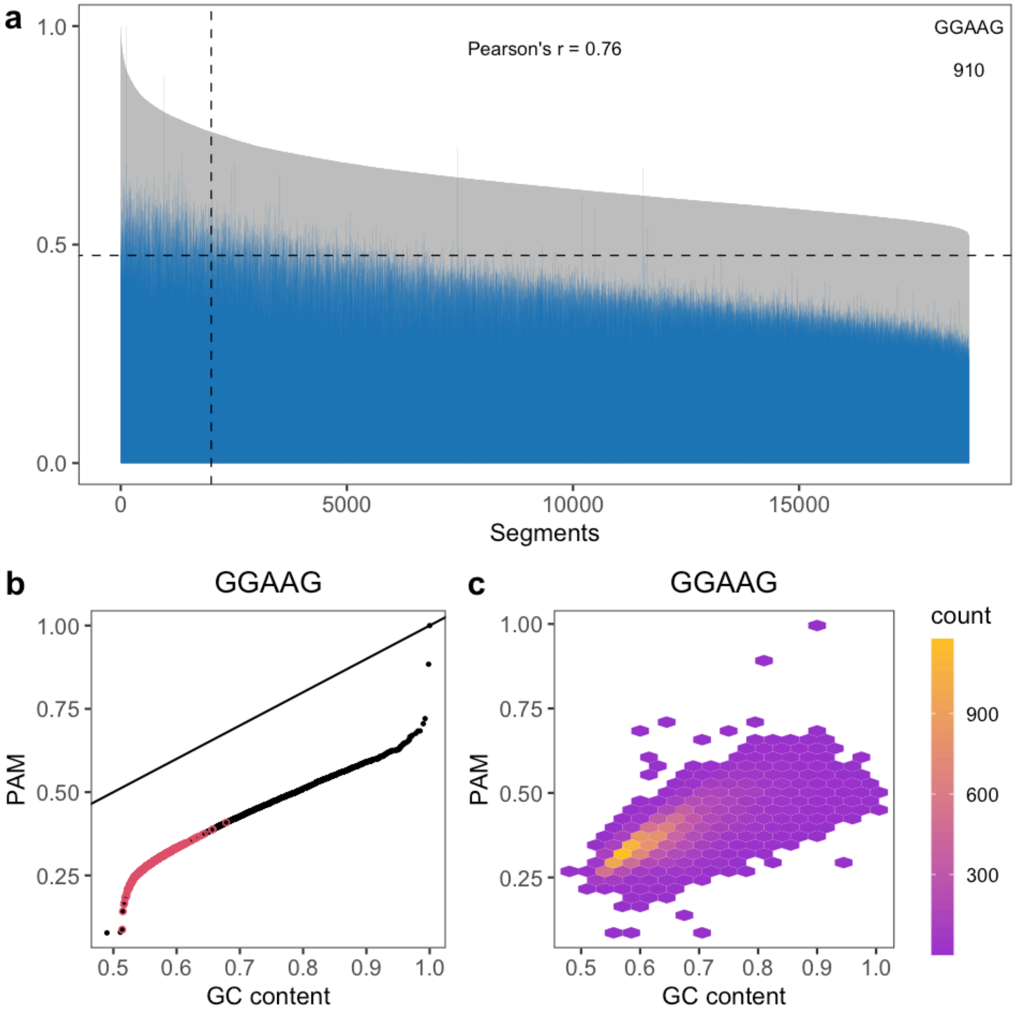

Supplementary Figure 40. Distribution of the GGAAG PAM enriched regions compared with that of the GC-rich regions. **a**. PAM (shown on the top-right) content of 18,763 segments (excluding 1,237 segments with all N bases), respectively. The genome segments are ordered by decreasing GC content (grey), and the corresponding PAM content is plotted in the same order (blue). The segments to the left of the vertical dashed line are the top 2,000 segments for GC content, and the segments above the horizontal dashed line are the top 2,000 segments for the GGAAG PAM. The number below the name of PAM (top-right) shows the number out of the top 2,000 PAM segments that are not in the top 2,000 GC-rich segments. The Pearson's r is the correlation between the GC content and the PAM content in all 18,763 segments. **b**. Quantile-quantile plot of the segments GC content v.s. PAM content distributions, where the red circles show the top 2,000 PAM segments, and the dashed line represents y = x. **c**. Hexbin plot of segments GC v.s. PAM content, where the color represents the count of points in each hexagon. GC and PAM content is normalized to the 0-1 range.

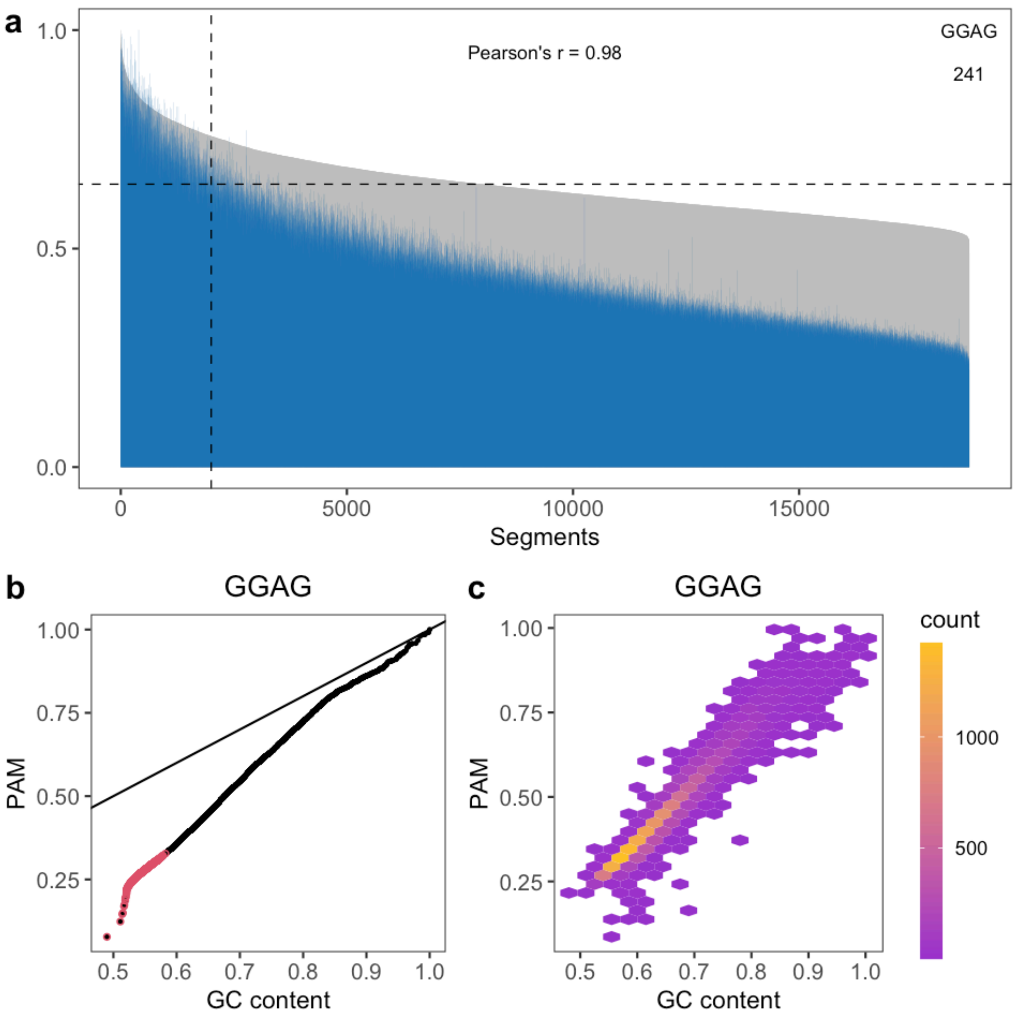

Supplementary Figure 41. Distribution of the GGAG PAM enriched regions compared with that of the GC-rich regions. **a**. PAM (shown on the top-right) content of 18,763 segments (excluding 1,237 segments with all N bases), respectively. The genome segments are ordered by decreasing GC content (grey), and the corresponding PAM content is plotted in the same order (blue). The segments to the left of the vertical dashed line are the top 2,000 segments for GC content, and the segments above the horizontal dashed line are the top 2,000 segments for the GGAG PAM. The number below the name of PAM (top-right) shows the number out of the top 2,000 PAM segments that are not in the top 2,000 GC-rich segments. The Pearson's r is the correlation between the GC content and the PAM content in all 18,763 segments. **b**. Quantile-quantile plot of the segments GC content v.s. PAM content distributions, where the red circles show the top 2,000 PAM segments, and the dashed line represents y = x. **c**. Hexbin plot of segments GC v.s. PAM content, where the color represents the count of points in each hexagon. GC and PAM content is normalized to the 0-1 range.

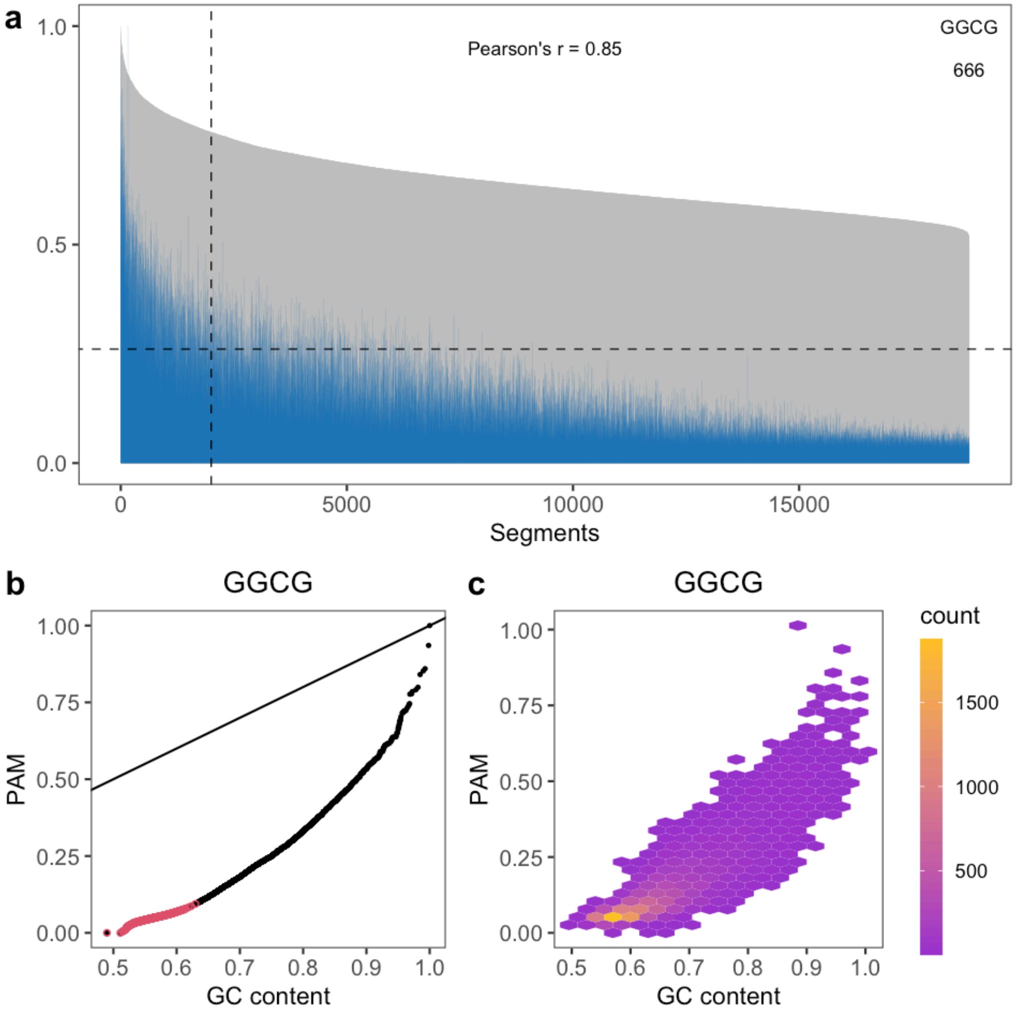

Supplementary Figure 42. Distribution of the GGCG PAM enriched regions compared with that of the GC-rich regions. **a**. PAM (shown on the top-right) content of 18,763 segments (excluding 1,237 segments with all N bases), respectively. The genome segments are ordered by decreasing GC content (grey), and the corresponding PAM content is plotted in the same order (blue). The segments to the left of the vertical dashed line are the top 2,000 segments for GC content, and the segments above the horizontal dashed line are the top 2,000 segments for the GGCG PAM. The number below the name of PAM (top-right) shows the number out of the top 2,000 PAM segments that are not in the top 2,000 GC-rich segments. The Pearson's r is the correlation between the GC content and the PAM content in all 18,763 segments. **b**. Quantile-quantile plot of the segments GC content v.s. PAM content distributions, where the red circles show the top 2,000 PAM segments, and the dashed line represents y = x. **c**. Hexbin plot of segments GC v.s. PAM content, where the color represents the count of points in each hexagon. GC and PAM content is normalized to the 0-1 range.

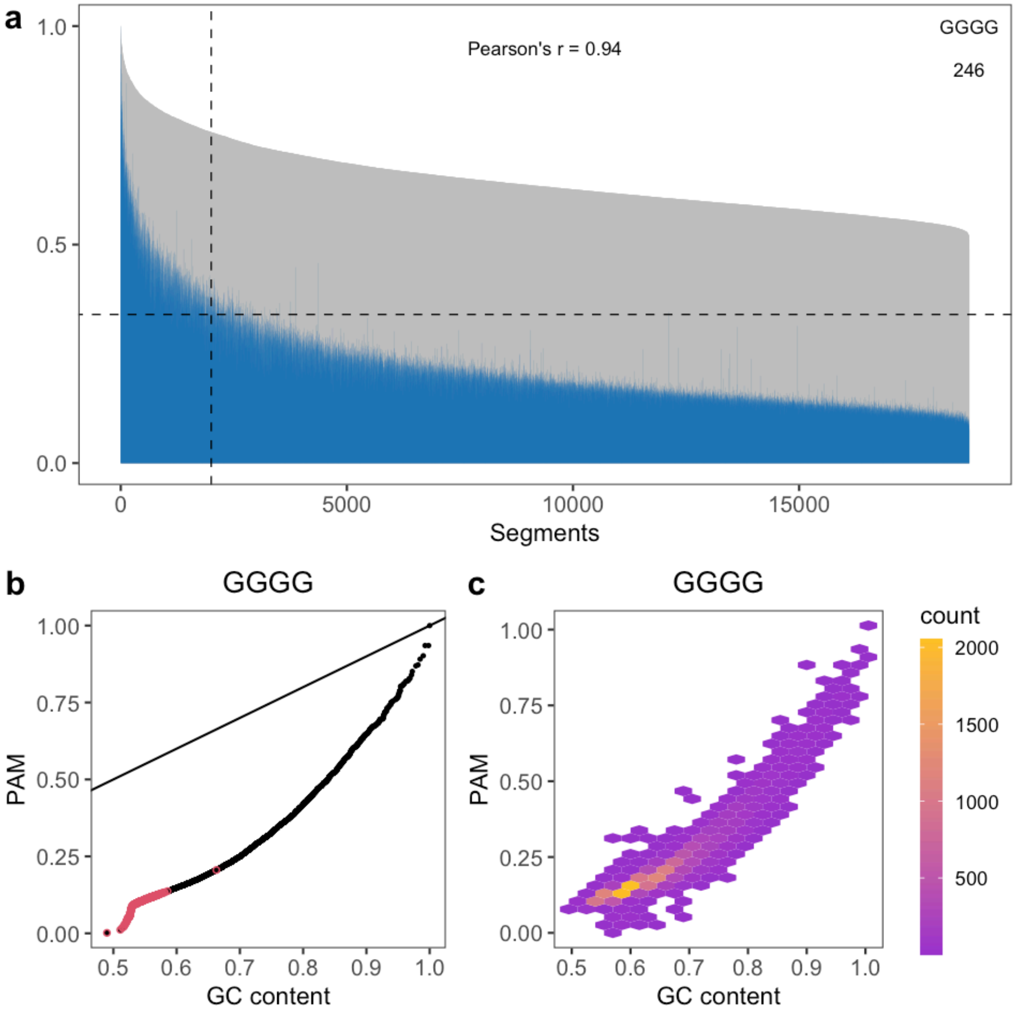

Supplementary Figure 43. Distribution of the GGGG PAM enriched regions compared with that of the GC-rich regions. **a**. PAM (shown on the top-right) content of 18,763 segments (excluding 1,237 segments with all N bases), respectively. The genome segments are ordered by decreasing GC content (grey), and the corresponding PAM content is plotted in the same order (blue). The segments to the left of the vertical dashed line are the top 2,000 segments for GC content, and the segments above the horizontal dashed line are the top 2,000 segments for the GGGG PAM. The number below the name of PAM (top-right) shows the number out of the top 2,000 PAM segments that are not in the top 2,000 GC-rich segments. The Pearson's r is the correlation between the GC content and the PAM content in all 18,763 segments. **b**. Quantile-quantile plot of the segments GC content v.s. PAM content distributions, where the red circles show the top 2,000 PAM segments, and the dashed line represents y = x. **c**. Hexbin plot of segments GC v.s. PAM content, where the color represents the count of points in each hexagon. GC and PAM content is normalized to the 0-1 range.

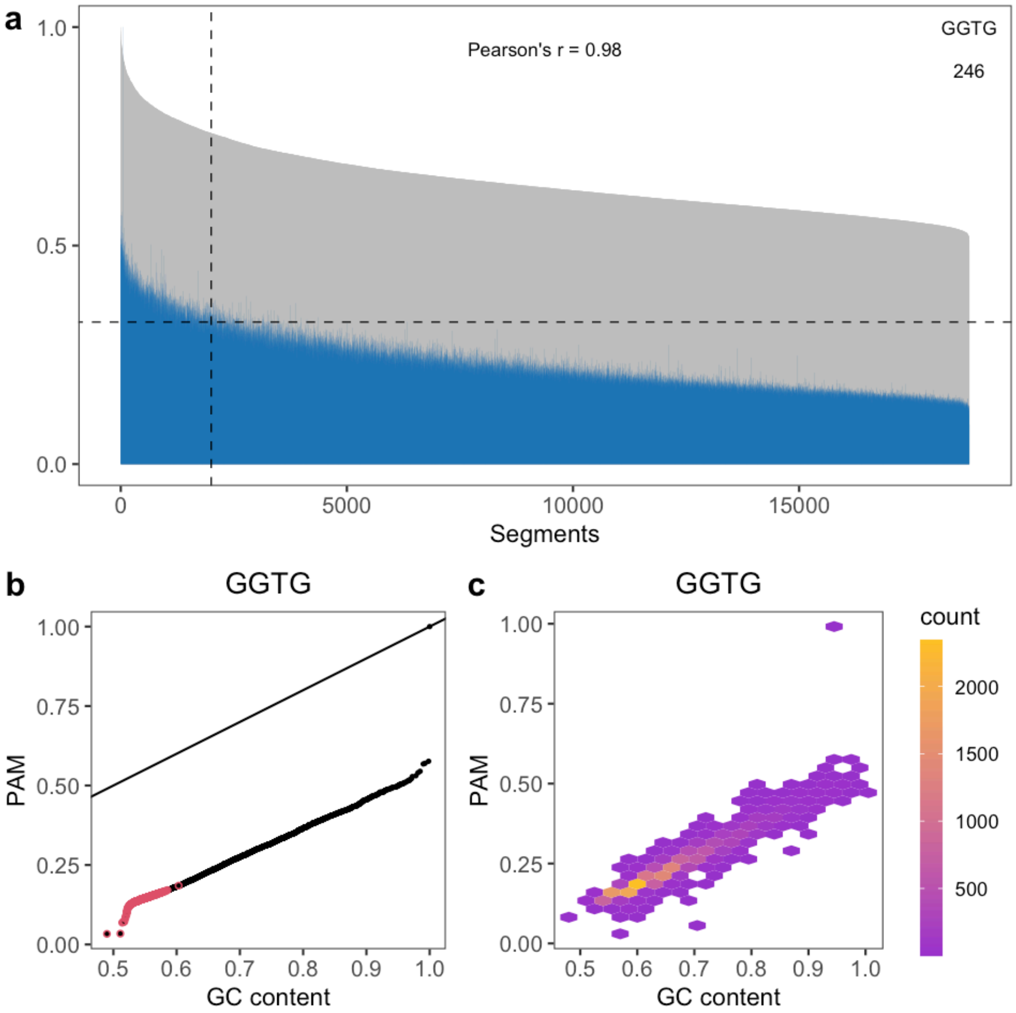

Supplementary Figure 44. Distribution of the GGTG PAM enriched regions compared with that of the GC-rich regions. **a**. PAM (shown on the top-right) content of 18,763 segments (excluding 1,237 segments with all N bases), respectively. The genome segments are ordered by decreasing GC content (grey), and the corresponding PAM content is plotted in the same order (blue). The segments to the left of the vertical dashed line are the top 2,000 segments for GC content, and the segments above the horizontal dashed line are the top 2,000 segments for the GGTG PAM. The number below the name of PAM (top-right) shows the number out of the top 2,000 PAM segments that are not in the top 2,000 GC-rich segments. The Pearson's r is the correlation between the GC content and the PAM content in all 18,763 segments. **b**. Quantile-quantile plot of the segments GC content v.s. PAM content distributions, where the red circles show the top 2,000 PAM segments, and the dashed line represents y = x. **c**. Hexbin plot of segments GC v.s. PAM content, where the color represents the count of points in each hexagon. GC and PAM content is normalized to the 0-1 range.

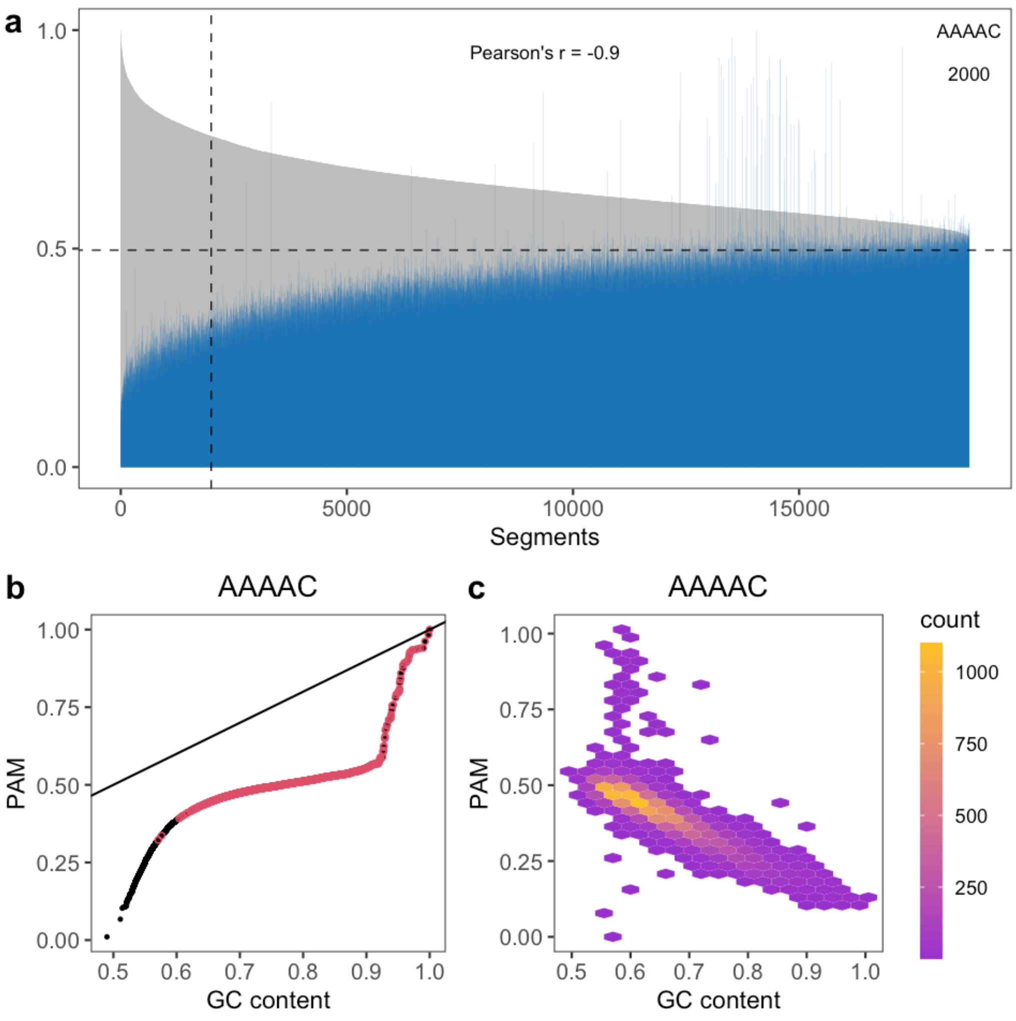

Supplementary Figure 45. Distribution of the AAAAC PAM enriched regions compared with that of the GC-rich regions. **a**. PAM (shown on the top-right) content of 18,763 segments (excluding 1,237 segments with all N bases), respectively. The genome segments are ordered by decreasing GC content (grey), and the corresponding PAM content is plotted in the same order (blue). The segments to the left of the vertical dashed line are the top 2,000 segments for GC content, and the segments above the horizontal dashed line are the top 2,000 segments for the AAAAC PAM. The number below the name of PAM (top-right) shows the number out of the top 2,000 PAM segments that are not in the top 2,000 GC-rich segments. The Pearson's r is the correlation between the GC content and the PAM content in all 18,763 segments. **b**. Quantile-quantile plot of the segments GC content v.s. PAM content distributions, where the red circles show the top 2,000 PAM segments, and the dashed line represents y = x. **c**. Hexbin plot of segments GC v.s. PAM content, where the color represents the count of points in each hexagon. GC and PAM content is normalized to the 0-1 range.

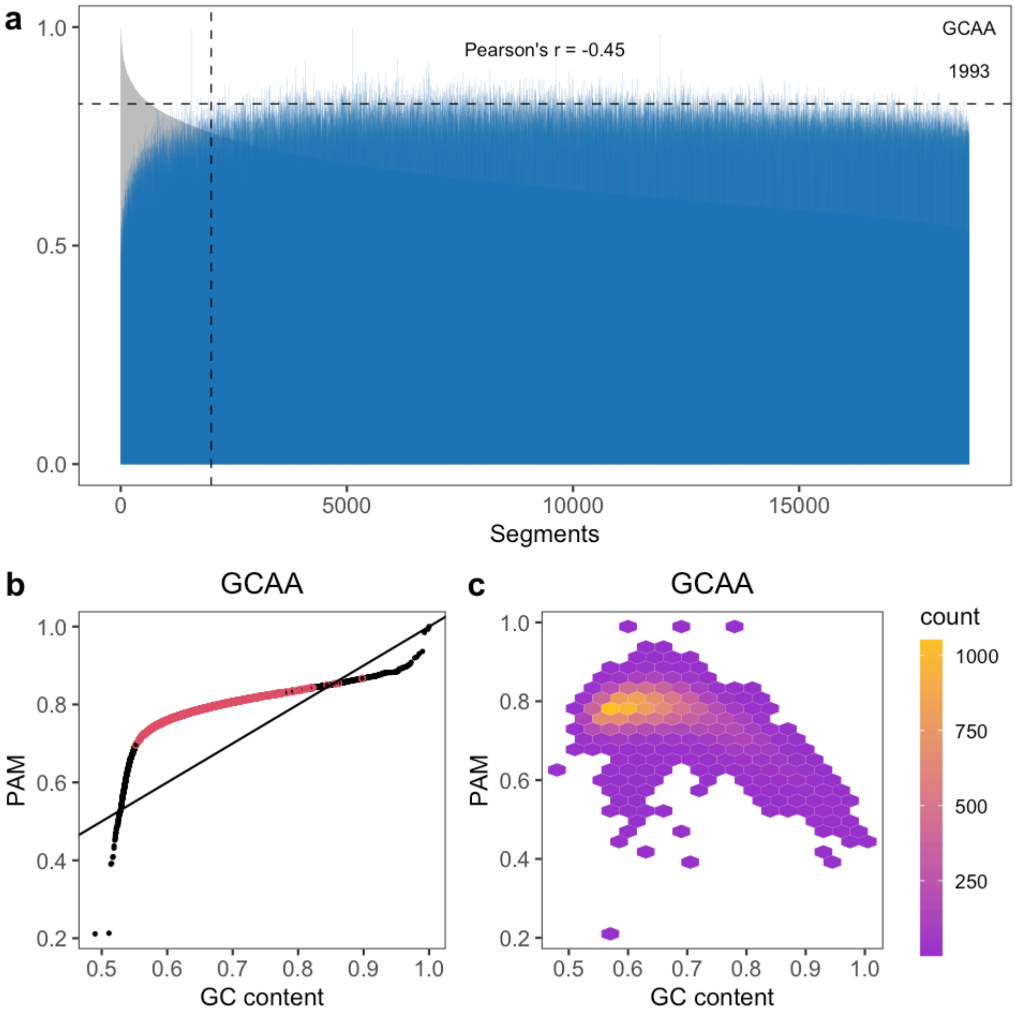

Supplementary Figure 46. Distribution of the GCAA PAM enriched regions compared with that of the GC-rich regions. **a**. PAM (shown on the top-right) content of 18,763 segments (excluding 1,237 segments with all N bases), respectively. The genome segments are ordered by decreasing GC content (grey), and the corresponding PAM content is plotted in the same order (blue). The segments to the left of the vertical dashed line are the top 2,000 segments for GC content, and the segments above the horizontal dashed line are the top 2,000 segments for the GCAA PAM. The number below the name of PAM (top-right) shows the number out of the top 2,000 PAM segments that are not in the top 2,000 GC-rich segments. The Pearson's r is the correlation between the GC content and the PAM content in all 18,763 segments. **b**. Quantile-quantile plot of the segments GC content v.s. PAM content distributions, where the red circles show the top 2,000 PAM segments, and the dashed line represents y = x. **c**. Hexbin plot of segments GC v.s. PAM content, where the color represents the count of points in each hexagon. GC and PAM content is normalized to the 0-1 range.

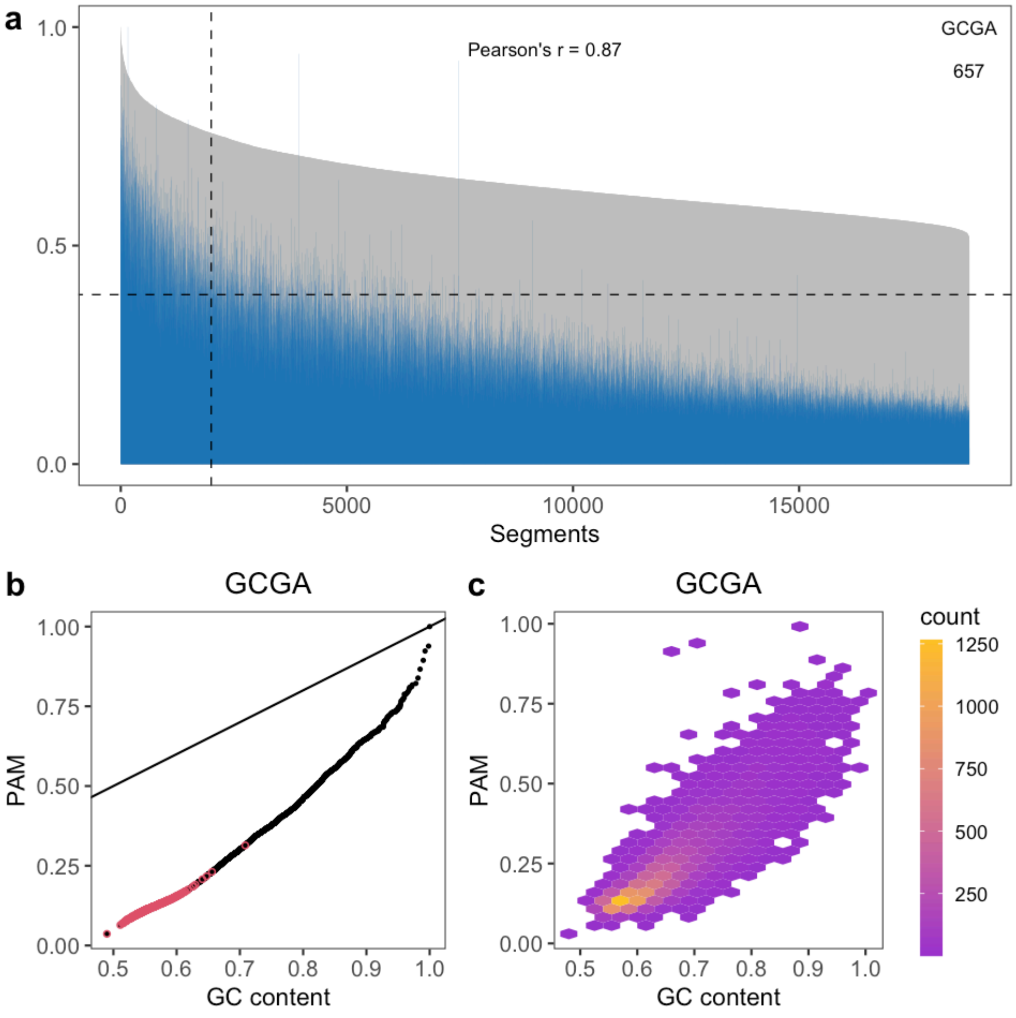

Supplementary Figure 47. Distribution of the GCGA PAM enriched regions compared with that of the GC-rich regions. **a**. PAM (shown on the top-right) content of 18,763 segments (excluding 1,237 segments with all N bases), respectively. The genome segments are ordered by decreasing GC content (grey), and the corresponding PAM content is plotted in the same order (blue). The segments to the left of the vertical dashed line are the top 2,000 segments for GC content, and the segments above the horizontal dashed line are the top 2,000 segments for the GCGA PAM. The number below the name of PAM (top-right) shows the number out of the top 2,000 PAM segments that are not in the top 2,000 GC-rich segments. The Pearson's r is the correlation between the GC content and the PAM content in all 18,763 segments. **b**. Quantile-quantile plot of the segments GC content v.s. PAM content distributions, where the red circles show the top 2,000 PAM segments, and the dashed line represents y = x. **c**. Hexbin plot of segments GC v.s. PAM content, where the color represents the count of points in each hexagon. GC and PAM content is normalized to the 0-1 range.

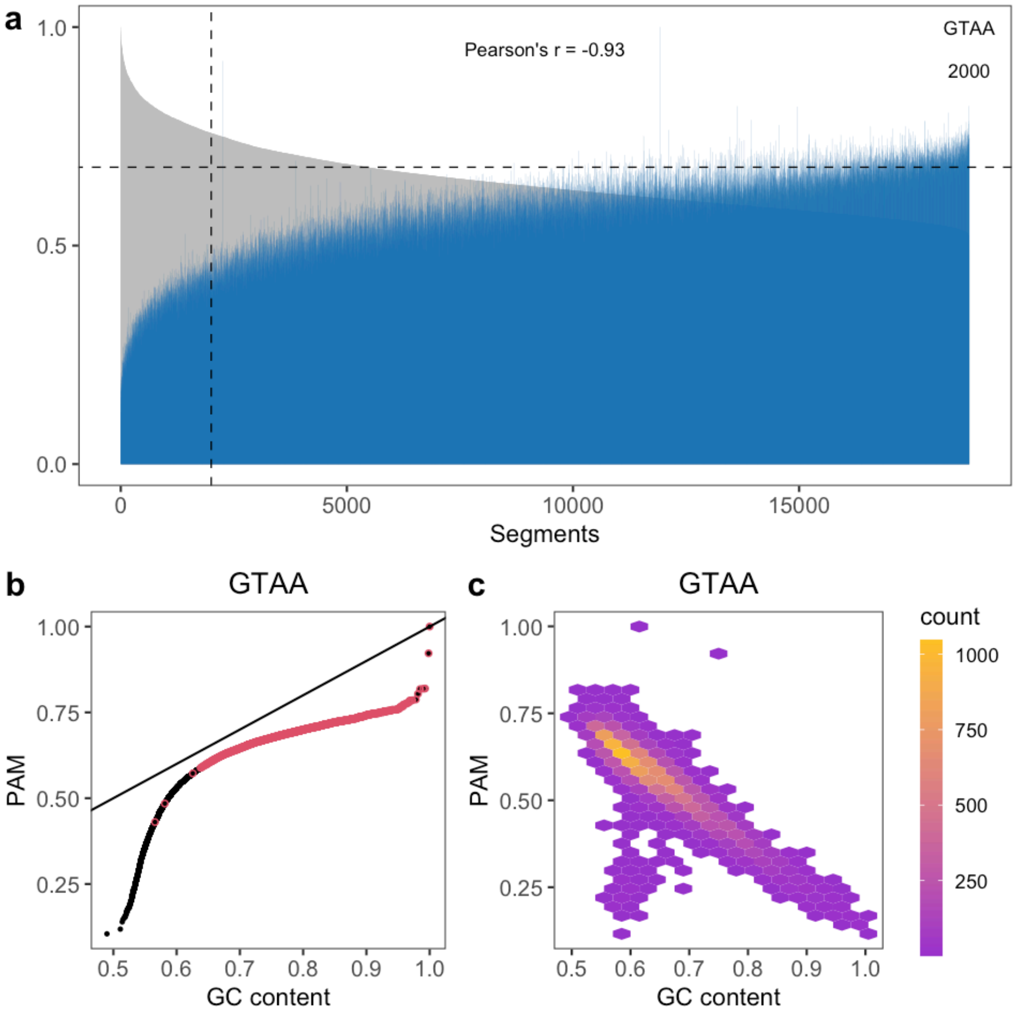

Supplementary Figure 48. Distribution of the GTAA PAM enriched regions compared with that of the GC-rich regions. **a**. PAM (shown on the top-right) content of 18,763 segments (excluding 1,237 segments with all N bases), respectively. The genome segments are ordered by decreasing GC content (grey), and the corresponding PAM content is plotted in the same order (blue). The segments to the left of the vertical dashed line are the top 2,000 segments for GC content, and the segments above the horizontal dashed line are the top 2,000 segments for the GTAA PAM. The number below the name of PAM (top-right) shows the number out of the top 2,000 PAM segments that are not in the top 2,000 GC-rich segments. The Pearson's r is the correlation between the GC content and the PAM content in all 18,763 segments. **b**. Quantile-quantile plot of the segments GC content v.s. PAM content distributions, where the red circles show the top 2,000 PAM segments, and the dashed line represents y = x. **c**. Hexbin plot of segments GC v.s. PAM content, where the color represents the count of points in each hexagon. GC and PAM content is normalized to the 0-1 range.

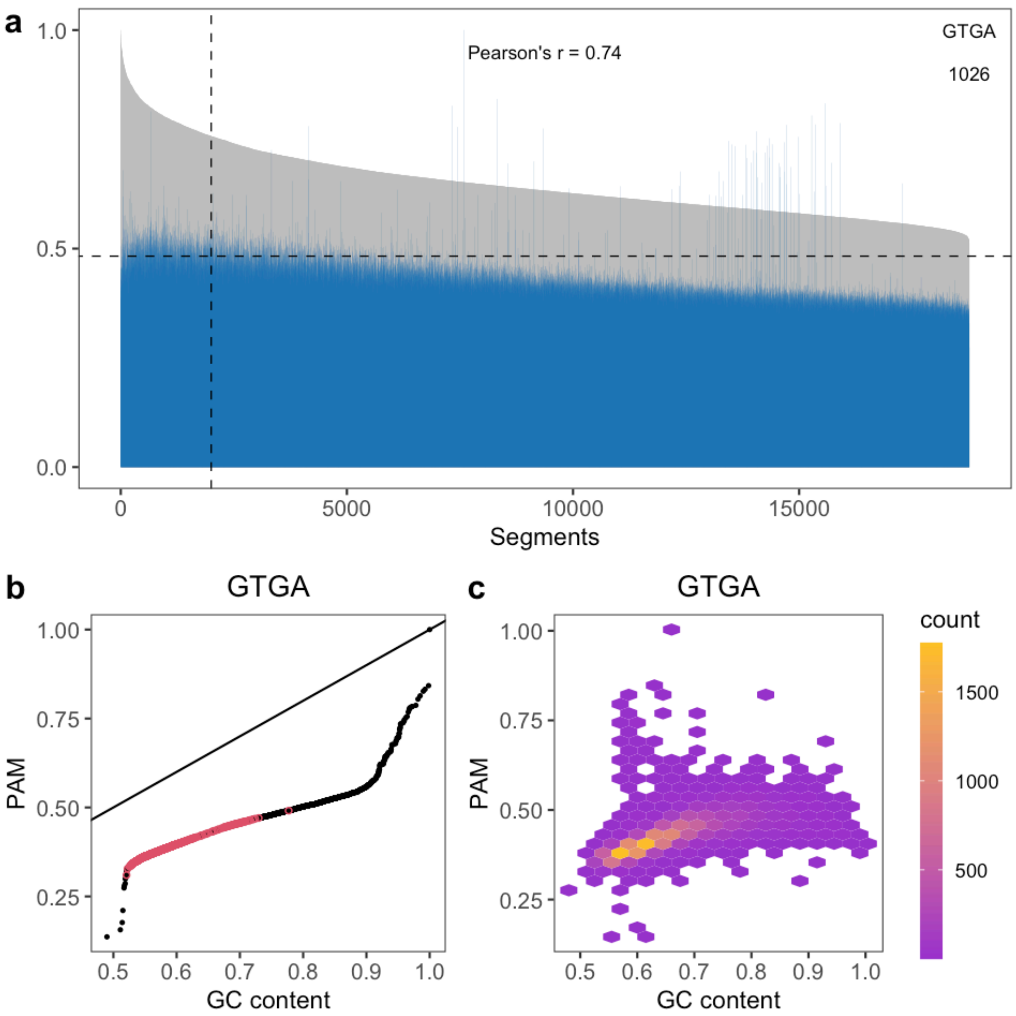

Supplementary Figure 49. Distribution of the GTGA PAM enriched regions compared with that of the GC-rich regions. **a**. PAM (shown on the top-right) content of 18,763 segments (excluding 1,237 segments with all N bases), respectively. The genome segments are ordered by decreasing GC content (grey), and the corresponding PAM content is plotted in the same order (blue). The segments to the left of the vertical dashed line are the top 2,000 segments for GC content, and the segments above the horizontal dashed line are the top 2,000 segments for the GTGA PAM. The number below the name of PAM (top-right) shows the number out of the top 2,000 PAM segments that are not in the top 2,000 GC-rich segments. The Pearson's r is the correlation between the GC content and the PAM content in all 18,763 segments. **b**. Quantile-quantile plot of the segments GC content v.s. PAM content distributions, where the red circles show the top 2,000 PAM segments, and the dashed line represents y = x. **c**. Hexbin plot of segments GC v.s. PAM content, where the color represents the count of points in each hexagon. GC and PAM content is normalized to the 0-1 range.

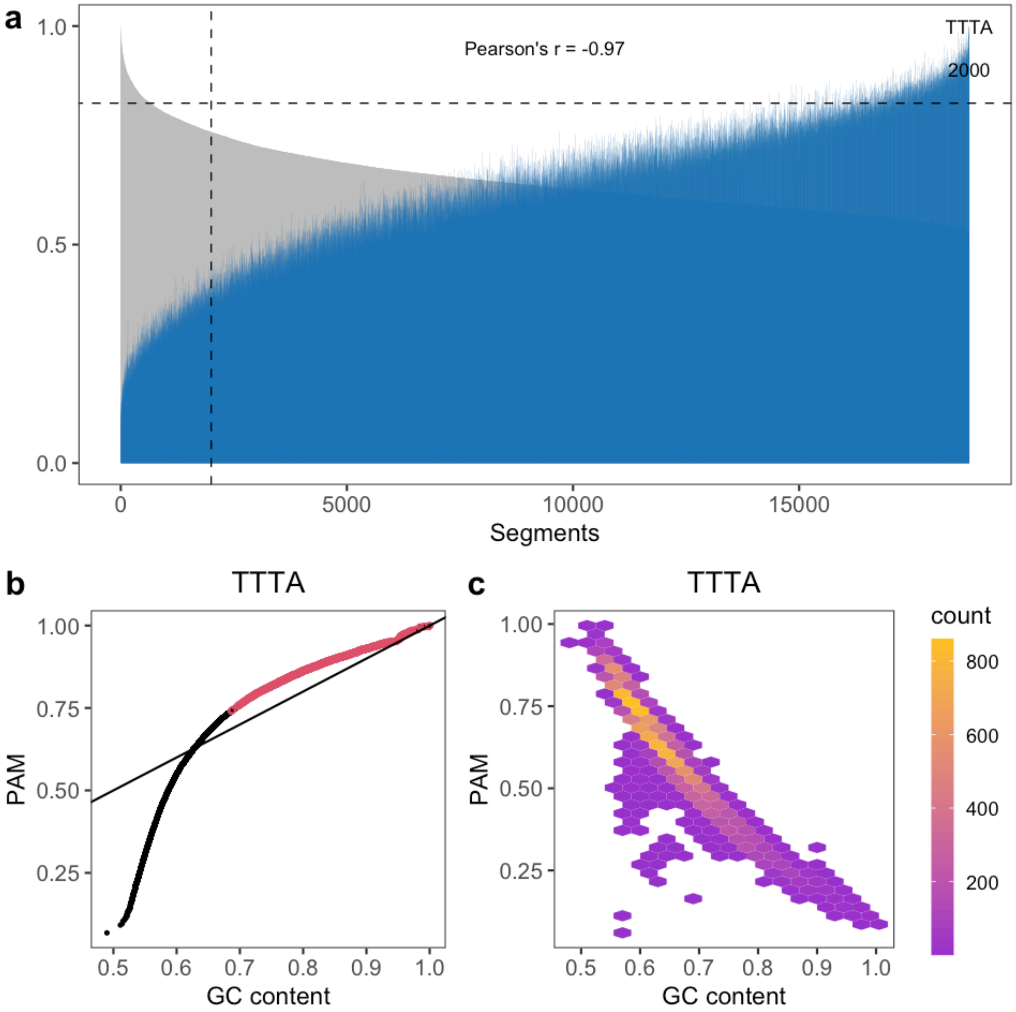

Supplementary Figure 50. Distribution of the TTTA PAM enriched regions compared with that of the GC-rich regions. **a**. PAM (shown on the top-right) content of 18,763 segments (excluding 1,237 segments with all N bases), respectively. The genome segments are ordered by decreasing GC content (grey), and the corresponding PAM content is plotted in the same order (blue). The segments to the left of the vertical dashed line are the top 2,000 segments for GC content, and the segments above the horizontal dashed line are the top 2,000 segments for the TTTA PAM. The number below the name of PAM (top-right) shows the number out of the top 2,000 PAM segments that are not in the top 2,000 GC-rich segments. The Pearson's r is the correlation between the GC content and the PAM content in all 18,763 segments. **b**. Quantile-quantile plot of the segments GC content v.s. PAM content distributions, where the red circles show the top 2,000 PAM segments, and the dashed line represents y = x. **c**. Hexbin plot of segments GC v.s. PAM content, where the color represents the count of points in each hexagon. GC and PAM content is normalized to the 0-1 range.

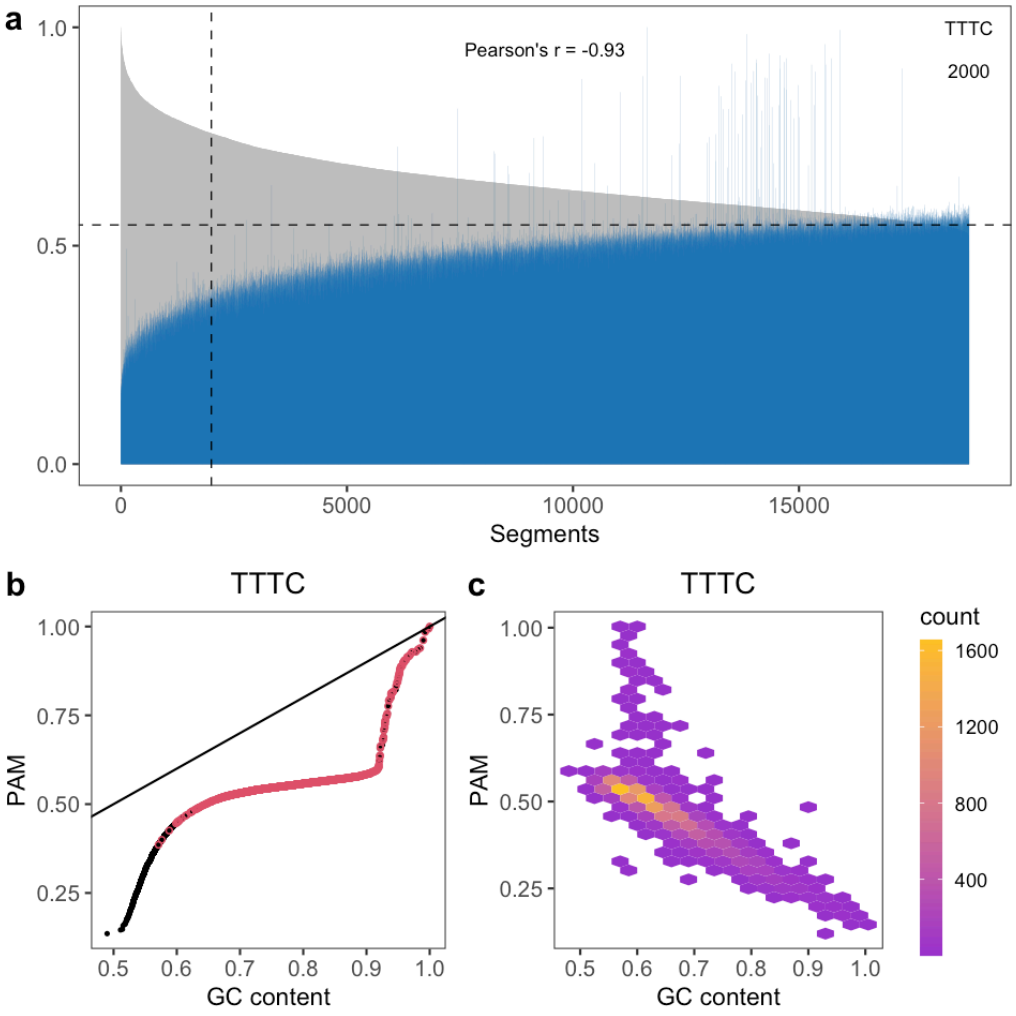

Supplementary Figure 51. Distribution of the TTTC PAM enriched regions compared with that of the GC-rich regions. **a**. PAM (shown on the top-right) content of 18,763 segments (excluding 1,237 segments with all N bases), respectively. The genome segments are ordered by decreasing GC content (grey), and the corresponding PAM content is plotted in the same order (blue). The segments to the left of the vertical dashed line are the top 2,000 segments for GC content, and the segments above the horizontal dashed line are the top 2,000 segments for the TTTC PAM. The number below the name of PAM (top-right) shows the number out of the top 2,000 PAM segments that are not in the top 2,000 GC-rich segments. The Pearson's r is the correlation between the GC content and the PAM content in all 18,763 segments. **b**. Quantile-quantile plot of the segments GC content v.s. PAM content distributions, where the red circles show the top 2,000 PAM segments, and the dashed line represents y = x. **c**. Hexbin plot of segments GC v.s. PAM content, where the color represents the count of points in each hexagon. GC and PAM content is normalized to the 0-1 range.

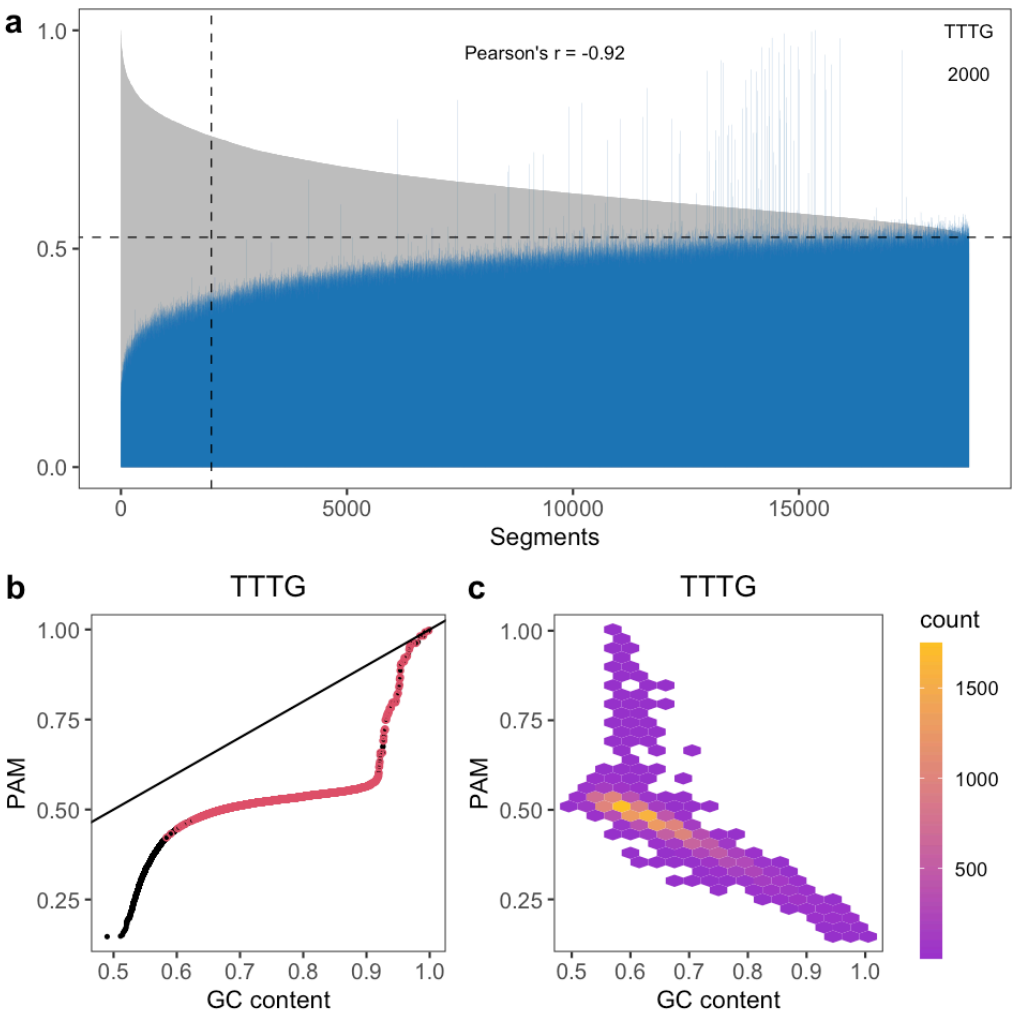

Supplementary Figure 52. Distribution of the TTTG PAM enriched regions compared with that of the GC-rich regions. **a**. PAM (shown on the top-right) content of 18,763 segments (excluding 1,237 segments with all N bases), respectively. The genome segments are ordered by decreasing GC content (grey), and the corresponding PAM content is plotted in the same order (blue). The segments to the left of the vertical dashed line are the top 2,000 segments for GC content, and the segments above the horizontal dashed line are the top 2,000 segments for the TTTG PAM. The number below the name of PAM (top-right) shows the number out of the top 2,000 PAM segments that are not in the top 2,000 GC-rich segments. The Pearson's r is the correlation between the GC content and the PAM content in all 18,763 segments. **b**. Quantile-quantile plot of the segments GC content v.s. PAM content distributions, where the red circles show the top 2,000 PAM segments, and the dashed line represents y = x. **c**. Hexbin plot of segments GC v.s. PAM content, where the color represents the count of points in each hexagon. GC and PAM content is normalized to the 0-1 range.

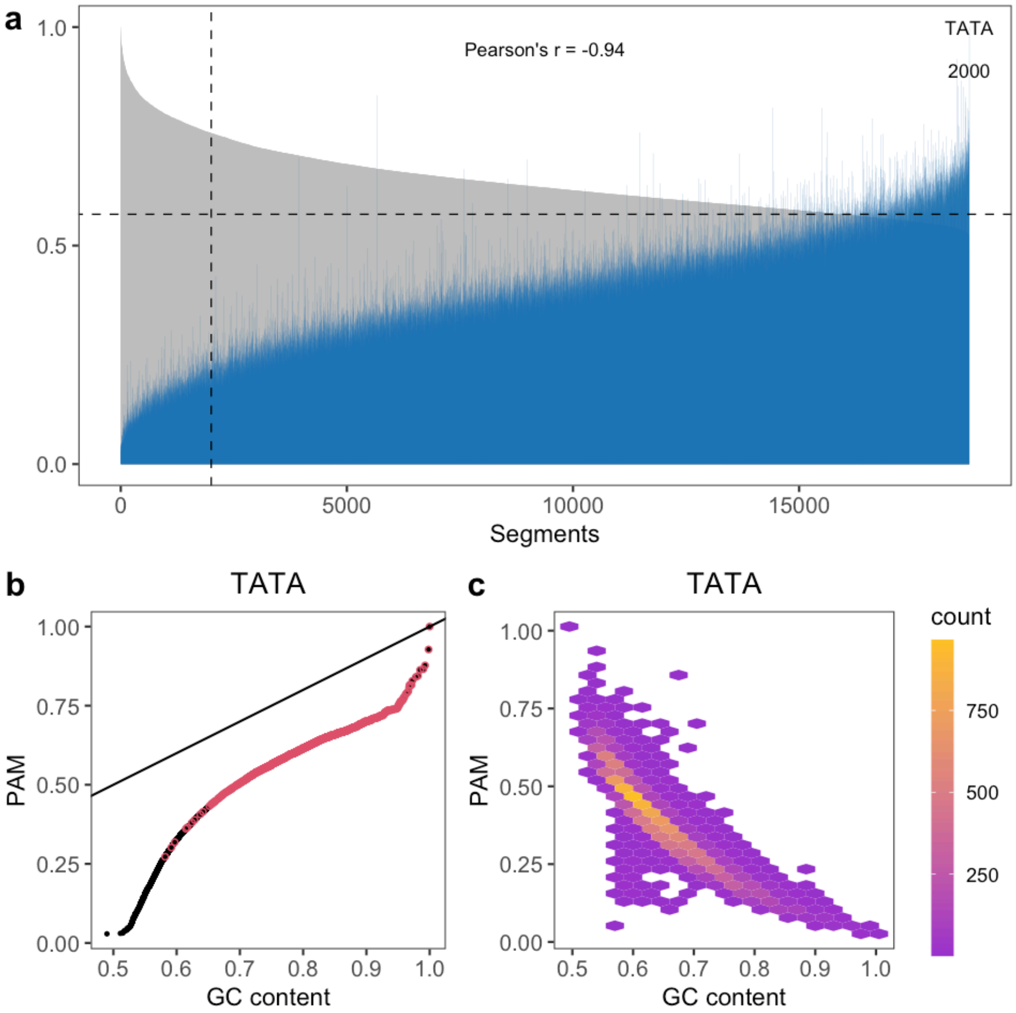

Supplementary Figure 53. Distribution of the TATA PAM enriched regions compared with that of the GC-rich regions. **a**. PAM (shown on the top-right) content of 18,763 segments (excluding 1,237 segments with all N bases), respectively. The genome segments are ordered by decreasing GC content (grey), and the corresponding PAM content is plotted in the same order (blue). The segments to the left of the vertical dashed line are the top 2,000 segments for GC content, and the segments above the horizontal dashed line are the top 2,000 segments for the TATA PAM. The number below the name of PAM (top-right) shows the number out of the top 2,000 PAM segments that are not in the top 2,000 GC-rich segments. The Pearson's r is the correlation between the GC content and the PAM content in all 18,763 segments. **b**. Quantile-quantile plot of the segments GC content v.s. PAM content distributions, where the red circles show the top 2,000 PAM segments, and the dashed line represents y = x. **c**. Hexbin plot of segments GC v.s. PAM content, where the color represents the count of points in each hexagon. GC and PAM content is normalized to the 0-1 range.

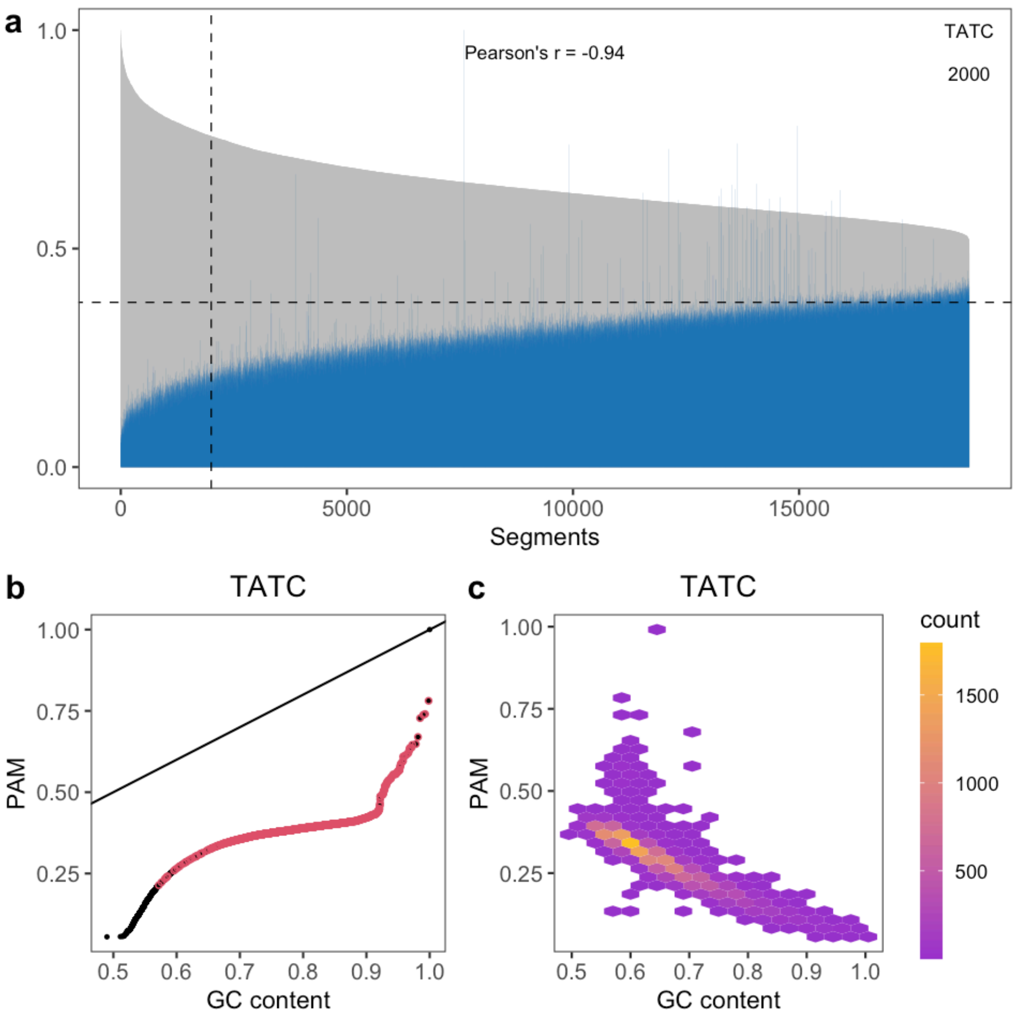

Supplementary Figure 54. Distribution of the TATC PAM enriched regions compared with that of the GC-rich regions. **a**. PAM (shown on the top-right) content of 18,763 segments (excluding 1,237 segments with all N bases), respectively. The genome segments are ordered by decreasing GC content (grey), and the corresponding PAM content is plotted in the same order (blue). The segments to the left of the vertical dashed line are the top 2,000 segments for GC content, and the segments above the horizontal dashed line are the top 2,000 segments for the TATC PAM. The number below the name of PAM (top-right) shows the number out of the top 2,000 PAM segments that are not in the top 2,000 GC-rich segments. The Pearson's r is the correlation between the GC content and the PAM content in all 18,763 segments. **b**. Quantile-quantile plot of the segments GC content v.s. PAM content distributions, where the red circles show the top 2,000 PAM segments, and the dashed line represents y = x. **c**. Hexbin plot of segments GC v.s. PAM content, where the color represents the count of points in each hexagon. GC and PAM content is normalized to the 0-1 range.

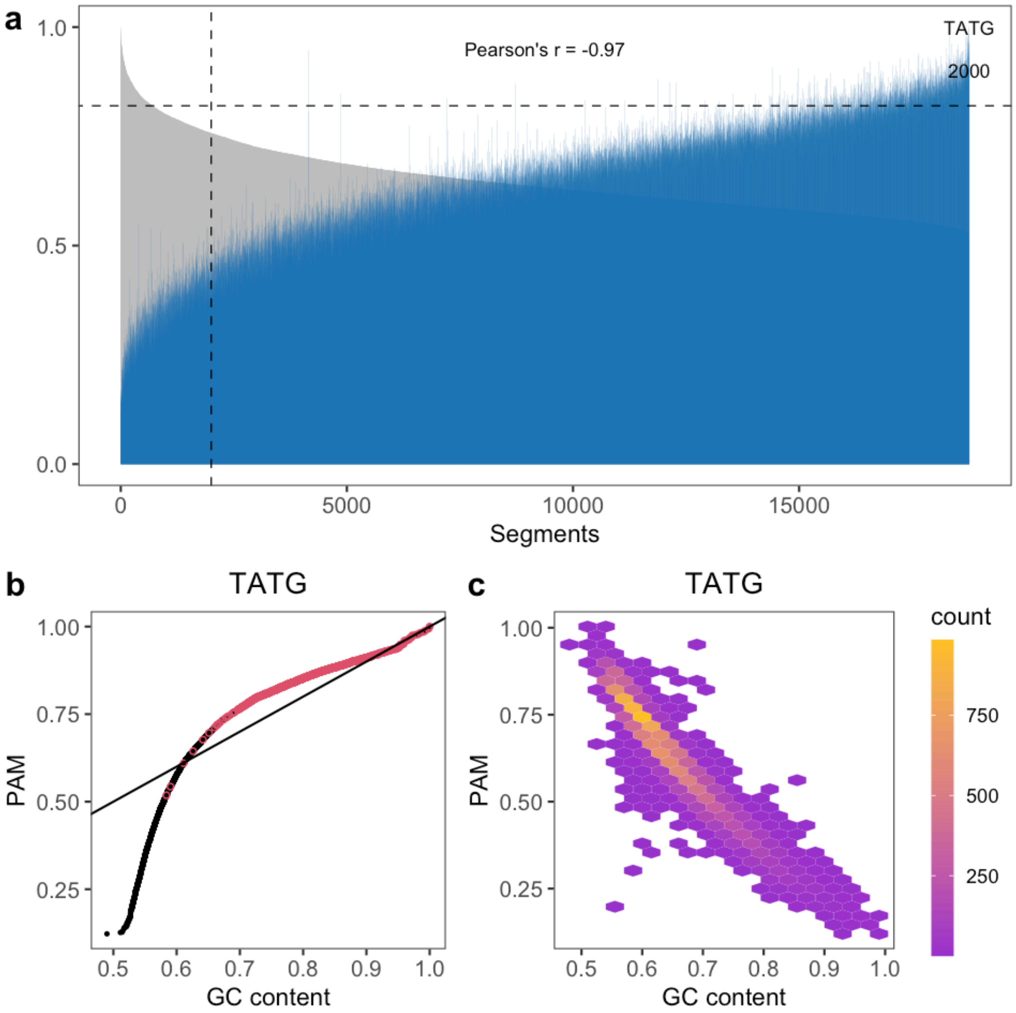

Supplementary Figure 55. Distribution of the TATG PAM enriched regions compared with that of the GC-rich regions. **a**. PAM (shown on the top-right) content of 18,763 segments (excluding 1,237 segments with all N bases), respectively. The genome segments are ordered by decreasing GC content (grey), and the corresponding PAM content is plotted in the same order (blue). The segments to the left of the vertical dashed line are the top 2,000 segments for GC content, and the segments above the horizontal dashed line are the top 2,000 segments for the TATG PAM. The number below the name of PAM (top-right) shows the number out of the top 2,000 PAM segments that are not in the top 2,000 GC-rich segments. The Pearson's r is the correlation between the GC content and the PAM content in all 18,763 segments. **b**. Quantile-quantile plot of the segments GC content v.s. PAM content distributions, where the red circles show the top 2,000 PAM segments, and the dashed line represents y = x. **c**. Hexbin plot of segments GC v.s. PAM content, where the color represents the count of points in each hexagon. GC and PAM content is normalized to the 0-1 range.

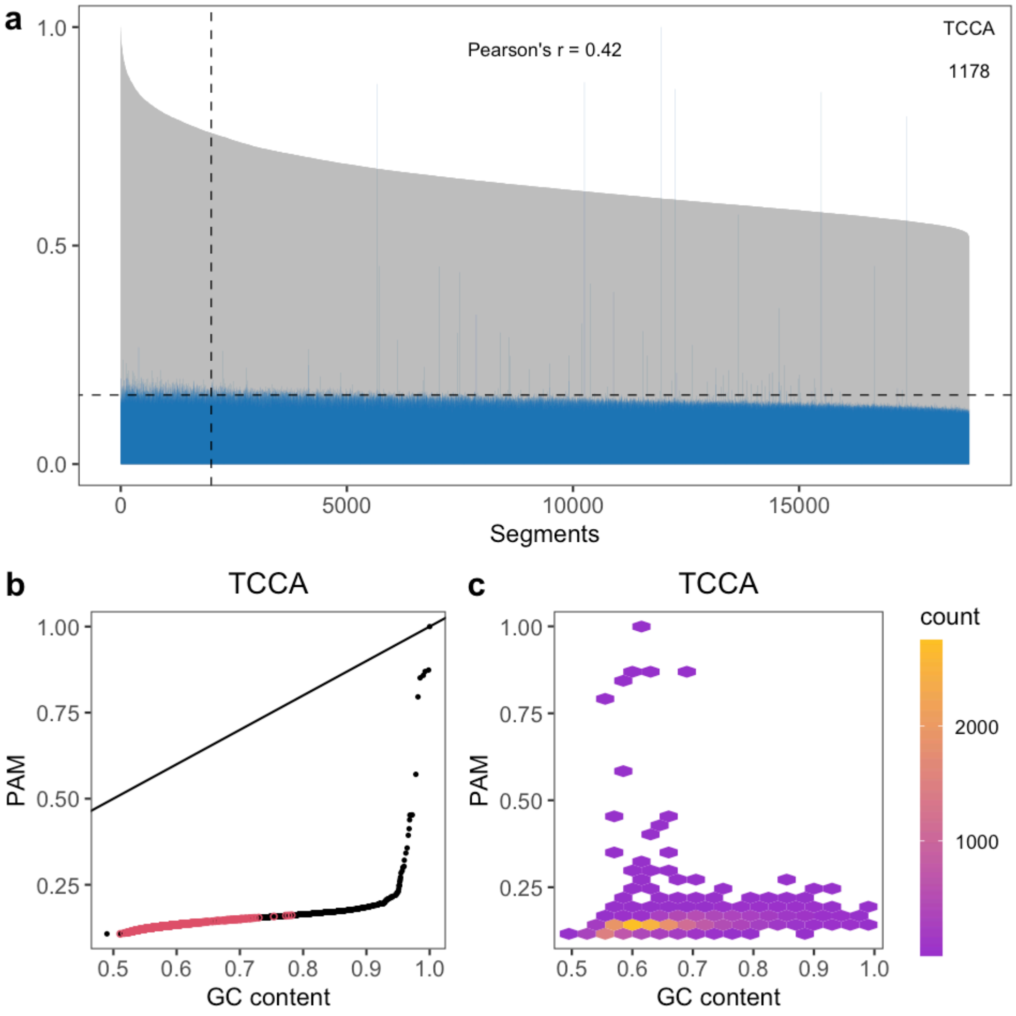

Supplementary Figure 56. Distribution of the TCCA PAM enriched regions compared with that of the GC-rich regions. **a**. PAM (shown on the top-right) content of 18,763 segments (excluding 1,237 segments with all N bases), respectively. The genome segments are ordered by decreasing GC content (grey), and the corresponding PAM content is plotted in the same order (blue). The segments to the left of the vertical dashed line are the top 2,000 segments for GC content, and the segments above the horizontal dashed line are the top 2,000 segments for the TCCA PAM. The number below the name of PAM (top-right) shows the number out of the top 2,000 PAM segments that are not in the top 2,000 GC-rich segments. The Pearson's r is the correlation between the GC content and the PAM content in all 18,763 segments. **b**. Quantile-quantile plot of the segments GC content v.s. PAM content distributions, where the red circles show the top 2,000 PAM segments, and the dashed line represents y = x. **c**. Hexbin plot of segments GC v.s. PAM content, where the color represents the count of points in each hexagon. GC and PAM content is normalized to the 0-1 range.

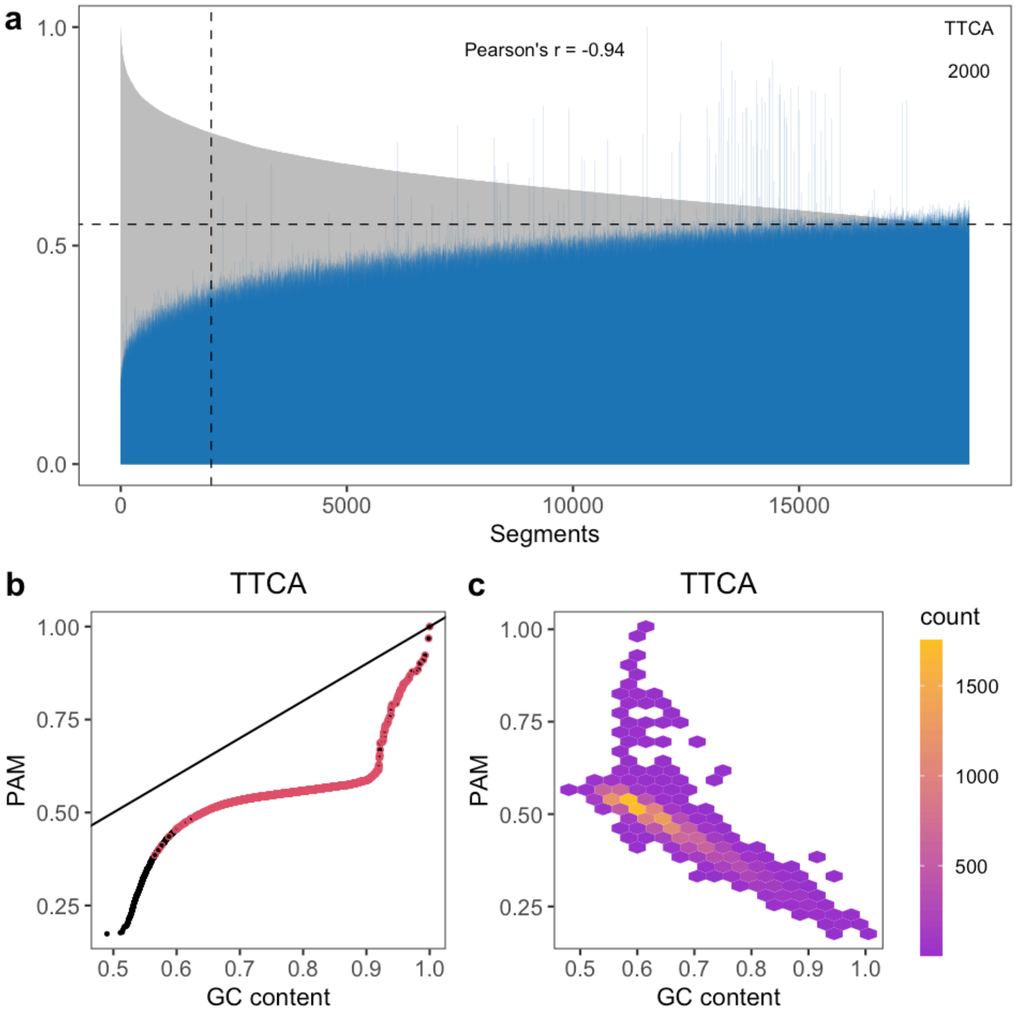

Supplementary Figure 57. Distribution of the TTCA PAM enriched regions compared with that of the GC-rich regions. **a**. PAM (shown on the top-right) content of 18,763 segments (excluding 1,237 segments with all N bases), respectively. The genome segments are ordered by decreasing GC content (grey), and the corresponding PAM content is plotted in the same order (blue). The segments to the left of the vertical dashed line are the top 2,000 segments for GC content, and the segments above the horizontal dashed line are the top 2,000 segments for the TTCA PAM. The number below the name of PAM (top-right) shows the number out of the top 2,000 PAM segments that are not in the top 2,000 GC-rich segments. The Pearson's r is the correlation between the GC content and the PAM content in all 18,763 segments. **b**. Quantile-quantile plot of the segments GC content v.s. PAM content distributions, where the red circles show the top 2,000 PAM segments, and the dashed line represents y = x. **c**. Hexbin plot of segments GC v.s. PAM content, where the color represents the count of points in each hexagon. GC and PAM content is normalized to the 0-1 range.

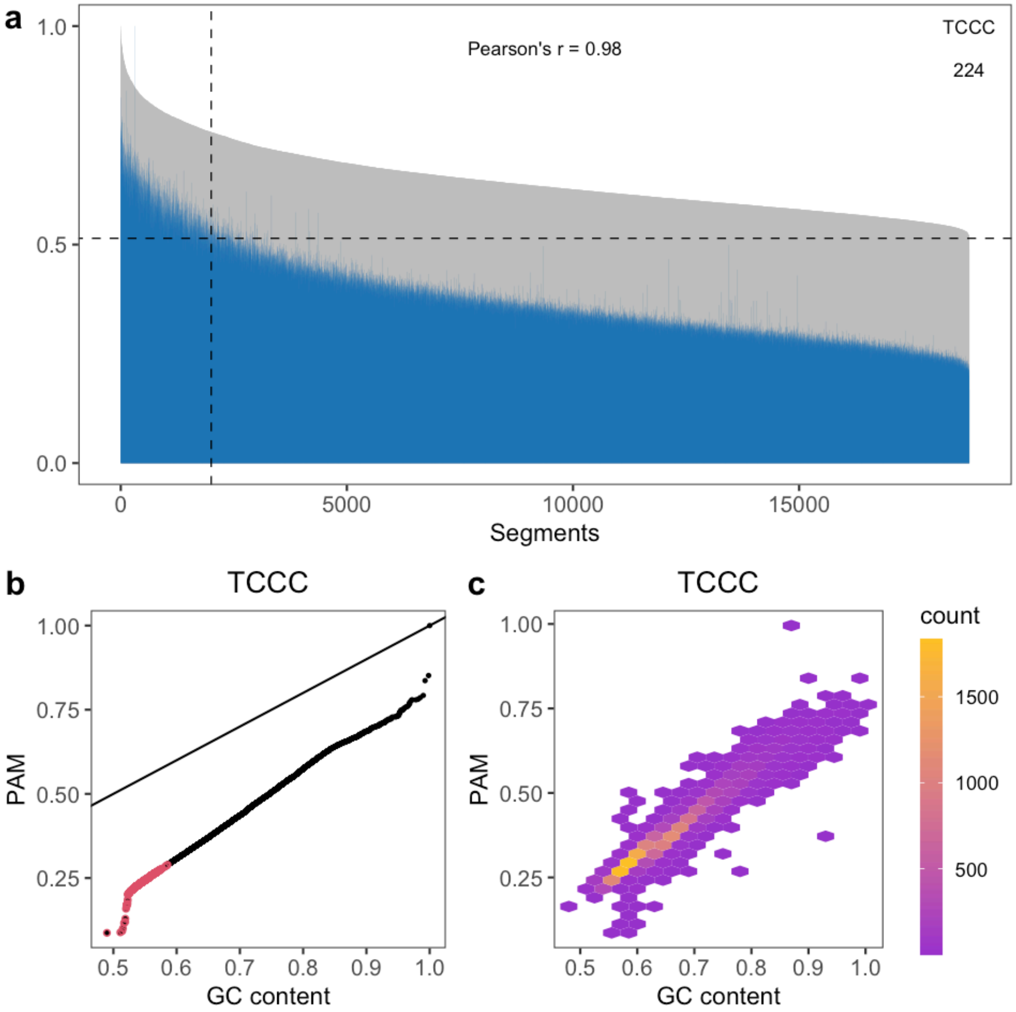

Supplementary Figure 58. Distribution of the TCCC PAM enriched regions compared with that of the GC-rich regions. **a**. PAM (shown on the top-right) content of 18,763 segments (excluding 1,237 segments with all N bases), respectively. The genome segments are ordered by decreasing GC content (grey), and the corresponding PAM content is plotted in the same order (blue). The segments to the left of the vertical dashed line are the top 2,000 segments for GC content, and the segments above the horizontal dashed line are the top 2,000 segments for the TCCC PAM. The number below the name of PAM (top-right) shows the number out of the top 2,000 PAM segments that are not in the top 2,000 GC-rich segments. The Pearson's r is the correlation between the GC content and the PAM content in all 18,763 segments. **b**. Quantile-quantile plot of the segments GC content v.s. PAM content distributions, where the red circles show the top 2,000 PAM segments, and the dashed line represents y = x. **c**. Hexbin plot of segments GC v.s. PAM content, where the color represents the count of points in each hexagon. GC and PAM content is normalized to the 0-1 range.

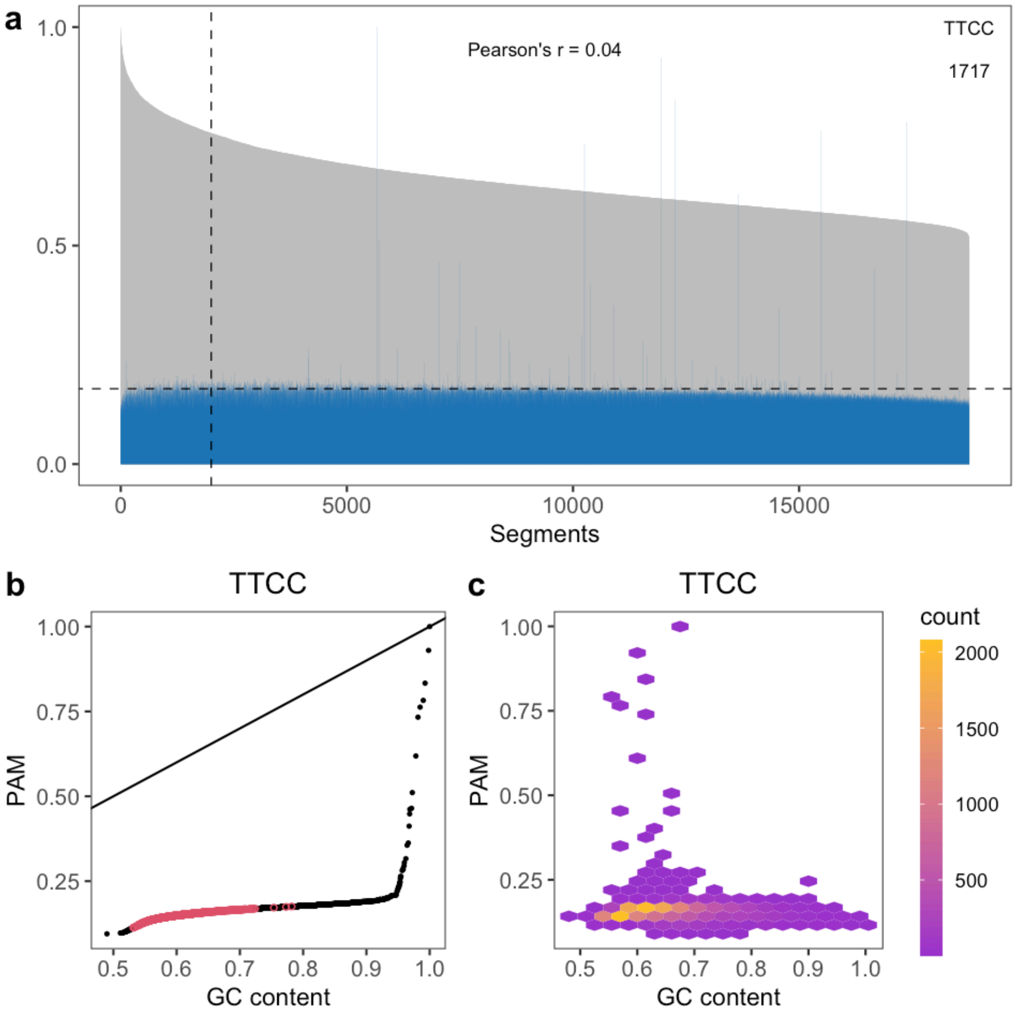

Supplementary Figure 59. Distribution of the TTCC PAM enriched regions compared with that of the GC-rich regions. **a**. PAM (shown on the top-right) content of 18,763 segments (excluding 1,237 segments with all N bases), respectively. The genome segments are ordered by decreasing GC content (grey), and the corresponding PAM content is plotted in the same order (blue). The segments to the left of the vertical dashed line are the top 2,000 segments for GC content, and the segments above the horizontal dashed line are the top 2,000 segments for the TTCC PAM. The number below the name of PAM (top-right) shows the number out of the top 2,000 PAM segments that are not in the top 2,000 GC-rich segments. The Pearson's r is the correlation between the GC content and the PAM content in all 18,763 segments. **b**. Quantile-quantile plot of the segments GC content v.s. PAM content distributions, where the red circles show the top 2,000 PAM segments, and the dashed line represents y = x. **c**. Hexbin plot of segments GC v.s. PAM content, where the color represents the count of points in each hexagon. GC and PAM content is normalized to the 0-1 range.

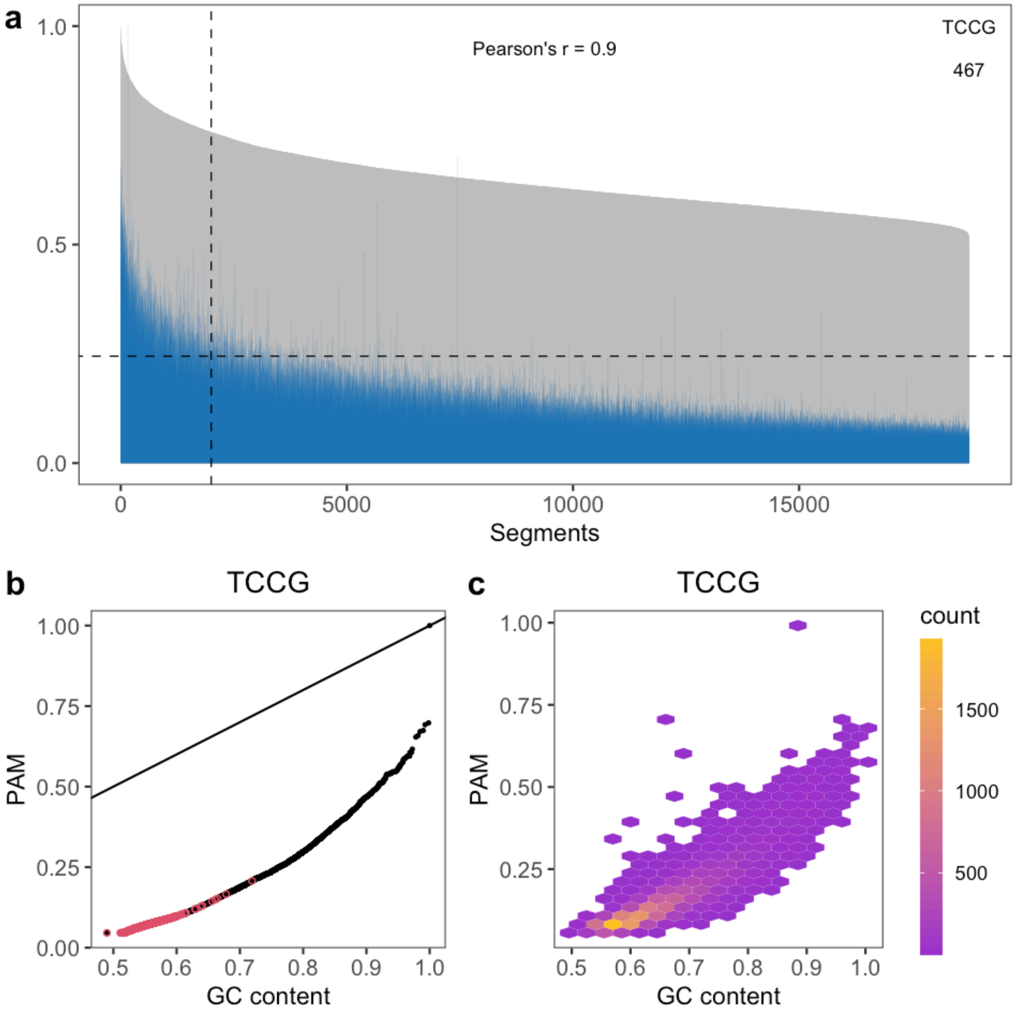

Supplementary Figure 60. Distribution of the TCCG PAM enriched regions compared with that of the GC-rich regions. **a**. PAM (shown on the top-right) content of 18,763 segments (excluding 1,237 segments with all N bases), respectively. The genome segments are ordered by decreasing GC content (grey), and the corresponding PAM content is plotted in the same order (blue). The segments to the left of the vertical dashed line are the top 2,000 segments for GC content, and the segments above the horizontal dashed line are the top 2,000 segments for the TCCG PAM. The number below the name of PAM (top-right) shows the number out of the top 2,000 PAM segments that are not in the top 2,000 GC-rich segments. The Pearson's r is the correlation between the GC content and the PAM content in all 18,763 segments. **b**. Quantile-quantile plot of the segments GC content v.s. PAM content distributions, where the red circles show the top 2,000 PAM segments, and the dashed line represents y = x. **c**. Hexbin plot of segments GC v.s. PAM content, where the color represents the count of points in each hexagon. GC and PAM content is normalized to the 0-1 range.

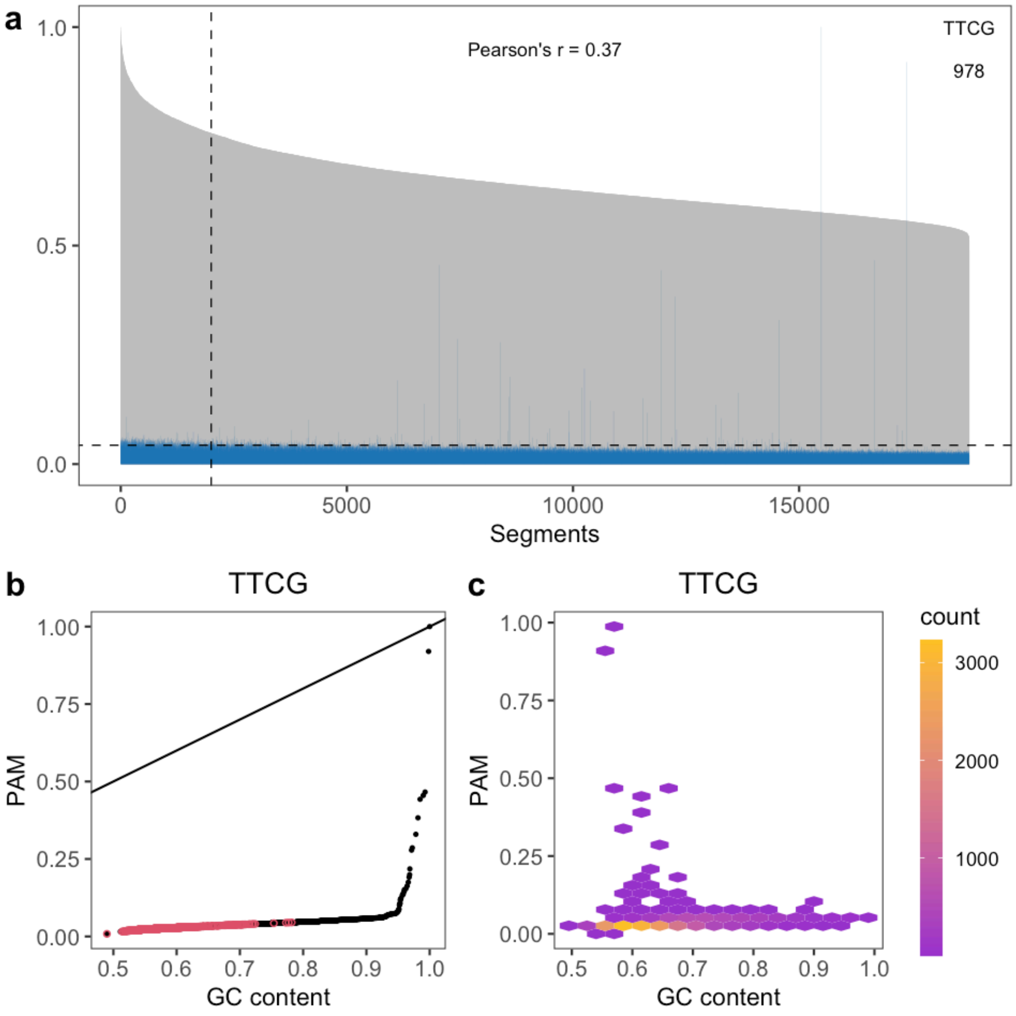

Supplementary Figure 61. Distribution of the TTCG PAM enriched regions compared with that of the GC-rich regions. **a**. PAM (shown on the top-right) content of 18,763 segments (excluding 1,237 segments with all N bases), respectively. The genome segments are ordered by decreasing GC content (grey), and the corresponding PAM content is plotted in the same order (blue). The segments to the left of the vertical dashed line are the top 2,000 segments for GC content, and the segments above the horizontal dashed line are the top 2,000 segments for the TTCG PAM. The number below the name of PAM (top-right) shows the number out of the top 2,000 PAM segments that are not in the top 2,000 GC-rich segments. The Pearson's r is the correlation between the GC content and the PAM content in all 18,763 segments. **b**. Quantile-quantile plot of the segments GC content v.s. PAM content distributions, where the red circles show the top 2,000 PAM segments, and the dashed line represents y = x. **c**. Hexbin plot of segments GC v.s. PAM content, where the color represents the count of points in each hexagon. GC and PAM content is normalized to the 0-1 range.

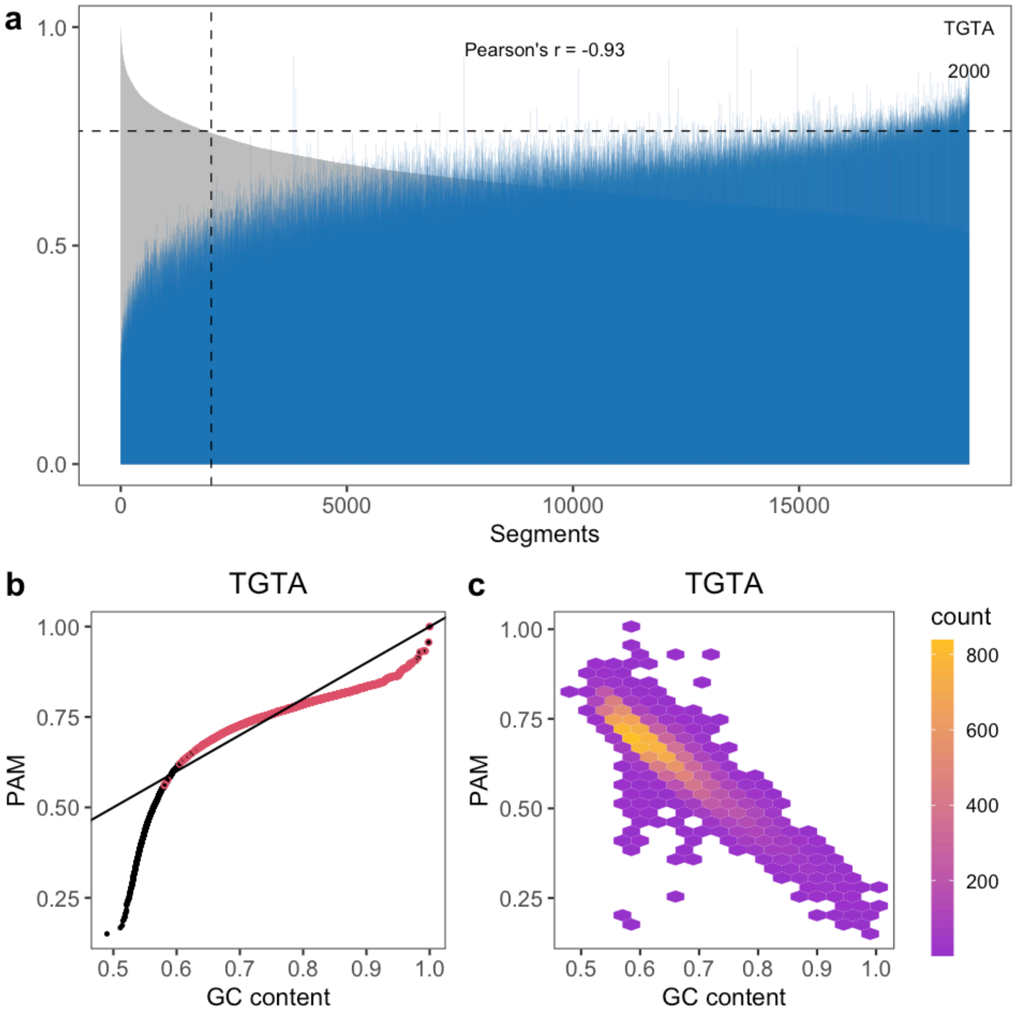

Supplementary Figure 62. Distribution of the TGTA PAM enriched regions compared with that of the GC-rich regions. **a**. PAM (shown on the top-right) content of 18,763 segments (excluding 1,237 segments with all N bases), respectively. The genome segments are ordered by decreasing GC content (grey), and the corresponding PAM content is plotted in the same order (blue). The segments to the left of the vertical dashed line are the top 2,000 segments for GC content, and the segments above the horizontal dashed line are the top 2,000 segments for the TGTA PAM. The number below the name of PAM (top-right) shows the number out of the top 2,000 PAM segments that are not in the top 2,000 GC-rich segments. The Pearson's r is the correlation between the GC content and the PAM content in all 18,763 segments. **b**. Quantile-quantile plot of the segments GC content v.s. PAM content distributions, where the red circles show the top 2,000 PAM segments, and the dashed line represents y = x. **c**. Hexbin plot of segments GC v.s. PAM content, where the color represents the count of points in each hexagon. GC and PAM content is normalized to the 0-1 range.

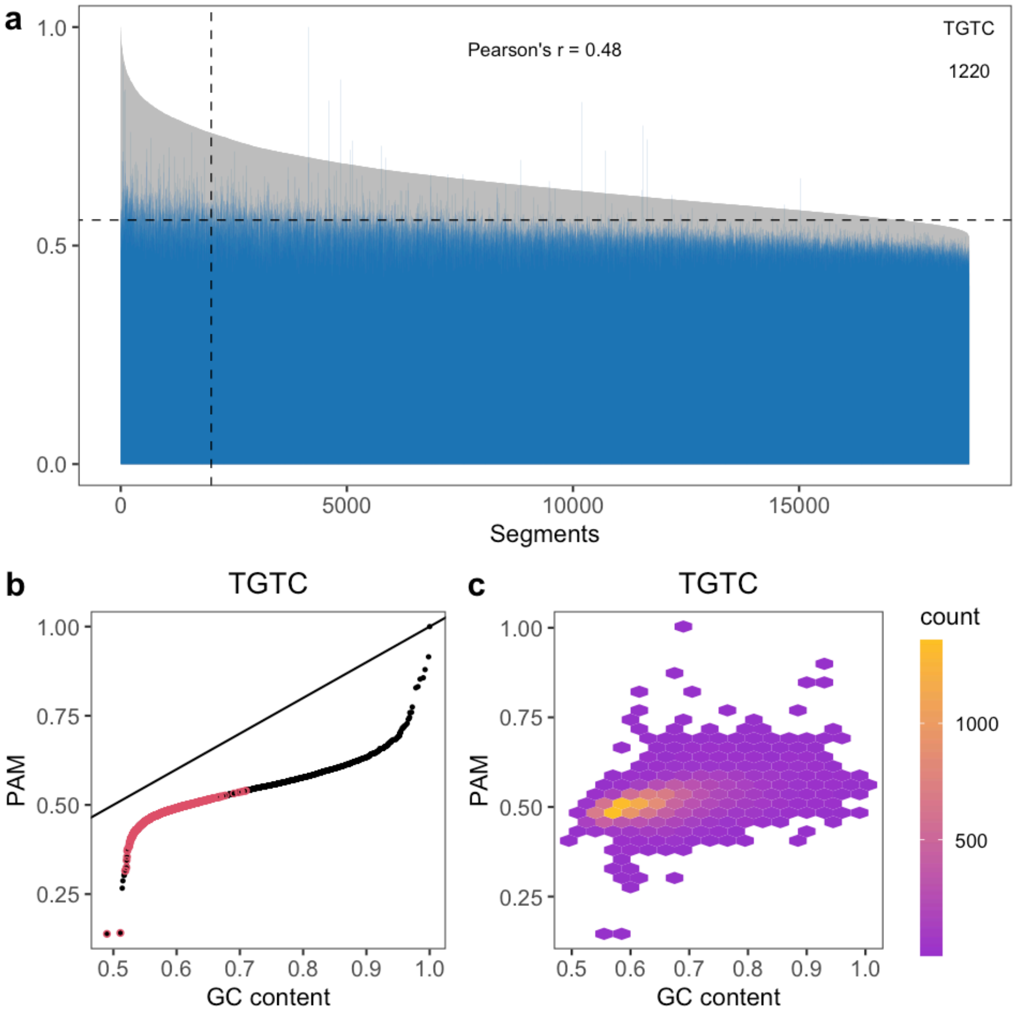

Supplementary Figure 63. Distribution of the TGTC PAM enriched regions compared with that of the GC-rich regions. **a**. PAM (shown on the top-right) content of 18,763 segments (excluding 1,237 segments with all N bases), respectively. The genome segments are ordered by decreasing GC content (grey), and the corresponding PAM content is plotted in the same order (blue). The segments to the left of the vertical dashed line are the top 2,000 segments for GC content, and the segments above the horizontal dashed line are the top 2,000 segments for the TGTC PAM. The number below the name of PAM (top-right) shows the number out of the top 2,000 PAM segments that are not in the top 2,000 GC-rich segments. The Pearson's r is the correlation between the GC content and the PAM content in all 18,763 segments. **b**. Quantile-quantile plot of the segments GC content v.s. PAM content distributions, where the red circles show the top 2,000 PAM segments, and the dashed line represents y = x. **c**. Hexbin plot of segments GC v.s. PAM content, where the color represents the count of points in each hexagon. GC and PAM content is normalized to the 0-1 range.

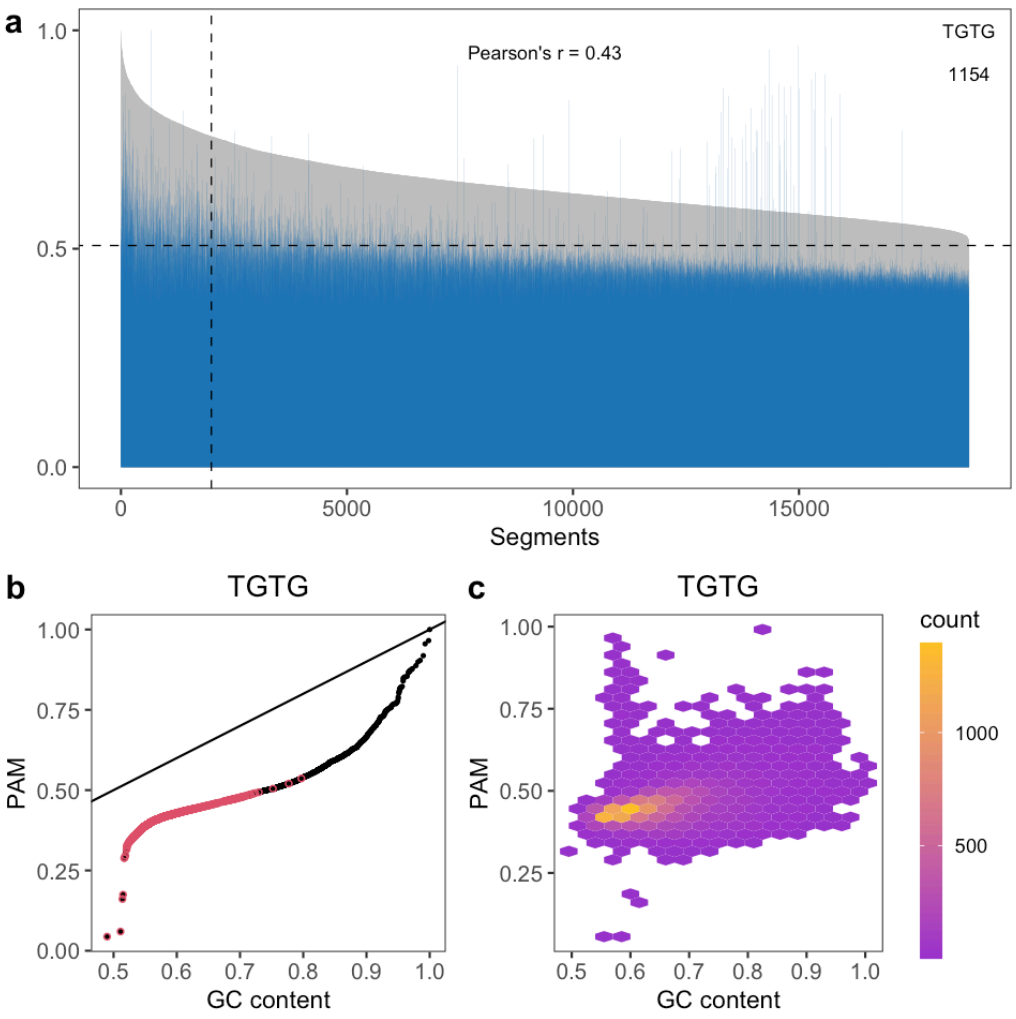

Supplementary Figure 64. Distribution of the TGTG PAM enriched regions compared with that of the GC-rich regions. **a**. PAM (shown on the top-right) content of 18,763 segments (excluding 1,237 segments with all N bases), respectively. The genome segments are ordered by decreasing GC content (grey), and the corresponding PAM content is plotted in the same order (blue). The segments to the left of the vertical dashed line are the top 2,000 segments for GC content, and the segments above the horizontal dashed line are the top 2,000 segments for the TGTG PAM. The number below the name of PAM (top-right) shows the number out of the top 2,000 PAM segments that are not in the top 2,000 GC-rich segments. The Pearson's r is the correlation between the GC content and the PAM content in all 18,763 segments. **b**. Quantile-quantile plot of the segments GC content v.s. PAM content distributions, where the red circles show the top 2,000 PAM segments, and the dashed line represents y = x. **c**. Hexbin plot of segments GC v.s. PAM content, where the color represents the count of points in each hexagon. GC and PAM content is normalized to the 0-1 range.

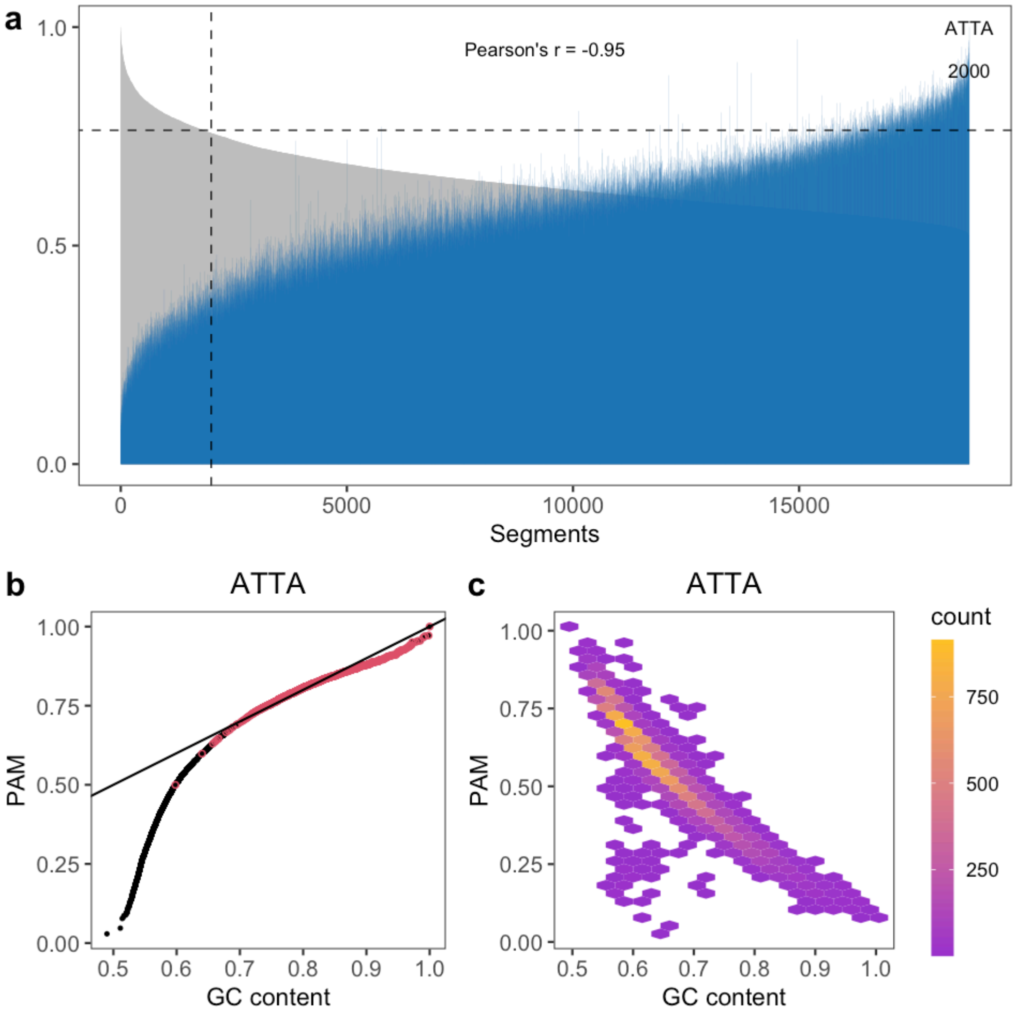

Supplementary Figure 65. Distribution of the ATTA PAM enriched regions compared with that of the GC-rich regions. **a**. PAM (shown on the top-right) content of 18,763 segments (excluding 1,237 segments with all N bases), respectively. The genome segments are ordered by decreasing GC content (grey), and the corresponding PAM content is plotted in the same order (blue). The segments to the left of the vertical dashed line are the top 2,000 segments for GC content, and the segments above the horizontal dashed line are the top 2,000 segments for the ATTA PAM. The number below the name of PAM (top-right) shows the number out of the top 2,000 PAM segments that are not in the top 2,000 GC-rich segments. The Pearson's r is the correlation between the GC content and the PAM content in all 18,763 segments. **b**. Quantile-quantile plot of the segments GC content v.s. PAM content distributions, where the red circles show the top 2,000 PAM segments, and the dashed line represents y = x. **c**. Hexbin plot of segments GC v.s. PAM content, where the color represents the count of points in each hexagon. GC and PAM content is normalized to the 0-1 range.

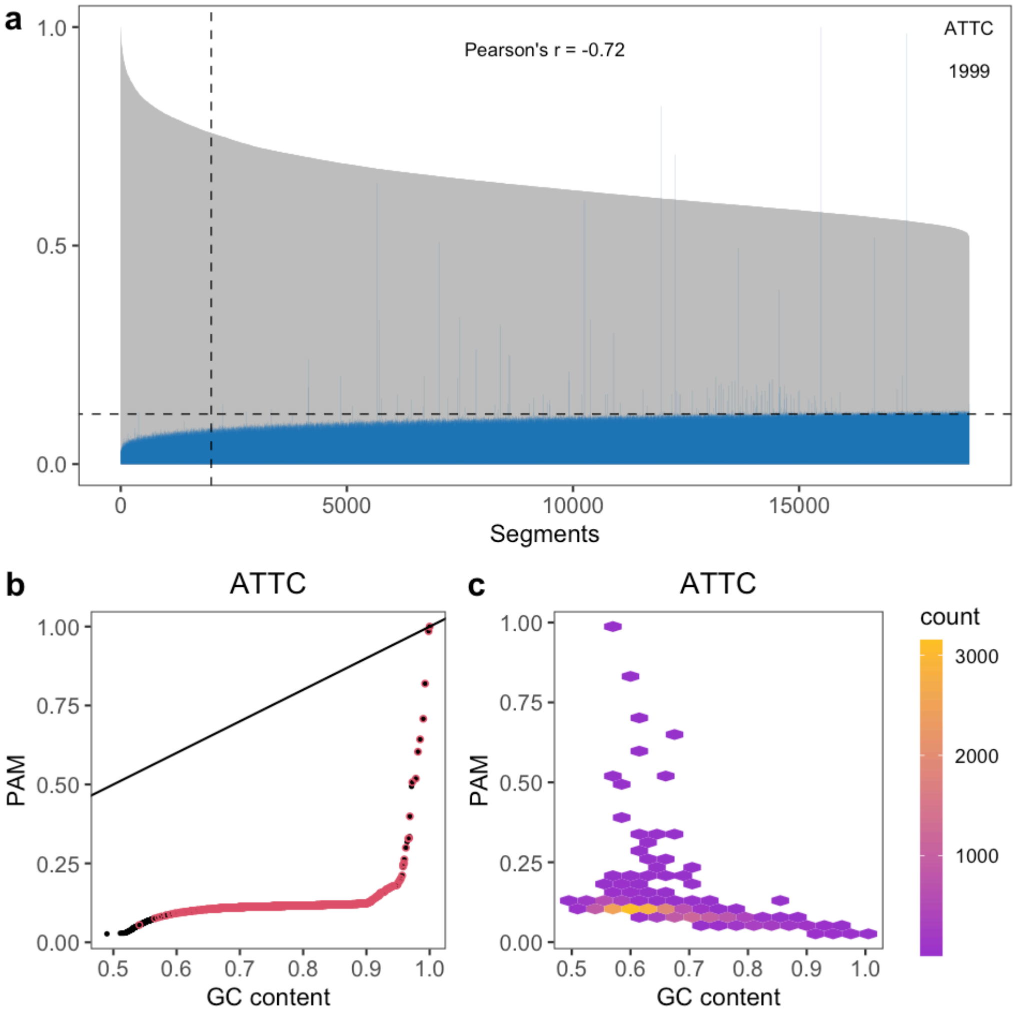

Supplementary Figure 66. Distribution of the ATTC PAM enriched regions compared with that of the GC-rich regions. **a**. PAM (shown on the top-right) content of 18,763 segments (excluding 1,237 segments with all N bases), respectively. The genome segments are ordered by decreasing GC content (grey), and the corresponding PAM content is plotted in the same order (blue). The segments to the left of the vertical dashed line are the top 2,000 segments for GC content, and the segments above the horizontal dashed line are the top 2,000 segments for the ATTC PAM. The number below the name of PAM (top-right) shows the number out of the top 2,000 PAM segments that are not in the top 2,000 GC-rich segments. The Pearson's r is the correlation between the GC content and the PAM content in all 18,763 segments. **b**. Quantile-quantile plot of the segments GC content v.s. PAM content distributions, where the red circles show the top 2,000 PAM segments, and the dashed line represents y = x. **c**. Hexbin plot of segments GC v.s. PAM content, where the color represents the count of points in each hexagon. GC and PAM content is normalized to the 0-1 range.

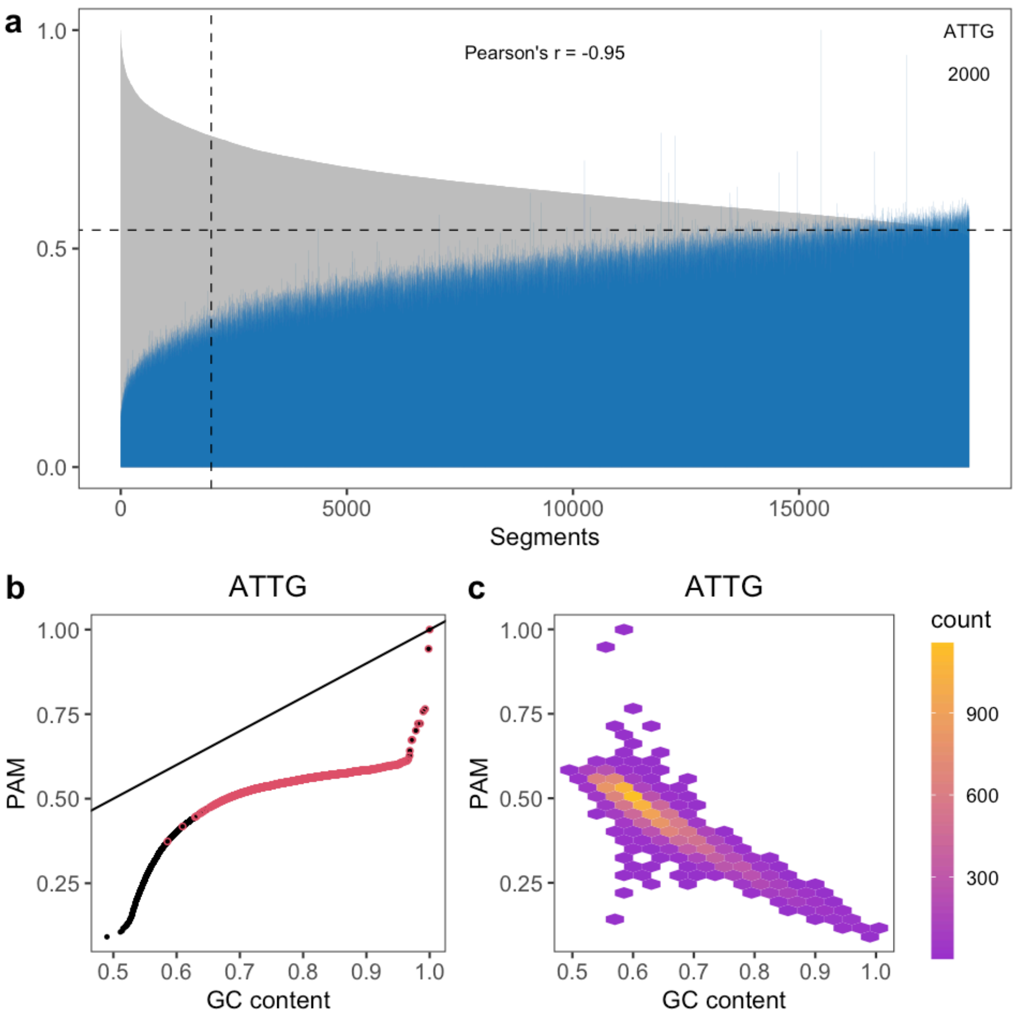

Supplementary Figure 67. Distribution of the ATTG PAM enriched regions compared with that of the GC-rich regions. **a**. PAM (shown on the top-right) content of 18,763 segments (excluding 1,237 segments with all N bases), respectively. The genome segments are ordered by decreasing GC content (grey), and the corresponding PAM content is plotted in the same order (blue). The segments to the left of the vertical dashed line are the top 2,000 segments for GC content, and the segments above the horizontal dashed line are the top 2,000 segments for the ATTG PAM. The number below the name of PAM (top-right) shows the number out of the top 2,000 PAM segments that are not in the top 2,000 GC-rich segments. The Pearson's r is the correlation between the GC content and the PAM content in all 18,763 segments. **b**. Quantile-quantile plot of the segments GC content v.s. PAM content distributions, where the red circles show the top 2,000 PAM segments, and the dashed line represents y = x. **c**. Hexbin plot of segments GC v.s. PAM content, where the color represents the count of points in each hexagon. GC and PAM content is normalized to the 0-1 range.

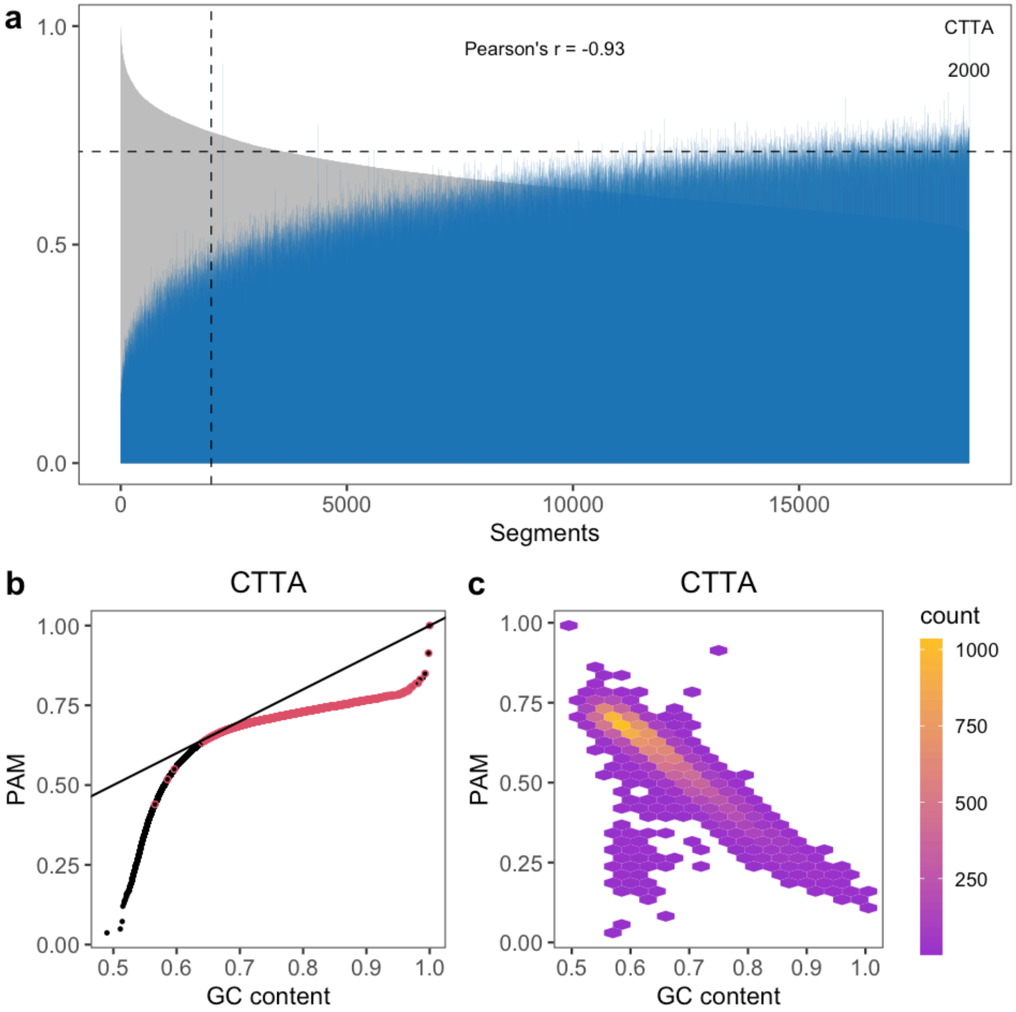

Supplementary Figure 68. Distribution of the CTTA PAM enriched regions compared with that of the GC-rich regions. **a**. PAM (shown on the top-right) content of 18,763 segments (excluding 1,237 segments with all N bases), respectively. The genome segments are ordered by decreasing GC content (grey), and the corresponding PAM content is plotted in the same order (blue). The segments to the left of the vertical dashed line are the top 2,000 segments for GC content, and the segments above the horizontal dashed line are the top 2,000 segments for the CTTA PAM. The number below the name of PAM (top-right) shows the number out of the top 2,000 PAM segments that are not in the top 2,000 GC-rich segments. The Pearson's r is the correlation between the GC content and the PAM content in all 18,763 segments. **b**. Quantile-quantile plot of the segments GC content v.s. PAM content distributions, where the red circles show the top 2,000 PAM segments, and the dashed line represents y = x. **c**. Hexbin plot of segments GC v.s. PAM content, where the color represents the count of points in each hexagon. GC and PAM content is normalized to the 0-1 range.

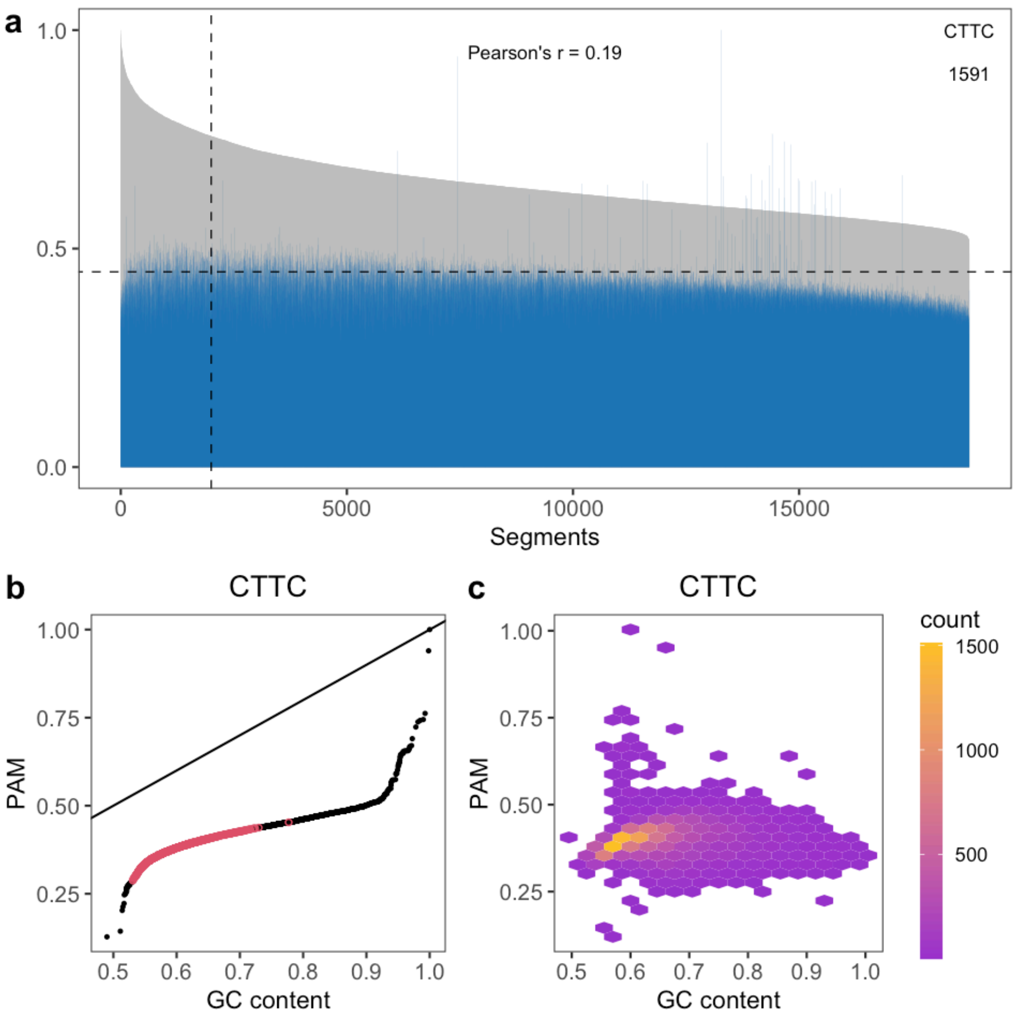

Supplementary Figure 69. Distribution of the CTTC PAM enriched regions compared with that of the GC-rich regions. **a**. PAM (shown on the top-right) content of 18,763 segments (excluding 1,237 segments with all N bases), respectively. The genome segments are ordered by decreasing GC content (grey), and the corresponding PAM content is plotted in the same order (blue). The segments to the left of the vertical dashed line are the top 2,000 segments for GC content, and the segments above the horizontal dashed line are the top 2,000 segments for the CTTC PAM. The number below the name of PAM (top-right) shows the number out of the top 2,000 PAM segments that are not in the top 2,000 GC-rich segments. The Pearson's r is the correlation between the GC content and the PAM content in all 18,763 segments. **b**. Quantile-quantile plot of the segments GC content v.s. PAM content distributions, where the red circles show the top 2,000 PAM segments, and the dashed line represents y = x. **c**. Hexbin plot of segments GC v.s. PAM content, where the color represents the count of points in each hexagon. GC and PAM content is normalized to the 0-1 range.

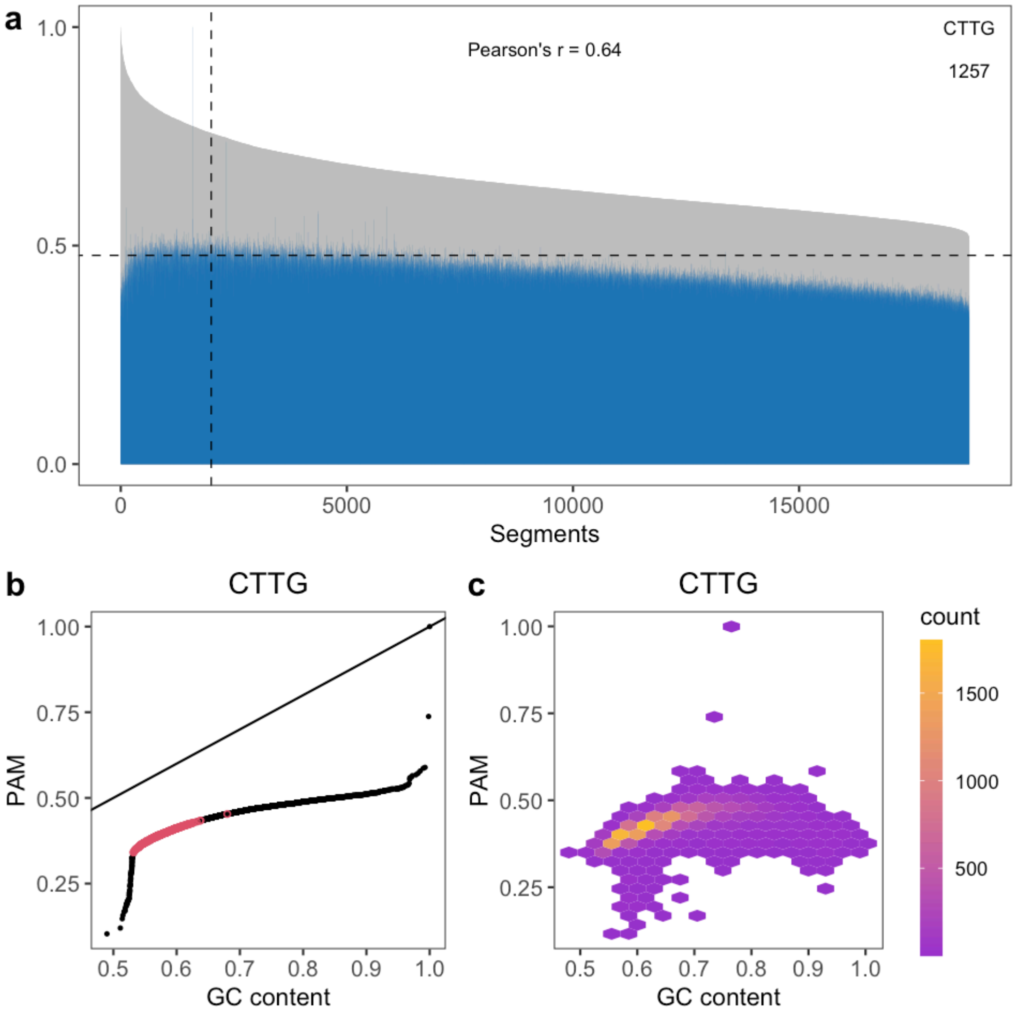

Supplementary Figure 70. Distribution of the CTTG PAM enriched regions compared with that of the GC-rich regions. **a**. PAM (shown on the top-right) content of 18,763 segments (excluding 1,237 segments with all N bases), respectively. The genome segments are ordered by decreasing GC content (grey), and the corresponding PAM content is plotted in the same order (blue). The segments to the left of the vertical dashed line are the top 2,000 segments for GC content, and the segments above the horizontal dashed line are the top 2,000 segments for the CTTG PAM. The number below the name of PAM (top-right) shows the number out of the top 2,000 PAM segments that are not in the top 2,000 GC-rich segments. The Pearson's r is the correlation between the GC content and the PAM content in all 18,763 segments. **b**. Quantile-quantile plot of the segments GC content v.s. PAM content distributions, where the red circles show the top 2,000 PAM segments, and the dashed line represents y = x. **c**. Hexbin plot of segments GC v.s. PAM content, where the color represents the count of points in each hexagon. GC and PAM content is normalized to the 0-1 range.

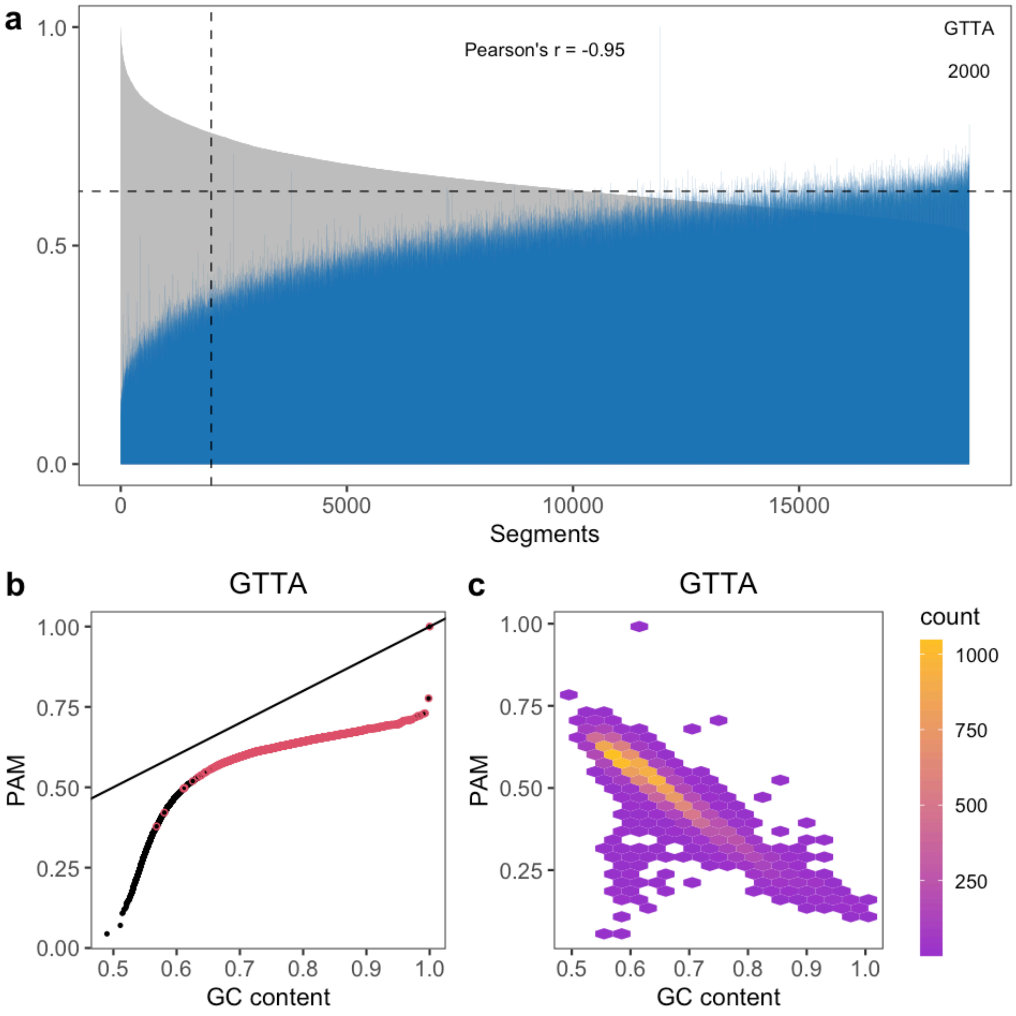

Supplementary Figure 71. Distribution of the GTTA PAM enriched regions compared with that of the GC-rich regions. **a**. PAM (shown on the top-right) content of 18,763 segments (excluding 1,237 segments with all N bases), respectively. The genome segments are ordered by decreasing GC content (grey), and the corresponding PAM content is plotted in the same order (blue). The segments to the left of the vertical dashed line are the top 2,000 segments for GC content, and the segments above the horizontal dashed line are the top 2,000 segments for the GTTA PAM. The number below the name of PAM (top-right) shows the number out of the top 2,000 PAM segments that are not in the top 2,000 GC-rich segments. The Pearson's r is the correlation between the GC content and the PAM content in all 18,763 segments. **b**. Quantile-quantile plot of the segments GC content v.s. PAM content distributions, where the red circles show the top 2,000 PAM segments, and the dashed line represents y = x. **c**. Hexbin plot of segments GC v.s. PAM content, where the color represents the count of points in each hexagon. GC and PAM content is normalized to the 0-1 range.

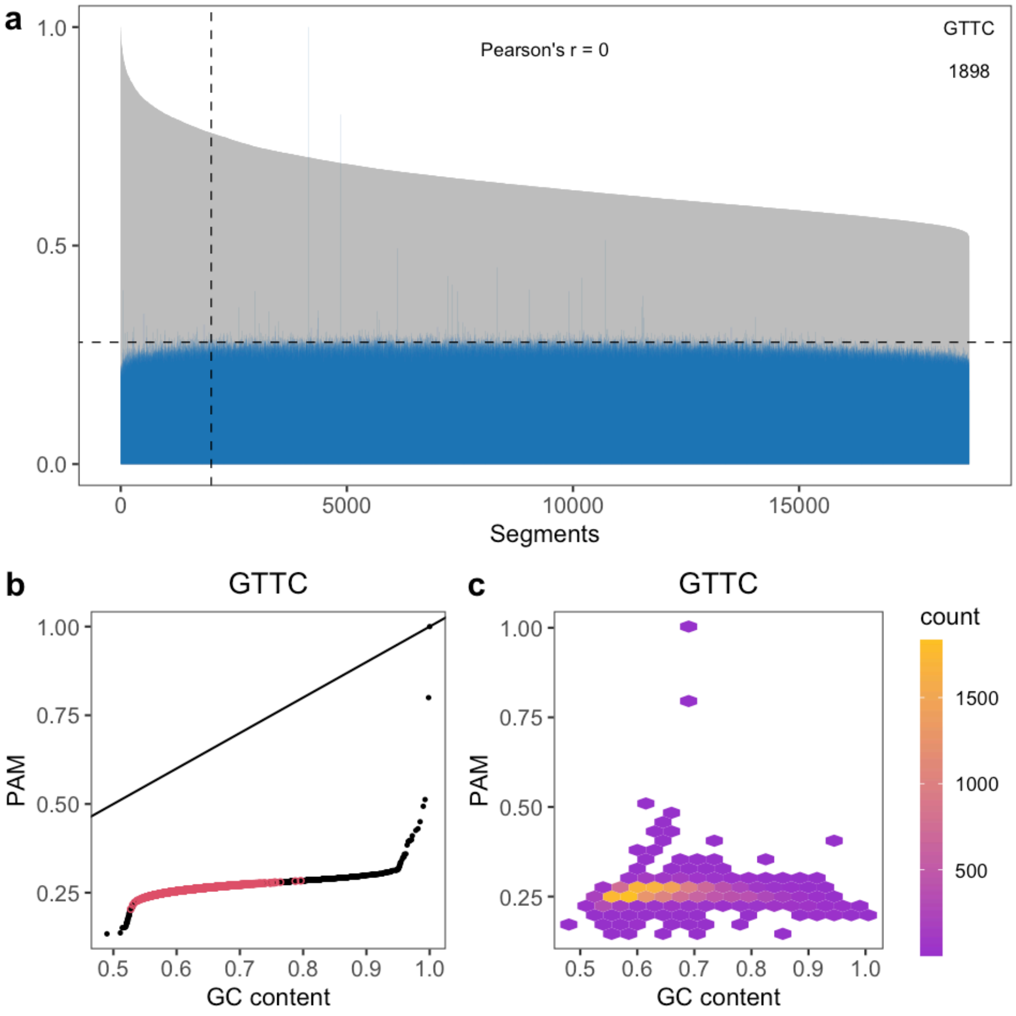

Supplementary Figure 72. Distribution of the GTTC PAM enriched regions compared with that of the GC-rich regions. **a**. PAM (shown on the top-right) content of 18,763 segments (excluding 1,237 segments with all N bases), respectively. The genome segments are ordered by decreasing GC content (grey), and the corresponding PAM content is plotted in the same order (blue). The segments to the left of the vertical dashed line are the top 2,000 segments for GC content, and the segments above the horizontal dashed line are the top 2,000 segments for the GTTC PAM. The number below the name of PAM (top-right) shows the number out of the top 2,000 PAM segments that are not in the top 2,000 GC-rich segments. The Pearson's r is the correlation between the GC content and the PAM content in all 18,763 segments. **b**. Quantile-quantile plot of the segments GC content v.s. PAM content distributions, where the red circles show the top 2,000 PAM segments, and the dashed line represents y = x. **c**. Hexbin plot of segments GC v.s. PAM content, where the color represents the count of points in each hexagon. GC and PAM content is normalized to the 0-1 range.

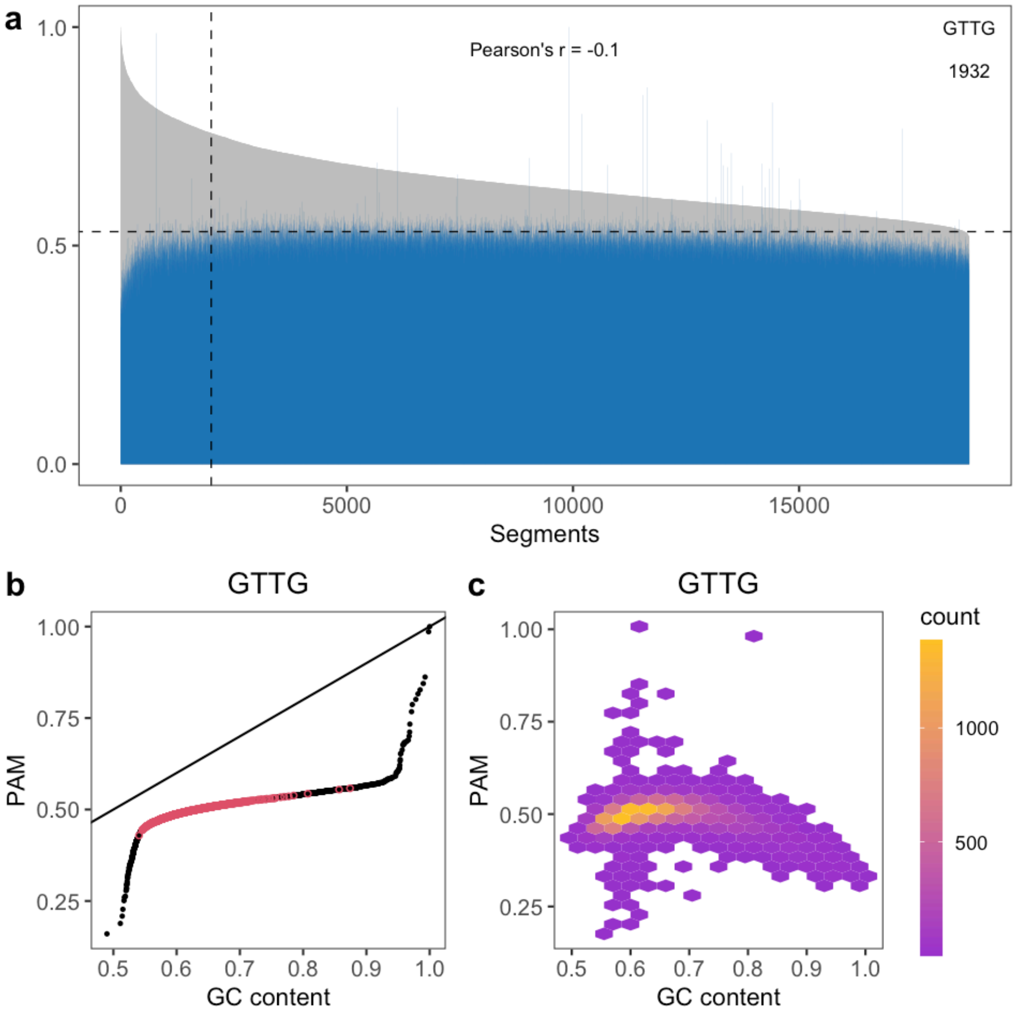

Supplementary Figure 73. Distribution of the GTTG PAM enriched regions compared with that of the GC-rich regions. **a**. PAM (shown on the top-right) content of 18,763 segments (excluding 1,237 segments with all N bases), respectively. The genome segments are ordered by decreasing GC content (grey), and the corresponding PAM content is plotted in the same order (blue). The segments to the left of the vertical dashed line are the top 2,000 segments for GC content, and the segments above the horizontal dashed line are the top 2,000 segments for the GTTG PAM. The number below the name of PAM (top-right) shows the number out of the top 2,000 PAM segments that are not in the top 2,000 GC-rich segments. The Pearson's r is the correlation between the GC content and the PAM content in all 18,763 segments. **b**. Quantile-quantile plot of the segments GC content v.s. PAM content distributions, where the red circles show the top 2,000 PAM segments, and the dashed line represents y = x. **c**. Hexbin plot of segments GC v.s. PAM content, where the color represents the count of points in each hexagon. GC and PAM content is normalized to the 0-1 range.

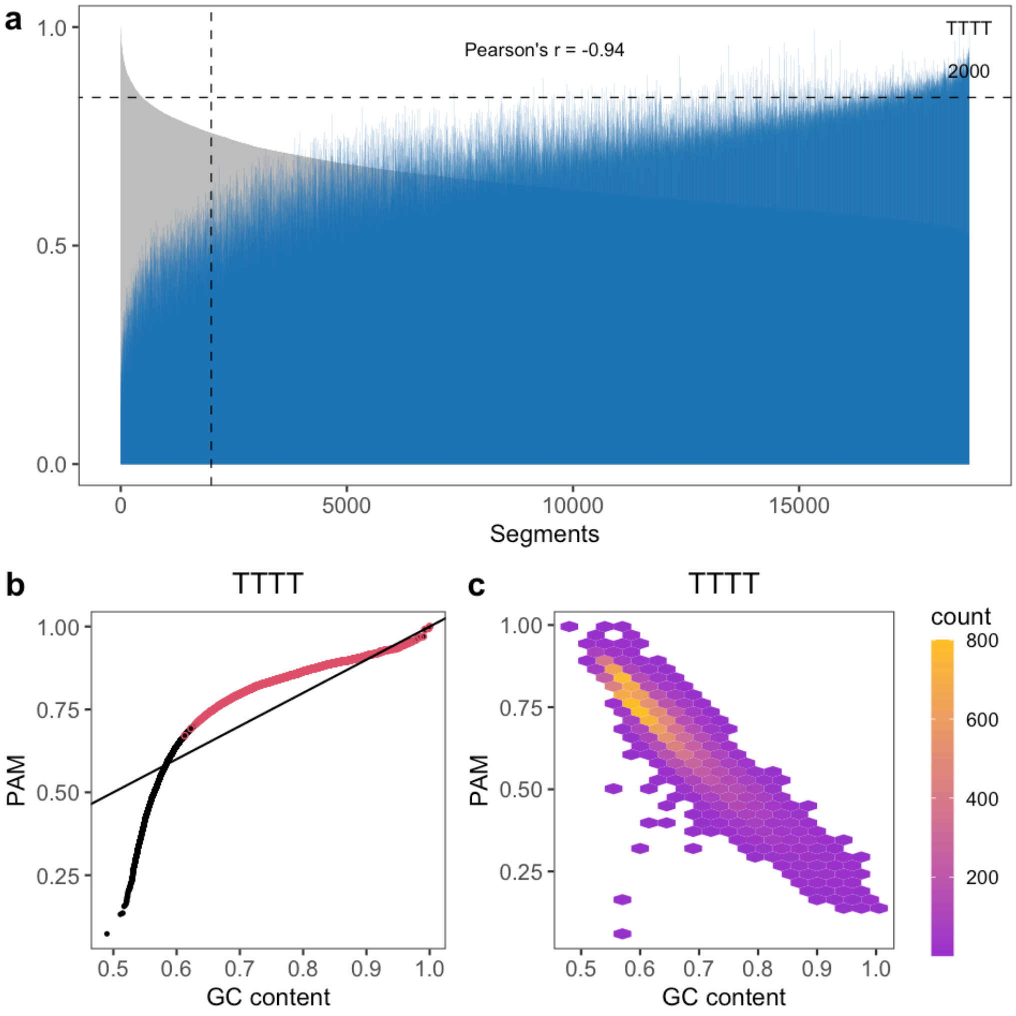

Supplementary Figure 74. Distribution of the TTTT PAM enriched regions compared with that of the GC-rich regions. **a**. PAM (shown on the top-right) content of 18,763 segments (excluding 1,237 segments with all N bases), respectively. The genome segments are ordered by decreasing GC content (grey), and the corresponding PAM content is plotted in the same order (blue). The segments to the left of the vertical dashed line are the top 2,000 segments for GC content, and the segments above the horizontal dashed line are the top 2,000 segments for the TTTT PAM. The number below the name of PAM (top-right) shows the number out of the top 2,000 PAM segments that are not in the top 2,000 GC-rich segments. The Pearson's r is the correlation between the GC content and the PAM content in all 18,763 segments. **b**. Quantile-quantile plot of the segments GC content v.s. PAM content distributions, where the red circles show the top 2,000 PAM segments, and the dashed line represents y = x. **c**. Hexbin plot of segments GC v.s. PAM content, where the color represents the count of points in each hexagon. GC and PAM content is normalized to the 0-1 range.

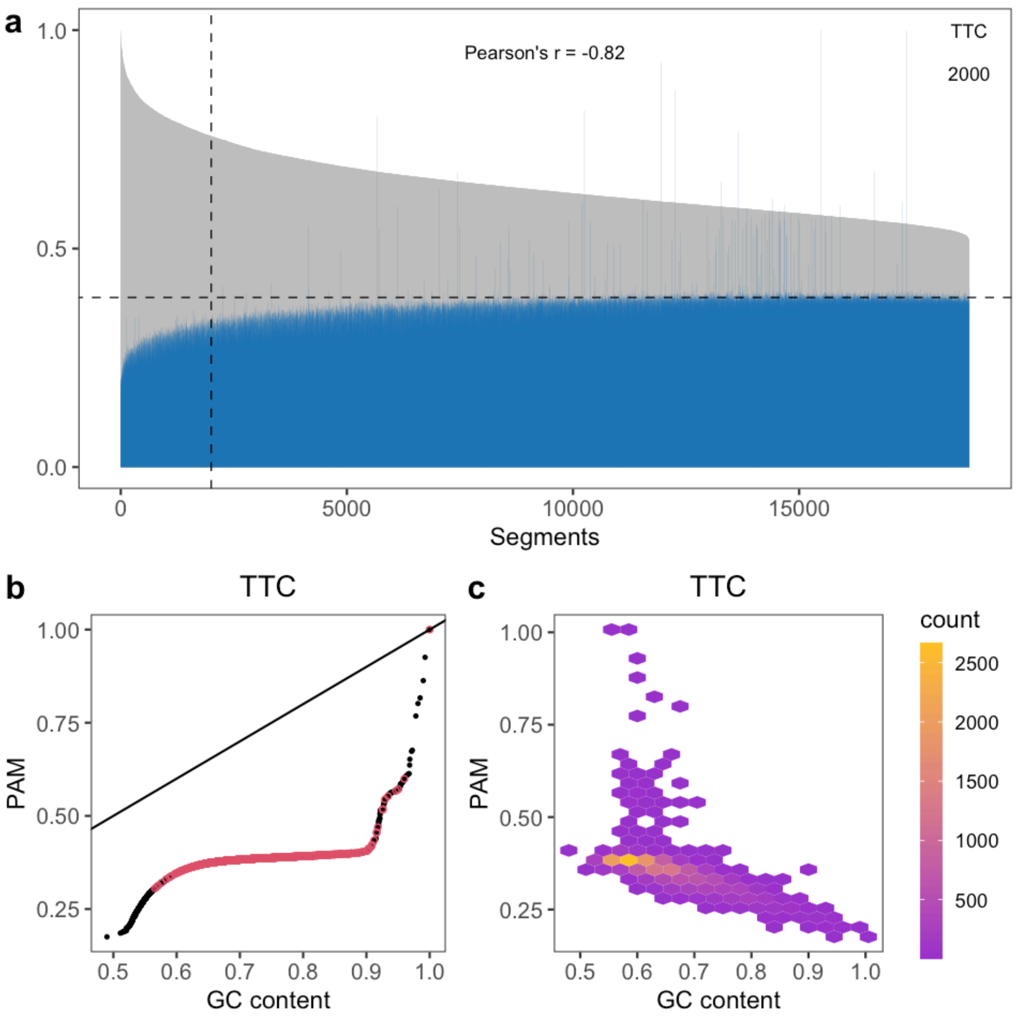

Supplementary Figure 75. Distribution of the TTC PAM enriched regions compared with that of the GC-rich regions. **a**. PAM (shown on the top-right) content of 18,763 segments (excluding 1,237 segments with all N bases), respectively. The genome segments are ordered by decreasing GC content (grey), and the corresponding PAM content is plotted in the same order (blue). The segments to the left of the vertical dashed line are the top 2,000 segments for GC content, and the segments above the horizontal dashed line are the top 2,000 segments for the TTC PAM. The number below the name of PAM (top-right) shows the number out of the top 2,000 PAM segments that are not in the top 2,000 GC-rich segments. The Pearson's r is the correlation between the GC content and the PAM content in all 18,763 segments. **b**. Quantile-quantile plot of the segments GC content v.s. PAM content distributions, where the red circles show the top 2,000 PAM segments, and the dashed line represents y = x. **c**. Hexbin plot of segments GC v.s. PAM content, where the color represents the count of points in each hexagon. GC and PAM content is normalized to the 0-1 range.

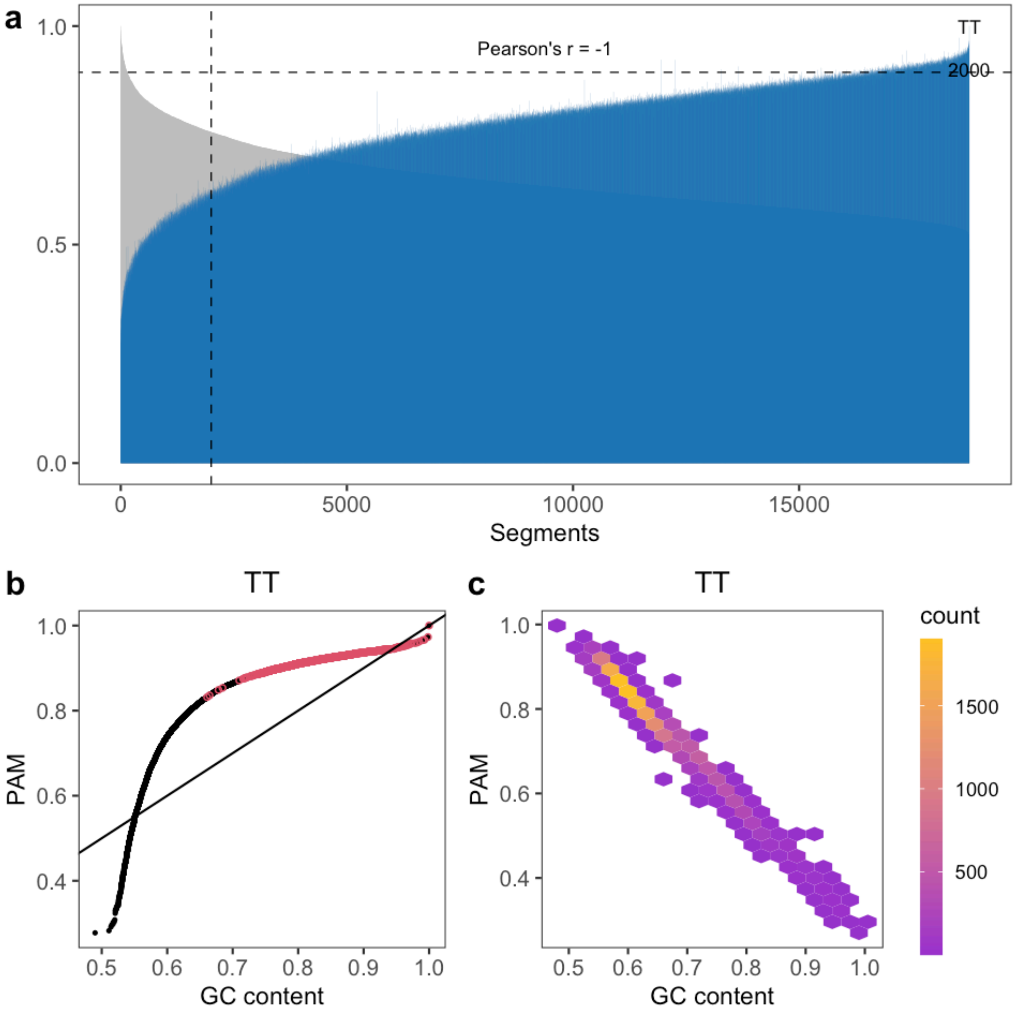

Supplementary Figure 76. Distribution of the TT PAM enriched regions compared with that of the GC-rich regions. **a**. PAM (shown on the top-right) content of 18,763 segments (excluding 1,237 segments with all N bases), respectively. The genome segments are ordered by decreasing GC content (grey), and the corresponding PAM content is plotted in the same order (blue). The segments to the left of the vertical dashed line are the top 2,000 segments for GC content, and the segments above the horizontal dashed line are the top 2,000 segments for the TT PAM. The number below the name of PAM (top-right) shows the number out of the top 2,000 PAM segments that are not in the top 2,000 GC-rich segments. The Pearson's r is the correlation between the GC content and the PAM content in all 18,763 segments. **b**. Quantile-quantile plot of the segments GC content v.s. PAM content distributions, where the red circles show the top 2,000 PAM segments, and the dashed line represents y = x. **c**. Hexbin plot of segments GC v.s. PAM content, where the color represents the count of points in each hexagon. GC and PAM content is normalized to the 0-1 range.

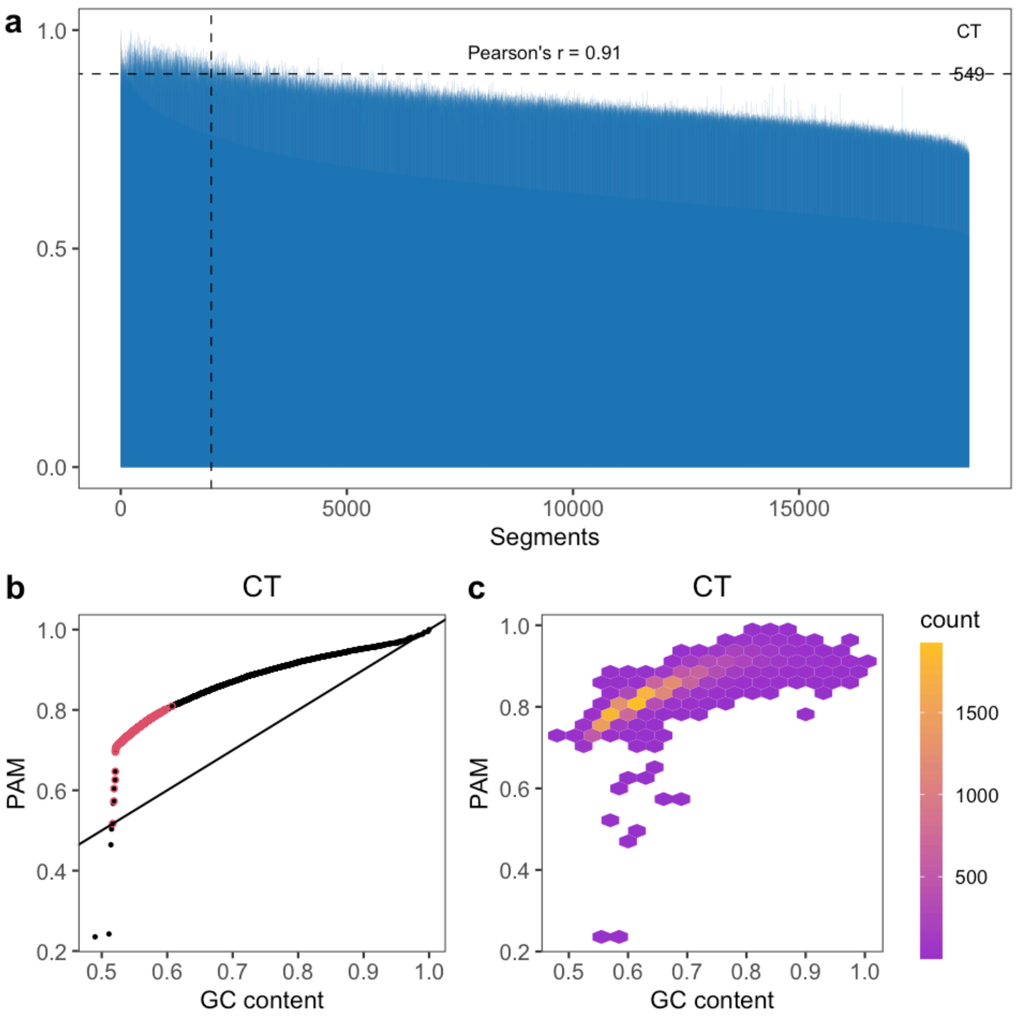

Supplementary Figure 77. Distribution of the CT PAM enriched regions compared with that of the GC-rich regions. **a**. PAM (shown on the top-right) content of 18,763 segments (excluding 1,237 segments with all N bases), respectively. The genome segments are ordered by decreasing GC content (grey), and the corresponding PAM content is plotted in the same order (blue). The segments to the left of the vertical dashed line are the top 2,000 segments for GC content, and the segments above the horizontal dashed line are the top 2,000 segments for the CT PAM. The number below the name of PAM (top-right) shows the number out of the top 2,000 PAM segments that are not in the top 2,000 GC-rich segments. The Pearson's r is the correlation between the GC content and the PAM content in all 18,763 segments. **b**. Quantile-quantile plot of the segments GC content v.s. PAM content distributions, where the red circles show the top 2,000 PAM segments, and the dashed line represents y = x. **c**. Hexbin plot of segments GC v.s. PAM content, where the color represents the count of points in each hexagon. GC and PAM content is normalized to the 0-1 range.

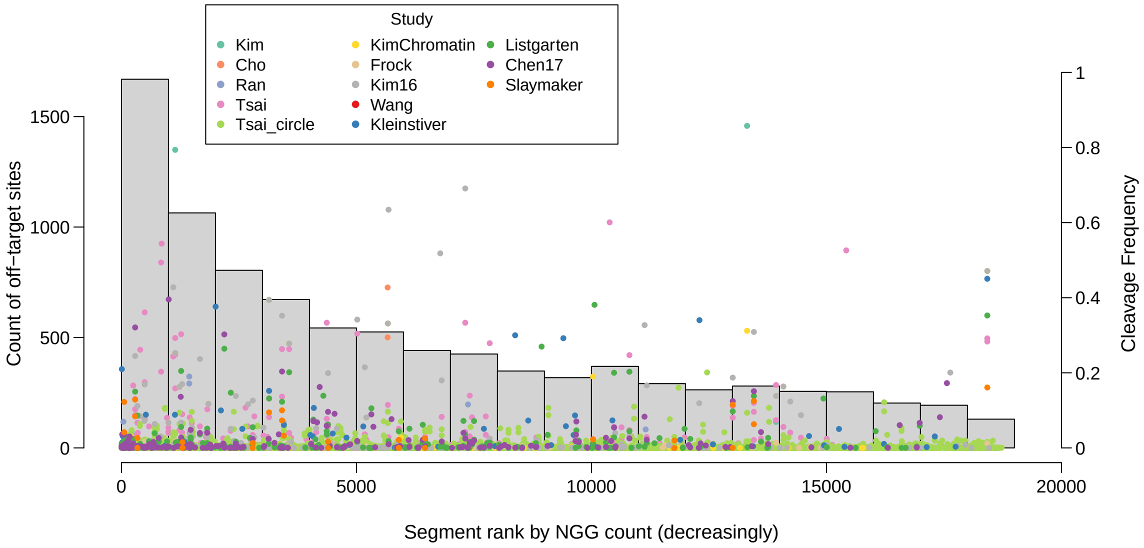

Supplementary Figure 78. Comparison between SpCas9 off-target sites frequency and NGG count of 20,000 segments. Each of 8956 off-target sites is matched to one of 20,000 segments that we defined in the analysis. The x axis is the segment rank by NGG count decreasingly. The histogram shows the distribution of matched segments by the off-target sites, with the y axis named 'Count of off-target sites' (left). The scatter plot shows the cleavage frequency of the corresponding off-target sites, with the y axis named 'Cleavage Frequency' (right).
