# Supplementary material for: Contribution of CRISPRable DNA to human complex traits
Source: Commun Biol. 2022 Oct 20;5:1111. doi: 10.1038/s42003-022-03969-7 (PMC9585070; doi:10.1038/s42003-022-03969-7)
Supplement: Supplementary file 3 — Description of Additional Supplementary Files [file 42003_2022_3969_MOESM3_ESM.pdf]

Description of Additional Supplementary Files

for

Contribution of CRISPRable DNA to human complex traits

Filename: Supplementary Data 1
Description: CRISPR-Cas enzymes and their PAMs. This table summarized 77 unique PAMs for 21 Cas enzymes and their corresponding publications. Class (type): Cas enzyme classification, Cas9 or Cas12a in this article; Cas: Cas enzyme names; PAM (IUPAC code): PAM sequence using IUPAC nucleotide code names; PAM: PAM sequence using nucleotide names; non-N PAM: PAM sequence without the N at the start; non-N rPAM: non-N PAM on the reverse strand; overall GC content: the overall GC content for each Cas, blue indicates Cas9 and green indicates Cas12a, shades of color represent the overall GC content; Reference (in article): reference number appeared in the article; Reference: the publication we referred to in order to determine the PAM. When counting the number of the PAM sequence, we are referring to the number of both non-N PAM and non-N rPAM.

Filename: Supplementary Data 2
Description: Summary of 28 human complex traits. This table described 28 complex traits that were used in our S-LDSC heritability partitioning analysis. Category: category of the trait; Trait: trait we used, ordered alphabetically within each category; h^2 (standard error): the heritability (h^2) and its standard error estimated by LDSC; Consortium: consortium that the trait belong to, "-" means the trait belong to no consortium; Reference: publication that provided the summary statistics.

Filename: Supplementary Data 3
Description: Heritability enrichment of Cas annotations on 28 complex traits. This table corresponds to the heatmap in **Fig. 1**. This table also includes other information of our Cas annotations, for example, the proportion of SNP that each annotation contains (the Prop._SNP column) and P value (the Enrichment_p column) and the P value after false discovery rate correction (the FDR column).

Filename: Supplementary Data 4
Description: Functional annotations that overlap with our PAM annotations.} This table shows the details of our functional annotations that overlap with our Cas annotations. LDSC names: functional annotation names provided by the LDSC software; Name: Names we used in the article and **Fig. 2**; Description: detailed description for the functional annotations, some provided with their publications.
